# High entropy liquid electrolytes for lithium batteries

**DOI:** 10.1038/s41467-023-36075-1

**Published:** 2023-01-27

**Authors:** Qidi Wang, Chenglong Zhao, Jianlin Wang, Zhenpeng Yao, Shuwei Wang, Sai Govind Hari Kumar, Swapna Ganapathy, Stephen Eustace, Xuedong Bai, Baohua Li, Marnix Wagemaker

**Affiliations:** 1 Department of Radiation Science and Technology, Delft University of Technology, Delft, 2629JB Netherlands; 2 State Key Laboratory for Surface Physics, Institute of Physics, Chinese Academy of Sciences, Beijing, 100190 China; 3 The State Key Laboratory of Metal Matrix Composites, School of Materials Science and Engineering, Shanghai Jiao Tong University, Shanghai, 200240 China; 4 Center of Hydrogen Science, Shanghai Jiao Tong University, Shanghai, 200240 China; 5 Shenzhen Key Laboratory on Power Battery Safety and Shenzhen Geim Graphene Center, School of Shenzhen International Graduate School, Tsinghua University, Guangdong, 518055 China; 6 Department of Chemistry and Computer Science, University of Toronto, Toronto, ON M5S 3H6 Canada; 7 Department of Biotechnology, Delft University of Technology, Delft, 2629HZ Netherlands

**Keywords:** Batteries, Batteries, Electrochemistry, Batteries

## Abstract

High-entropy alloys/compounds have large configurational entropy by introducing multiple components, showing improved functional properties that exceed those of conventional materials. However, how increasing entropy impacts the thermodynamic/kinetic properties in liquids that are ambiguous. Here we show this strategy in liquid electrolytes for rechargeable lithium batteries, demonstrating the substantial impact of raising the entropy of electrolytes by introducing multiple salts. Unlike all liquid electrolytes so far reported, the participation of several anionic groups in this electrolyte induces a larger diversity in solvation structures, unexpectedly decreasing solvation strengths between lithium ions and solvents/anions, facilitating lithium-ion diffusivity and the formation of stable interphase passivation layers. In comparison to the single-salt electrolytes, a low-concentration dimethyl ether electrolyte with four salts shows an enhanced cycling stability and rate capability. These findings, rationalized by the fundamental relationship between entropy-dominated solvation structures and ion transport, bring forward high-entropy electrolytes as a composition-rich and unexplored space for lithium batteries and beyond.

## Abstract (web summary)

Electrolytes, function as an ion conducting membrane between battery electrodes, are essential for rechargeable batteries. Here, the authors report high-entropy liquid electrolytes and reveal substantial impact of the increasing entropy on lithium-ion solvation structures for highly reversible lithium batteries.

## Introduction

High-entropy (HE) alloys have attracted significant attention in the fields of materials science and engineering since the introduction from 2004^1,2^, because of their potentially desirable properties^3–6^. Several structural, thermodynamic, and kinetic principles have been proposed to demonstrate the special nature of such materials. Firstly, the presence of several principal elements, typically more than five, can promote the formation of solid-solution phases. On the other hand, the distortion of the local lattice due to the configurational disorder can lead to improved mechanical properties^4^. Another contributor to the improved functional properties of HE alloys is suggested to be the different diffusion kinetics^7^, which however is subject to debate, as experimental studies are rare and complex, and the state of knowledge is still far from complete^8,9^.

**Fig. 1:**
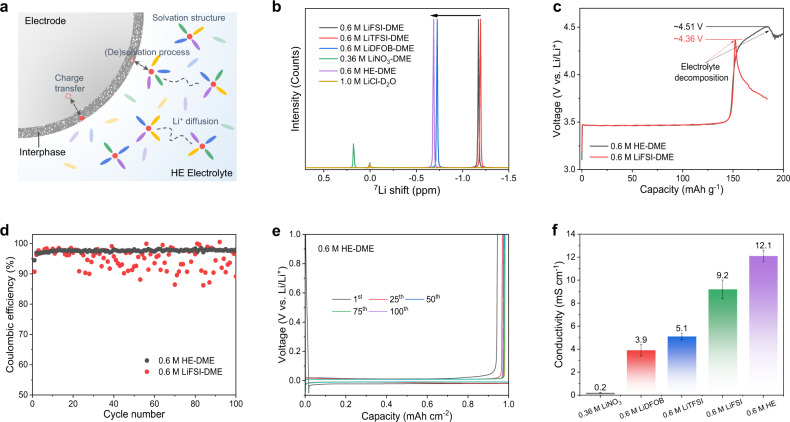
Properties of the HE liquid electrolyte. **a** Schematic of the HE electrolyte battery system. **b** ^7^Li NMR spectra of single-salt electrolytes and the as-prepared HE electrolyte. Due to the relatively low salt solubility of LiNO_3_ in DME, a 0.36 M LiNO_3_-DME electrolyte was prepared for comparison. **c** Galvanostatic charge profiles of Li||LiFePO_4_ cells with different electrolytes at a current density of 0.02 C. **d** Lithium plating/stripping CE in Li||Cu cells using 0.6 M LiFSI-DME and 0.6 M HE-DME electrolytes. **e** Galvanostatic lithium plating/stripping profiles of 0.6 M HE-DME electrolyte. **f** Comparison of the lithium-ion conductivity of different electrolytes.

A new research direction within the class of HE materials is liquid electrolyte solutions, which function as an ion-conducting membrane between battery electrodes^10,11^. However, the basic properties of HE electrolytes are unexplored to date. This motivates investigation as to how the main electrolyte functional properties are impacted, including redox stability, ion conductivity, charge transfer and solid electrolyte interphase (SEI) formation. An important aspect herein is to determine whether the changes in electrolyte behavior can be ascribed to the larger entropy associated with the presence of multiple principal components, or to the properties of the principle chemical components themselves. By combining multiple salts with a single solvent or/and a single salt with multiple solvents, a more complex and diverse solvation structure is expected to form, which is due to the diversity of local interactions between solvents, lithium-ion and anionic groups^10^. Such complex solvation structure could influence redox stability, charge transfer and the SEI composition and structure (Fig. 1a). These properties determine to a large extent the battery performance parameters such as cycle life and rate performance^12,13^. How entropy impacts the kinetic properties such as diffusivity and conductivity is an intriguing aspect, as entropy is only formally related to the thermodynamic properties. However, for liquids, excess entropy scaling has been proposed and empirically demonstrated to be the relationship between the entropy of the system and its kinetic behavior, suggesting that increasing the entropy can result in improved diffusivity^14,15^, which has never been explored for electrolytes. Due to the potential to be able to tune electrolyte properties through the entropy, and the lack of knowledge thereof, we embark on a systematic study of the properties of HE electrolytes and their impact on the relevant processes in lithium batteries.

Differing from mixing solvents that is a common strategy to compensate deficiencies of the individual solvents, where extra functional solvents are introduced as additives to the corresponding functional properties^16–20^. In this work, we show a prototype HE electrolyte through combining 0.15 mol/L (M) lithium bis(fluorosulfonyl)imide (LiFSI), 0.15 M lithium bis(trifluoromethanesulfonyl)imide (LiTFSI), 0.15 M lithium difluoro(oxalato)borate (LiDFOB) and 0.15 M lithium nitrate (LiNO_3_) in a single solvent of dimethoxyethane (DME) (Supplementary Note 1 and Supplementary Fig. 1). This results in a 0.6 M HE-DME electrolyte, which is compared to each of the single salt electrolytes with the same 0.6 M concentration. Results show that more anionic groups can participate in the solvation structures (similar to highly concentrated electrolytes) in the low salt concentration electrolyte, however, the interactions of lithium ions with both solvent and anionic groups are much weaker in this HE electrolyte, a direct result from the higher disorder, which is never observed in any liquid electrolytes. The consequence is the formation of inorganic-rich and stable interphase layers on the electrodes, responsible for more stable cycling of high-voltage lithium batteries. In addition, the weaker solvation strength facilitates lithium-ion mobility, resulting in substantial improvement in rate capability. This suggests that raising the entropy of mixing through introducing multiple salts in solvent provides a general strategy to tailor the functional properties for the development of electrolytes.

## Results

### Characterization of the electrolytes and compatibility with anodes

The lithium-ion solvation environment of the electrolytes is studied using nuclear magnetic resonance (NMR) spectroscopy (Fig. 1b, Supplementary Figs. 2 and 3), where the chemical shift reflects the shielding of the lithium-ions as a result of the solvation environment. The 0.6 M LiTFSI-DME, 0.6 M LiFSI-DME and 0.6 M LiDFOB-DME single-salt electrolytes result in more negative shifts of −1.19, −1.17, and −0.73 ppm, respectively. In this case, the lithium-ions thus experience relatively strong shielding due to a high electron density, indicating a stronger solvation interaction with both solvent and anions. In contrast, a downfield shift for the 0.6 M HE-DME electrolyte is observed, at −0.68 ppm, demonstrating a relatively lower shielding of the lithium-ions, which may promote lithium-ion diffusivity based on a weaker solvation interaction^21^.

The oxidation and reduction stability of the electrolytes with single salt and different salt combinations are evaluated (Supplementary Figs. 4–9 and Supplementary Note 2). Among the single-salt electrolytes, 0.6 M LiFSI-DME shows the best overall stability, therefore it is selected as the control group in the detailed study of the 0.6 M HE-DME electrolyte (Table 1). The oxidative stability limit is evaluated using Li||LiFePO_4_ cells with a cut-off voltage of 5.0 V, making use of the absence of an oxidation reaction of LiFePO_4_ above ~3.8 V (Fig. 1c). The 0.6 M HE-DME shows an oxidation stable potential up to ~4.51 V, higher than the single salt (4.36 V for 0.6 M LiFSI-DME), where the subsequent capacity increase is suggested to be due to the formation of a cathode electrolyte interphase (CEI) on the surface of the cathode. Under a polarization potential of 4.2 V vs. Li/Li^+^, the 0.6 M HE-DME electrolyte presents a stable anodic current due to the suppressed corrosion of the aluminum foil, in contrast to the 0.6 M LiFSI-DME, which suffers from a rapid dissolution of the aluminum foil (Supplementary Fig. 10). Also, the stability against reduction by lithium metal anode appears to be improved as a Li||Cu cell with the 0.6 M HE-DME exhibits stable lithium metal plating/stripping voltage profiles with a Coulombic efficiency (CE) up to 98.6%, considerably higher than that of 0.6 M LiFSI-DME (Fig. 1d, e and Supplementary Fig. 5). Symmetric Li||Li cells, electrochemical impedance (EIS) and cyclic voltammetry (CV) measurements are performed (Supplementary Figs. 11–19 and Supplementary Note 3), all indicating the improved stability of the 0.6 M HE-DME electrolyte against the lithium anode.

**Table 1:** Components used in the HE-DME electrolyte and their stabilities in each single system

		Properties	
Salt	Solvent	Oxidative stability	Reversible stability
0.6 M LiFSI	DME	Moderate	Good
0.6 M LiTFSI	DME	Good	Poor
0.6 M LiDFOB	DME	Good	Poor
0.36 M LiNO_3_	DME	Poor	Poor
0.15 M [LiFSI, LiTFSI, LiDFOB, LiNO_3_]	DME	Good	Good

Measurement of the lithium-ion transference number and conductivity of the 0.6 M HE-DME electrolyte (Fig. 1f, Supplementary Fig. 20 and Supplementary Table 1), result in 0.46 and ~12.1 mS cm^−1^, respectively. This is higher than that measured for the 0.6 M LiFSI-DME electrolyte (0.39 and 9.6 mS cm^−1^, respectively)^22^. The rate capability of the 0.6 M HE-DME electrolyte is investigated in Li||Li_4_Ti_5_O_12_ cells (Supplementary Figs. 21 and 22), making use of the excellent Li_4_Ti_5_O_12_ rate performance and medium working potential. When the rate is increased to 5.0 C, the capacity retention of the 0.6 M LiFSI-DME electrolyte decreases to ~40 mAh g^−1^, much lower than that of ~115 mAh g^−1^ in 0.6 M HE-DME electrolyte (Supplementary Fig. 21). This improved rate performance can directly be related to the higher transference number and conductivity of the 0.6 M HE-DME electrolyte. It should be emphasized that the conductivity of the 0.6 M HE-DME electrolyte is higher than that of the electrolytes with individual salts, showing that the combination of salts results in a higher diffusivity.

### Compatibility with cathodes

**Fig. 2:**
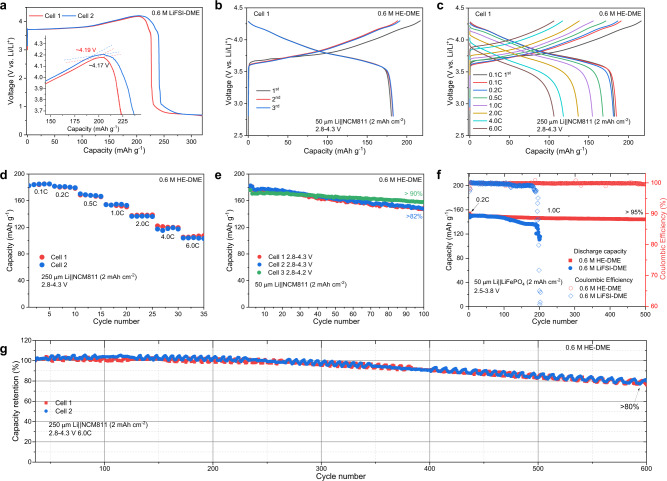
Electrochemical performance. **a**, **b** Galvanostatic charge/discharge curves of LiNi_0.8_Co_0.1_Mn_0.1_O_2_(NCM811) cells in **a** 0.6 M LiFSI-DME, **b** 0.6 M HE-DME electrolytes within the voltage range of 2.8–4.3 V at a rate of 0.1 C (1 C = 180 mA g^−1^). Cells were tested with a capacity of 2 mAh cm^−2^ for NCM811 and 50 μm lithium metal foils, resulting in a negative/positive capacity ratio (N/P) of 5. **c** Galvanostatic charge/discharge curves of NCM811 cells in the 0.6 M HE-DME electrolyte at different rates. **d** Electrochemical rate capability of NCM811 in the 0.6 M HE-DME electrolyte. **e** Cycling performance of NCM811 in the 0.6 M HE-DME electrolyte cycled at a 0.1 C rate for three cycles before cycling at a 0.333 C rate at different voltage ranges. **f** Cycling performance of LiFePO_4_ (LFP) cells cycled at a 0.2 C rate for three cycles before cycling at a 1.0 C rate. **g** Long-term cycling capacity retention of NCM811 cells in the 0.6 M HE-DME electrolyte cycled at a rate of 6.0 C.

The oxidation stability of the 0.6 M HE-DME electrolyte is investigated in Li||LiNi_0.8_Co_0.1_Mn_0.1_O_2_ (NCM811) cells (Supplementary Fig. 23) in the voltage range of 2.8–4.3 V vs Li/Li^+^ with a cathode areal capacity of 2.0 mAh cm^−2^. The upper cut-off voltage is challenging for the DME solvent because its relatively low oxidation stability. In combination with the 0.6 M LiFSI-DME electrolyte, Li||NCM811 cells are not able to reach the cut-off voltage of 4.3 V at a current density of 0.1 C (Fig. 2a), presumably because the cathode results in undesired oxidation of the electrolyte, catalyzed by the formed high-valence Ni species upon de-lithiation (charging)^23^. In comparison, the 0.6 M HE-DME electrolyte shows significantly improved reversible cycling when charged to 4.3 V, where two reproducible cells deliver similar charge/discharge profiles with a specific capacity of 182 mAh g^−1^(Fig. 2b and Supplementary Fig. 24). Li||NCM811 cells with 0.6 M LiDFOB-DME, 0.6 M LiTFSI-DME and 0.36 M LiNO_3_-DME electrolytes are also evaluated (Supplementary Figs. 25–28). Cells with 0.6 M LiDFOB-DME electrolyte can charge to 4.3 V, resulting in ~20 cycles, followed by a rapid decay to ~30% of the initial capacity after 50 cycles (Supplementary Figs. 25 and 28), similarly due to the continuous electrolyte and lithium consumption^24^ as observed in Li||Cu cells (Supplementary Fig. 6). The 0.36 M LiNO_3_-DME electrolyte does not support cycling at all (Supplementary Fig. 27). The above results indicate that the 0.6 M HE-DME electrolyte improves the oxidation stability of the NCM811 cathode significantly. The rate performance is evaluated in Li||NCM811 cells (Fig. 2c, d and Supplementary Fig. 29). When charged at 6.0 C (1080 mA g^−1^), more than 60% capacity retention is achieved (Fig. 2d) while the corresponding charge/discharge curves remain comparable, reflecting stable cycling. Cycling performance tests of the 0.6 M HE-DME electrolyte in Li||NCM811 cells are performed at 0.333 C (Fig. 2e, Supplementary Figs. 30 and 31), resulting in a capacity retention of over 82% after 100 cycles charged to 4.3 V. When the charged cut-off voltage is lowered to 4.2 V, an enhanced capacity retention of more than 90% is obtained, showing reduced reactions with DME. This is further demonstrated by the LiFePO_4_ cathodes cycled at 2.5–3.8 V, resulting in a capacity retention of more than 95% after 500 cycles, which implies that a more stable interphase is formed with the 0.6 M HE-DME electrolyte compared to the 0.6 M LiFSI-DME electrolyte in the suitable voltage range (Fig. 2f and Supplementary Fig. 32).

As an ultimate test of the power density and cycle life, long-term cycling of the 0.6 M HE-DME electrolyte at an aggressive rate of 6.0 C in Li||NCM811 cells was conducted (Fig. 2g, Supplementary Figs. 29 and 33). The capacity retentions of ~85% and 80% are achieved after 500 and 600 cycles, respectively. Increasing the cycling rate from 0.333 C to 6.0 C increases the CE from 99.3% to 99.8%, indicating that shorter exposure to the high potentials reduces the interfacial reactions of DME to some extent. After long-term cycling at 6.0 C for 1000 cycles, a capacity of more than 155.0 mAh g^−1^ can be recovered at a rate of 0.5 C (Supplementary Fig. 33). These results further support the DME is responsible for the capacity decay for high-voltage cathodes, but mixing of several salts in this HE electrolyte demonstrates substantial improvements and promising application potential.

### Lithium metal deposits morphology and microstructure

**Fig. 3:**
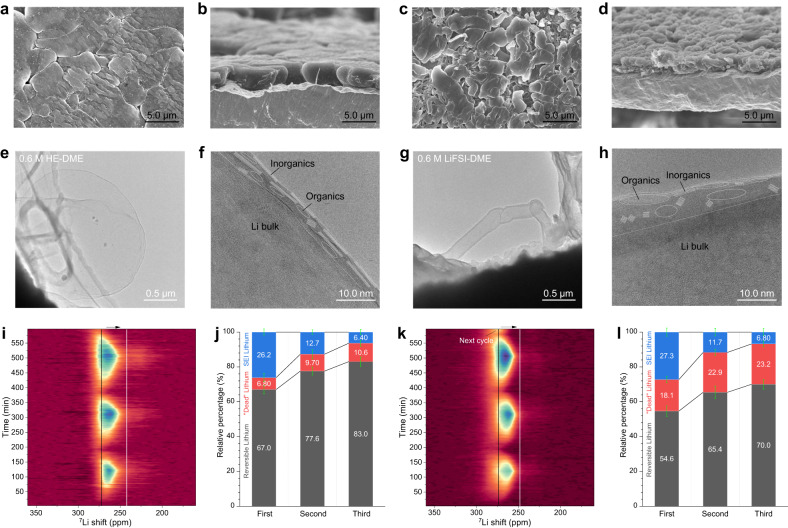
Morphology and microstructure of the lithium deposits. Lithium-metal morphology after plating **a**, **b** for the 0.6 M HE-DME electrolyte; **c**, **d** for the 0.6 M LiFSI-DME electrolyte. **a**, **c** Top view; **b** and **d** cross-sectional view. Microstructure of deposited lithium metal and interfacial phase from cryo-TEM images **e**, **f** for the 0.6 M HE-DME electrolyte; **g**, **h** for the 0.6 M LiFSI-DME electrolyte. The enlarged images of **f** and **h** are shown in the Supplementary Figs. 46 and 47. Operando ^7^Li solid-state NMR spectra and quantification of the lithium species, **i**, **j** for the 0.6 M HE-DME electrolyte; **k**, **l** for the 0.6 M LiFSI-DME electrolyte. Lithium species in the SEI (blue bars), reversible lithium metal (gray bars), and “dead” lithium metal residual species (red bars) are derived from the lithium metal integrated intensity ratio *I*(stripping)/*I*(plating) and the CE. Error bars are obtained by different tests. The corresponding galvanostatic charge/discharge curves of Cu||LiFePO_4_ cells at different electrolytes are shown in Supplementary Fig. 60.

To determine the origin of the improved electrochemical performance of HE-DME in combination with lithium metal anode, the morphology of lithium metal plated on bare Cu foil is investigated with scanning electron microscopy (SEM) (Fig. 3a–d and Supplementary Figs. 34–38). The deposited lithium metal in the 0.6 M HE-DME electrolyte is compact and well connected to the Cu substrate with particle sizes in the order of 10 µm (Fig. 3a, b and Supplementary Fig. 34). In contrast, for the 0.6 M LiFSI-DME electrolyte, porous and dendritic lithium deposits are observed that are less well connected to the Cu substrate (Fig. 3c, d and Supplementary Fig. 35) which can be held responsible for the residual dendritic lithium after stripping (Supplementary Fig. 36). For the 0.6 M HE-DME electrolyte, the Cu substrate after stripping shows less residual lithium metal (Supplementary Figs. 37, 38 and Supplementary Note 4). The lithium metal deposition process is further investigated with in-situ electrochemical atomic force microscopy (AFM) (Supplementary Figs. 39–41 and Supplementary Note 5), where the deposited lithium metal particles in the 0.6 M HE-DME electrolyte grow comparatively larger into a more compact morphology.

Cryo-transmission electron microscopy (cryo-TEM) is used to evaluate the lithium metal and SEI structure^25^ in both the 0.6 M HE-DME and LiFSI-DME electrolytes (Fig. 3e–h). In the 0.6 M HE-DME electrolyte, large lithium metal particles with thin SEI (~6 nm thick) are observed (Fig. 3e, f and Supplementary Figs. 42–44), which is different from the whisker and needle-like lithium metal deposits with thicker and non-uniform SEI layer in 0.6 M LiFSI-DME electrolyte (Fig. 3g, h and Supplementary Figs. 43 and 45). Being inorganic-dominant, this indicates that more anionic groups participate in the SEI formation for the 0.6 M HE-DME electrolyte (Fig. 3f and Supplementary Fig. 46). Whereas for the 0.6 M LiFSI-DME electrolyte, a mosaic structured SEI is formed, which is dominated by DME solvent decomposition (Fig. 3h and Supplementary Fig. 47). Selected area electron diffraction (SAED) and high-resolution TEM imaging (Supplementary Fig. 48) reveal that lithium-metal growth is different in these electrolytes. In the 0.6 HE-DME electrolyte, large spherical crystallites are observed, with the (110) planes parallel to Cu substrate (Supplementary Fig. 48a, c). This is consistent with a previous study which indicated that this crystalline texturing is beneficial to increase the homogeneity of lithium growth^26^. It signifies that after nucleation, lithium-ion transport facilitates the regular and homogeneous lithium-metal growth in the 0.6 HE-DME electrolyte. This does not appear to apply in the 0.6 M LiFSI-DME electrolyte where the particles are polycrystalline in nature (Supplementary Fig. 48b and d). Cryo-scanning transmission electron microscopy (STEM) electron energy loss spectroscopy (EELS) mapping also reveals a very different elemental distribution in the SEIs (Supplementary Fig. 49 and 50). In the 0.6 M HE-DME electrolyte, the outer surface is rich in O, while F, S, N and B are uniformly distributed over the surface of the particles (Supplementary Figs. 49 and 50). However, for the 0.6 M LiFSI-DME electrolyte, part of the outer surface is rich in C and O, and overall C is much more abundantly present. The SEI composition is further studied using X-ray photoelectron spectroscopy (XPS) (Supplementary Figs. 51–59 and Supplementary Note 6), where O 1s, F 1s, and N 1s spectra confirm that the inorganic Li-F, Li-N, B-F, Li-O, and B-O species dominate the SEI in the 0.6 M HE-DME electrolyte. The presence of these species could be responsible for a more facile and homogeneous lithium-ion supply, supporting dense lithium metal growth^27^, and less decomposition of solvent species in 0.6 M LiFSI-DME electrolyte.

Operando solid-state ^7^Li NMR is used to determine the origin of lithium loss during plating/stripping in both electrolytes^28^ (Supplementary Note 7). To enable quantification, an anode-less Cu||LiFePO_4_ full cell configuration is employed^29,30^. As expected, the ^7^Li metal resonance is absent before cycling and appears upon plating at ~260–270 ppm (Fig. 3i, k) reaching the highest integrated intensity after charging. Subsequently, the intensity decreases during discharging (lithium stripping), leaving some intensity, which represents the “dead” or inactive lithium metal. From the operando ^7^Li NMR spectra and the CE, the fraction of reversible lithium metal, dead lithium metal and lithium in the SEI is calculated for the first three cycles^31^ and shown in Fig. 3j, l. During the first cycle, both electrolytes have similar lithium loss into SEI formation, however, the “dead” lithium metal fraction is smaller in the 0.6 M HE-DME electrolyte, which contributes to the higher CE observed for this electrolyte (Fig. 3i, j). During the second and third cycles, the fraction of “dead” lithium metal grows in both electrolytes (Supplementary Fig. 61), being higher in the 0.6 M LiFSI-DME electrolyte, which results in a 2.4-times larger fraction of “dead” lithium metal. At the end of each plating process, the ^7^Li metal resonance in the 0.6 M HE-DME electrolyte moves to lower ppm values as compared to that in 0.6 M LiFSI-DME electrolyte. It reaches values close to that of lithium metal foil at ~246 ppm^30^, demonstrating that the 0.6 M HE-DME electrolyte results in a more dense lithium metal morphology^31,32^, consistent with SEM observations above.

### Morphology, microstructure, and composition of the cathode electrolyte interphase

**Fig. 4:**
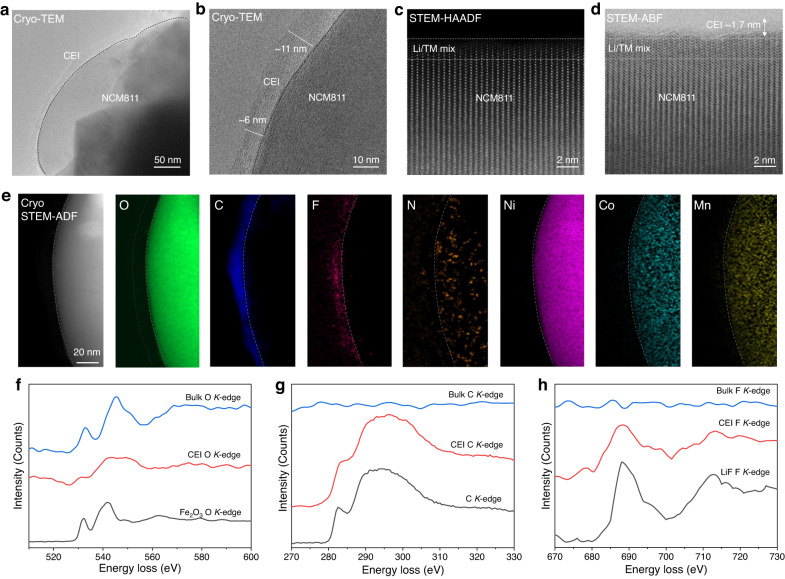
Morphology, microstructure and composition of the cathode electrolyte interphase (CEI). **a**, **b** Cryo-TEM images of the CEI after cycling in the 0.6 M HE-DME electrolyte. **c** Atomic-resolution high-angle annular dark field (HAADF) and **d** annular bright field (ABF)-scanning transmission electron microscopy (STEM) images collected at room temperature. A Li/TM (TM: transition metal) mixed layer was observed near the surface of the particle with a thickness of around 2 nm in both the HAADF and ABF images, in addition, a layer around 1.7 nm was found on the ABF image using its ability to detect light elements. **e** Cryo-STEM electron energy loss spectroscopy (EELS) mapping of the NCM811 CEI including a cryo-STEM ADF image, and O, C, F, N, Ni, Co and Mn elemental maps. Scale bar is 20 nm. EELS fine structure of **f**, O *K*-edge in the bulk and CEI with Fe_2_O_3_ included as a reference; **g** C *K*-edge in the bulk and CEI with carbon as a reference; **h** F *K*-edge in the bulk and CEI with LiF as a reference.

To understand the stable cycling of the NCM811 cathode in the 0.6 M HE-DME electrolyte, the morphology, microstructure, and composition of the CEI is investigated. After cycling, the morphology of the cathode particles, where the secondary particles consist of densely packed primary sub-micron sized particles, is preserved in the 0.6 M HE-DME electrolyte when comparing SEM images taken before and after cycling (Supplementary Fig. 62). Cryo-TEM at −170 °C is performed to study the nanostructure and chemical composition of the air-sensitive CEI formed on the surface of the particles. Compared to the pristine material (Supplementary Fig. 63) a conformal CEI layer is formed after cycling with a thickness in the range of 6–11 nm (Fig. 4a, b, and Supplementary Fig. 64). The corresponding fast Fourier transform (FFT) patterns of the near-surface region in the NCM811 particles indicates that the original layered structure is largely preserved (Supplementary Fig. 64). Atomic-resolution high-angle annular dark field (HAADF) and annular bright field (ABF)-STEM images are collected from the cycled NCM811 electrodes at room temperature. A mixed Li/TM (TM: transition metal) layer of ~2 nm is observed at the surface of the NCM811 particle, in both HAADF and ABF images (Fig. 4c, d), which indicates that the detrimental phase transition to the rock-salt phase, which is observed for carbonate electrolytes^33^, has not occurred up to 50 cycles in the 0.6 HE-DME electrolyte. Cryo-STEM EELS mappings are recorded to study the elemental distribution in the CEI layer and the near-surface structure of the cycled cathode (Fig. 4e and Supplementary Fig. 65). The results indicate the presence of O-, C-, F- and N- containing components in the conformal CEI layer, where O, C and F are the main components participating in the CEI formation and N uniformly distributes on the surface of particle (Fig. 4e and Supplementary Note 8).

The EELS fine structure at O *K*-edge, C *K*-edge, and F *K*-edge provides further insight in the CEI and bulk NCM811 composition (Fig. 4f–h). The O (2*p*)-TM (3*d*) hybridized peaks at around 533 and 544 eV in the cathode bulk are relatively high in energy compared to the referenced O *K*-edge of Fe_2_O_3_. O in the CEI layer shows a bonding that is similar to organic polymer carbonate compositions^34^ (Fig. 4f), which is most likely the result of DFOB^-^ decomposition^35^. With a peak around 290 eV in C *K*-edge EELS spectra, the formation of carbonate bonds in the CEI layer is further supported (Fig. 4g). Finally, F *K*-edge EELS indicates the presence of LiF in CEI layer (Fig. 4h). The XPS depth profiling analysis further confirmed these observations of the element distribution, showing that O and F are the dominant species in CEI layer formed in 0.6 M HE-DME electrolyte (Supplementary Figs. 66–73 and Supplementary Note 9). This compositional analysis indicates that the CEI that formed in 0.6 M HE-DME electrolyte consists of components that support a high stability, in combination with a high conductivity.

### Solvation structure in HE electrolyte

**Fig. 5:**
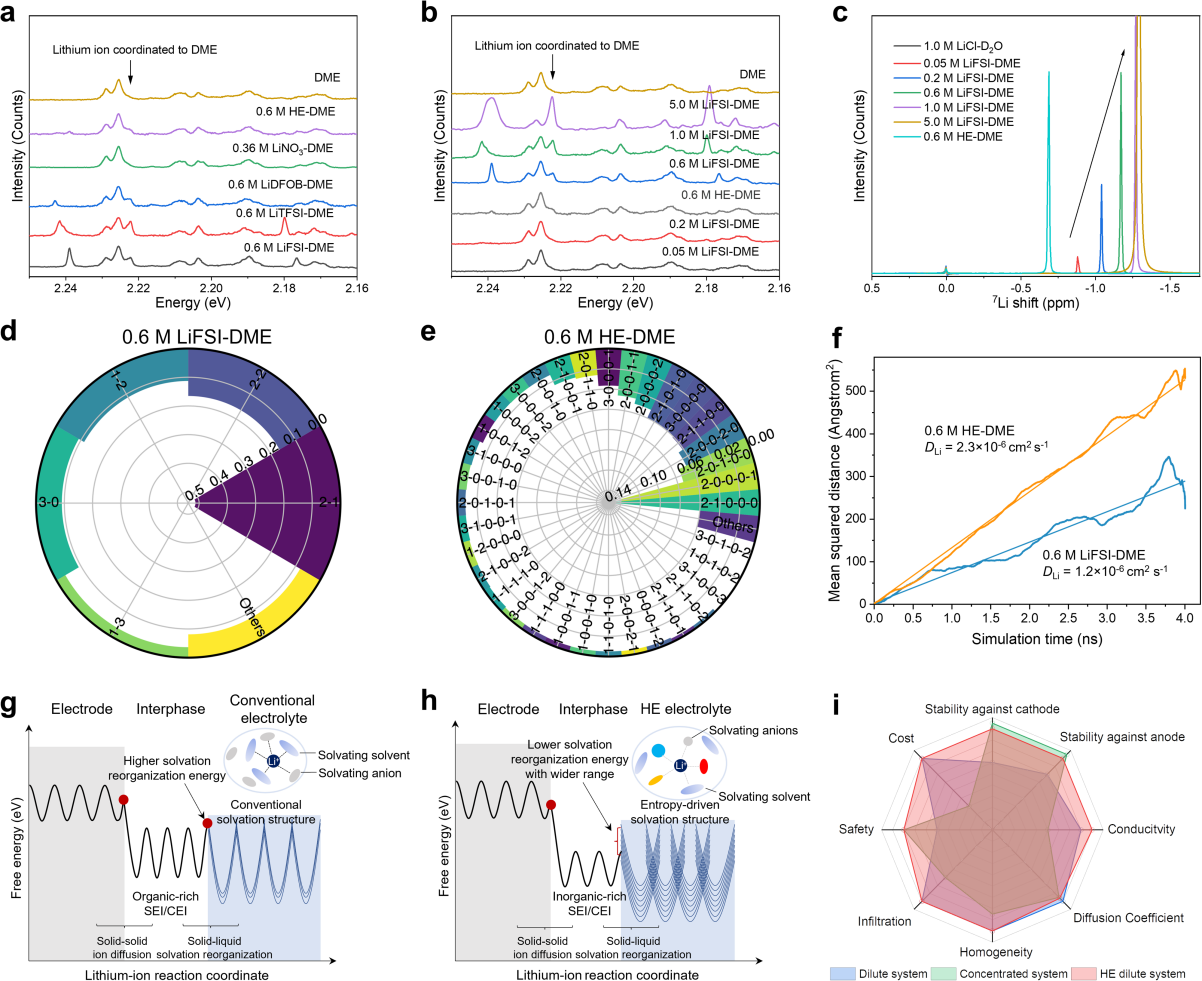
Solvation structure of HE electrolytes. **a** Raman spectra of the single salt and HE electrolytes. **b** Raman spectra of different LiFSI concentration DME electrolytes. **c** ^7^Li NMR spectra of the HE-DME electrolyte and the LiFSI in DME electrolytes, referenced to a 1 M LiCl in D_2_O solution. Lithium-ion coordination environments of **d** the 0.6 M LiFSI-DME electrolyte and of **e** the 0.6 M HE-DME electrolyte determined from MD simulations (detailed description in the Supplementary Tables 2 and 3). **f** The lithium-ion self-diffusion coefficient (*D*_Li_) from the MD simulated mean squared displacement for the 0.6 M LiFSI-DME electrolyte (blue line) and the 0.6 M HE-DME electrolyte (orange line). **g**, **h** Schematic diagram illustrating the ion transport between electrode and electrolyte in a **g** conventional electrolyte and **h** 0.6 M HE electrolyte. **i** Performance of the HE electrolyte compared with conventional dilute electrolytes and high-concentration electrolytes.

The solvation structure of the single-salt and HE-DME electrolytes is investigated with Raman spectroscopy (Fig. 5a and Supplementary Figs. 74–76). The HE-DME electrolyte shows a weaker solvation interaction between lithium ions and the DME solvent, indicated by the decreased peak intensity at ~2.22 eV^36,37^, compared with the single-salt electrolytes (Supplementary Fig. 76). In the 0.36 M LiNO_3_-DME electrolyte, this peak also appears weaker, which in this case should be attributed to the poor solubility of LiNO_3_ in DME^38^, where the strong Li^+^-NO_3_^−^ interaction results in a lower conductivity compared to the HE-DME electrolyte (Supplementary Table 1). In comparison with the different concentrations of the LiFSI-DME electrolyte (Fig. 5b and Supplementary Figs. 75 and 76), solvation in the 0.6 M HE-DME electrolyte is most similar to that of the dilute electrolytes (0.2 M and 0.05 M). This is also very different from the strong interactions between anions and lithium ions in the concentrated electrolytes (5.0 M)^39,40^. In line with this, the ^7^Li chemical shift of the 0.6 M HE-DME electrolyte indicates weaker shielding and therefore weak solvation; even weaker than a dilute 0.05 M LiFSI-DME electrolyte (Fig. 5c).

The HE electrolyte introduces a diversity in anion species, which in turn are expected to result in a larger variety of solvation structures, weakening the interaction between lithium ions and DME/anions as inferred above from NMR. To gain more insights into the solvation structure, density functional theory (DFT) and molecular dynamics (MD) simulations were carried out (Supplementary Fig. 77, Supplementary Tables 2 and 3). The various principal anion species in the 0.6 M HE-DME electrolyte result in a rich diversity of more than 30 types of lithium-ion solvation environments, much more than what is predicted for the 0.6 M LiFSI-DME electrolyte (Fig. 5d, e and Supplementary Figs. 78 and 79). The simulated self-diffusion coefficient of 2.3 × 10^−6^ cm^2^ s^−1^ is larger than that of the 0.6 M LiFSI-DME electrolyte (Fig. 5f), indicating improved lithium-ion mobility in agreement with the measured conductivity (Fig. 1f and Supplementary Table 1).

## Discussion

The MD simulations demonstrate that the introduction of several salts into the HE electrolyte leads to a much larger diversity in solvation structures, expressing the higher entropy, and to a higher lithium-ion mobility, as compared to single salt electrolytes with the same salt concentration. A way of defining the relationship between the entropy and the dynamic properties of liquids was introduced by Rosenfeld^14^ in 1977, which became known as excess entropy scaling, an approximate semi-quantitative relationship that is currently widely employed to estimate dynamic properties of liquids, such as diffusion constants and heat conductivities^15^. In general, excess entropy scaling indicates that the diffusivity increases with the entropy of the system. It can provide a qualitative argument for the present observation, where increasing the entropy by introducing multiple salts (resulting in a richer variation in solvation structures), has resulted in an increase of the diffusivity and conductivity, while keeping the total salt molarity the same. An intuitive explanation is that increasing the number of principle components in an electrolyte will lead to a wider distribution in diffusional barriers based on a wider diversity in solvation structures. Early studies have shown that in regular lattices, random distributions in energy barriers for diffusion typically enhances three-dimensional diffusion, as compared to a uniform energy barrier, which can simply be explained by the availability of a percolation network with lower-than-average energy barriers^41^.

The solvation structure of the liquid electrolyte plays a dominant role in the charge transfer between electrolyte and the electrode as well as in the SEI formation, where the resulting SEI morphology and composition determine the lithium-ion transport through the SEI. According to the radial distribution function (RDF) of the 0.6 M LiFSI-DME electrolyte obtained from the MD simulations (Supplementary Fig. 80), oxygen shows a strong tendency to coordinate with the lithium-ion in comparison to the other elements, indicating a relatively strong interaction between lithium ions and solvent molecules. However, in the HE-DME electrolyte, fluorine and nitrogen also coordinate with lithium ions, indicating more anion-rich solvation structures. This rationalizes the observation that for the HE electrolyte, both the SEI on anode and the CEI on the cathode are rich in decomposed salt anions. These salt anions, responsible for the higher electrochemical stability, facilitate lithium-ion transfer between the electrolyte and SEI/CEI^42,43^ and most likely also a higher lithium-ion conductivity in both the SEI and CEI. As for the SEI-electrolyte interface, the large diversity in solvation structures in the HE electrolyte leads to a wider range of solvation energies, as indicated by DFT (Supplementary Fig. 81). This diversity results in lower solvation reorganization energies that facilitate lithium-ion diffusion as well as charge transfer towards the interphase as schematically illustrated in Fig. 5g, h. The de-solvation processes in the entropy-dominated and conventional dilute electrolyte are further illustrated in Supplementary Fig. 82. In the HE electrolyte, the inorganic rich SEI/CEI and improved lithium-ion kinetics are attributed to the increasing entropy of mixing, resulting in denser lithium metal growth, despite the low concentration of HE electrolyte. Based on the above results, the characteristics of conventional dilute electrolytes, high salt concentration electrolytes and HE electrolytes are compared in Fig. 5i. From this comparison, the HE demonstrates promising assets, especially realizing that it enables improved stability against the anode/cathode in low salt concentration liquid electrolytes, which typically can be achieved in highly concentrated electrolytes (Supplementary Fig. 83).

In summary, we present liquid electrolytes with multiple salts and investigated the role and impact of entropy in this class of materials. Via introducing multiple salts (e.g., LiFSI, LiTFSI, LiDFOB, and LiNO_3_) in an ether solvent, a HE electrolyte has been prepared for the proof of concept, which exhibits a higher reversibility for lithium metal plating/stripping and a higher oxidation stability for Ni-rich cathode charging to a high cut-off voltage and results in improved rate performance. Despite the low salt concentration (0.6 M) and the poor oxidation stability of DME, the high entropy endows the solution with the demonstrated promising electrolyte properties. The results show that more salt anions participate in the solvation structures of the low-concentration HE electrolyte, resulting in a thinner and inorganic-rich SEI compared to the single salt electrolyte. The improved interphase properties enhance anodic and cathodic electrochemical stability (Supplementary Fig. 84), and results in more compact lithium metal plating and higher oxidative stability of ether-based solvents for 4 V-class lithium batteries. This rationalises the improved reversibility of the charge/discharge cycles and improved rate performance that exceeds these of conventional electrolytes. Moreover, the observed weaker solvation strengths between lithium ions and the solvents/anions, as compared to the commonly single-salt low- and high-concentration electrolytes, is held responsible for the enhanced charge transfer and the improved electrolyte conductivity. This is the consequence of the increased entropy of mixing leading to more diverse and more facile solvation rearrangements in the HE electrolyte (Supplementary Figs. 82 and 83).

The compositional landscape of HE materials is huge, as the chemistry and relative amounts of both solvents and salts can be varied. The present investigation suggests that raising the entropy by introducing multiple salts can be used to improve its functional properties through the solvation structure, where the choice of specific salts and solvents should be guided by their interactions with the specific anode and cathode chemistry. This is further supported by preparing a five-component salt HE electrolyte supporting even longer stable cycling (Supplementary Figs. 85–87 and Supplementary Note 10). This exploration motivates more fundamental and systematic research, which is of general scientific importance and will guide the development of better electrolyte systems and beyond.

## Methods

### Materials and synthesis

Lithium metal foils (thickness of 250 µm), Cu foils and Al foils were purchased from MTI Corporation, and lithium metal foils (50 µm) were purchased from China Energy Lithium Co., Ltd. All lithium metal foils were washed three times with dimethoxyethane (DME) solvent before use. Cu foils were immersed in diluted acetic acid for several minutes, subsequently washed by deionized water and acetone three times, separately, then they were quickly dried in the vacuum oven of glove box at room temperature. DME solvent was purchased from Sigma-Aldrich, which was dehydrated with a 4 Å molecular sieve (Sigma-Aldrich) to eliminate the possible trace water. Lithium bis(fluorosulfonyl)imide (LiFSI), lithium bis(trifluoromethanesulfonyl) imide (LiTFSI), Lithium Difluoro(oxalato)borate (LiDFOB) were obtained from Sigma-Aldrich, and Lithium bis(pentafluoroethanesulfonyl)imide (LiBETI) were obtained from 3 M company, which were dried under vacuum condition at 80 °C for 24 h after purchased. Lithium nitrate (LiNO_3_, > 99.9%) was purchased from Shenzhen Capchem Technology Co., Ltd and used as-received. All the electrolytes were prepared by dissolving the specific amount of different lithium salts in DME solvent in an Ar-filled glove box (H_2_O < 0.1 ppm, O_2_ < 0.1 ppm). 0.6 mol L^-1^ (M) LiFSI-DME, 0.6 M LiTFSI-DME, 0.6 M LiDFOB-DME and 0.36 M LiNO_3_-DME electrolytes denote that the corresponding concentration of different salts are dissolved in DME, where 0.6 M LiNO_3_-DME electrolyte can’t be prepared because of the relatively low salt solubility. HE-DME electrolyte was prepared by dissolving 0.15 M LiFSI, 0.15 M LiTFSI, 0.15 M LiDFOB and 0.15 M LiNO_3_ into DME solvent with the total concentration of lithium ion to be 0.6 M. 5-component 0.6 M HE-DME electrolyte was prepared by dissolving 0.15 M LiFSI, 0.10 M LiTFSI, 0.10 M LiBFTI, 0.10 M LiDFOB and 0.15 M LiNO_3_ into DME solvent with the total concentration of lithium to be 0.6 M.

LiFePO_4_ was obtained from Leneng Technology for which the cathodes were prepared by mixing LiFePO_4_ material, poly(vinylidene difluoride) (PVDF, MTI) binder and Super P (Alfa Aesar) conductive carbon in a weight ratio of 92:4:4. The resulting slurry was cast on the Al foil then dried at 60 °C for 6 h, followed by drying overnight at 120 °C in a vacuum oven. Li_4_Ti_5_O_12_ anode was purchased from MTI Corporation as received. LiNi_0.8_Co_0.1_Mn_0.1_O_2_ (NCM811) was synthesized using coprecipitation method. The certain amount of alkaline aqueous solution (NH_4_OH and NaOH) was poured into deionized water (1.5 L) to form the base solution in a tank reactor under continuous stirring. Then, a 2 M solution of NiSO_4_ ⋅ 6H_2_O, CoSO_4_ ⋅ 7H_2_O, and MnSO_4_ ⋅ H_2_O with a molar ratio of 8:1:1 and an aqueous solution of 5 M NH_4_OH and 10 M NaOH were added into the base solution in the tank reactor with a steady rate of 8 mL min^−1^. The coprecipitation temperature was controlled at 50 °C, and pH value was maintained at around 11 by NH_4_OH with stirring speed of 500 rpm under nitrogen atmosphere. The coprecipitated Ni_0.8_Co_0.1_Mn_0.1_(OH)_2_ precursor was prepared, which was subsequently washed by deionized water and ethanol for four times and dried in a vacuum at 120 °C for 24 h. The apparent and tap density of Ni_0.8_Co_0.1_Mn_0.1_(OH)_2_ precursor are 1.88 g cm^−3^ and 2.06 g cm^−3^, respectively. For preparation of NCM 811 materials, the as-obtained precursor was mixed with LiOH·H_2_O at a molar ratio of 1:1.03; then firstly heated at 500 °C for 5 h and subsequently calcined at 780 °C for 12 h in oxygen atmosphere. After cooling naturally, the obtained material was directly put into an Ar-filled glovebox to prevent any moisture exposition. The NCM811 electrodes were prepared by mixing active material, Super P and PVDF binder in the mass ratio of 90:5:5 in N-methyl-2-pyrrolidone (NMP) solvent and cast on Al foil and then dried at 60 °C for 6 h, followed by drying in a vacuum oven at 120 °C overnight. X-ray diffraction pattern demonstrates the pure phase of this prepared NCM811 material.

### Electrochemical measurements

Electrochemical cycling tests of all batteries were based on CR2032 coin cells assembled in an Ar-filled glove box (H_2_O < 0.1 ppm, O_2_ < 0.1 ppm) with Celgard 2500 separator and tested at room temperature, unless stated otherwise. 70 μL electrolytes were injected into each coin cell for comparison. All coin cells were tested using multi-channel battery testing systems (Land CT2001A or Lanhe G340A) at room temperature. Symmetric Li||Li cells were assembled to study the cycling stability under different current densities with various electrolytes. 15.6 mm diameter lithium metal foils with 250 μm thickness were used as both the working and counter electrodes. For LiǁCu cells, 14 mm diameter lithium metal foils were used as the reference, while 16 mm Cu foils was used as a working electrode with the effective area for lithium deposition of 1.54 cm^2^. During cycles, capacity of 1 mAh cm^−2^ lithium was deposited on Cu foils at a current density of 0.5 mA cm^−2^ and then stripped to a cut-off voltage of 1.0 V vs Li/Li^+^.

Electrochemical cycling performance of LiFePO_4_ and NCM811 electrodes (12 mm diameter) are all with an areal capacity of 2 mAh cm^−2^ tested with lithium metal foils with a thickness of 50 μm as counter electrode. Li||NMC811 cells were electrochemically cycled between 2.8 and 4.3 V under a 0.1 C rate for three cycles before cycling at 0.333C rate (1 C = 180 mA g^−1^). Li||LiFePO_4_ cells were cycled in the galvanostatic mode, whereas a voltage range of 2.5–5.0 V was used to gauge the oxidation stabilities of the different electrolytes for which LiFePO_4_ cathode doesn’t show the extra redox reaction from Fe^2+^ to Fe^3+^ above ~3.8 V. For electrochemical rate capabilities of Li||Li_4_Ti_5_O_12_ and Li||NMC811 cells, the areal capacity of 2 mAh cm^−2^ for Li_4_Ti_5_O_12_ (12 mm diameter) and capacity of 2 mAh cm^−2^ for NMC811 were used with lithium metal foils having a thickness of 250 μm as counter electrode. Cyclic voltammetry (CV) of LiǁCu cells with various electrolytes were conducted at a scan rate of 0.8 mV s^−1^ from −0.1 to 2.5 V vs. Li/Li^+^. Electrochemical impedance spectra (EIS) of the symmetric cells were collected on an Autolab (PGSTAT302N) in the frequency range of 0.1 Hz–1 MHz with a potential amplitude of 10 mV.

The lithium ion transference number (*t*_Li_^+^) of the electrolytes was measured via the method from Abraham et al.^7^. The polarization potential (Δ*V*) of 10 mV was used for symmetric Li||Li cells using the HE-DME electrolyte until the polarization currents reached a steady state The corresponding EIS measurements were collected before and after the polarization. The *t*_Li_^+^ was calculated using the following Eq. (1):

1 $${t}_{{{Li}}^{+}}=\frac{{I}^{{{{{{\rm{ss}}}}}}}{R}_{b}^{{{{{{\rm{ss}}}}}}}(\varDelta V-{I}^{0}{R}_{i}^{0})}{{I}^{0}{R}_{b}^{0}(\varDelta V-{I}^{{{{{{\rm{ss}}}}}}}{R}_{i}^{{{{{{\rm{ss}}}}}}})}$$

where Δ*V* is the applied potential, *I*^0^ is the initial current and *I*^ss^ is the steady-state current; *R*_b_^0^ and *R*_b_^ss^ are the initial and steady-state values of the bulk resistances and *R*_i_^0^ and *R*_i_^ss^ are initial and steady-state values of the interfacial resistances, respectively, which were examined by impedance measurements before and after the potentiostatic polarization.

Ionic conductivity of electrolytes was measured using symmetric stainless steel||stainless steel cells by collecting the electrochemical impedance (*R*) at room temperature, and calculated using the Eq. (2):

2 $$\sigma=\frac{L}{R\times S}$$

where *σ* is ionic conductivity, *S* is the effective area of electrode, *L* stands for the thickness between two stainless-steel electrodes, respectively. Test cells were assembled with a Polytetrafluoroethylene (PTFE) ring between two electrodes. Hence, the effective area of electrode is calculated based on the inner diameter PTFE ring, and the thickness two stainless-steel electrodes is based on total thickness of PTFE ring.

For the evaluation of Al foil corrosion, Al||Li cells were assembled with a 250 µm thick lithium metal foil with 70 μL of different kinds of electrolytes. The cells were tested with the potentiostatic mode at 4.2 V vs. Li/Li^+^ for 24 h.

### Materials characterization

Morphologies of electrodes were measured on a cold field scanning electron microscope (SEM, HITACH-S4800, SU8010). Elemental composition on the surface of the electrodes was analyzed by X-ray photoelectron spectroscopy (XPS, PHI 5000 VersaProbe II) using a monochromatic Al Kα X-ray source with X-ray settings being 100 µm 25 W 15 kV. Peaks were fitted using MultiPak software calibrated with respect to carbon (284.8 eV). The above morphology and composition characterization were performed with cells being disassembled after specific cycles in an Ar-filled glove box and rinsed with pure DME solvent three times to remove residual electrolyte, followed by drying in a glove box for several hours at room temperature to remove the residual solvent. Then these electrodes were transferred into the vacuum transfer boxes for measurements to avoid air exposure. Raman spectroscopy was measured by Micro-laser confocal Raman spectrometer (Horiba LabRAM HR800 spectrometer) equipped with an Olympus BX microscope and an argon ion laser (532 nm) at room temperature. All the electrolytes were hermetically sealed in quartz cuvettes in a glovebox before measurement. Powder X-ray diffraction (XRD) was performed using a Bruker D8 Advance diffractometer equipped with a Cu Kα radiation source (λ_1_ = 1.54060 Å, λ_2_ = 1.54439 Å at 40 kV and 40 mA) and a LynxEye_XE detector.

### Cryo-transmission electron microscopy (cryo-TEM) characterization

Conventional and cryo-(S)TEM experiments were performed on a scanning transmission electron microscope (STEM) (JEM-ARM300F, JEOL Ltd.) operated at 300 kV with a cold field emission gun and double Cs correctors. During image acquisition, the corresponding electron dose flux (units of number of electrons per square angström per second, e^−^ Å^−2^ s^−1^) was recorded. Conventional STEM images were taken with a dose rate of over 1000 e^−^ Å^−2^ s^−1^ with an exposure time for each image of several seconds. Cryo-TEM images were obtained with an exposure time for each image of around 0.3 s with built-in drift correction function using the OneView and K2 cameras. Cryo-TEM images were taken with an electron dose rate of 50–500 e^−^ Å^−2^ s^−1^. Short-exposure single-frame shots were used to estimate the defocus and make it as close as possible to Scherzer defocus. EELS spectra were acquired on a GIF Quantum camera with a dispersion of 1 eV/channel, utilizing the Dual EELS capability to correct for drift in the low-loss centered on the zero-loss peak and core-loss centered on the *C* K-edge. The EELS spectrum images were carried out with a camera length of 20 mm, and a pixel dwell time of 10 ms. Energy drift during spectrum imaging was corrected by centering the zero-loss peak to 0 eV at each pixel. Elemental maps were computed through a two-window method in a pre-edge window fitted to a power-law background and a post-edge window of 50–200 eV on the core-loss signal. Analysis of the spectra has been performed in Gatan microscopy suite software. For cryo-TEM sample preparation and transfer, cells were disassembled immediately in an argon-filled glovebox after cycling and then both lithium metal anodes and NCM811 cathodes were rinsed with pure DME three times to remove lithium salts, followed by drying in the glove box for one hour at room temperature to remove the residual solvent. During the washing procedure, ~10 mL DME was carefully dropped onto each of the electrodes one time to reduce additional artifacts on the electrodes.

For cryo-TEM preparation of lithium metal anode, a lacey carbon TEM grid was put on Cu foil working electrode and assembled into LiǁCu cells in an argon-filled glovebox. The cells were discharged at a constant current density of 1.0 mA cm^−2^ for 15 min, after which the TEM grid was taken out by disassembling the cells for measurement. The TEM grid was carefully transferred into the cryo-TEM holder in glovebox with a specialized shutter to prevent air exposure and ice condensation onto the sample introducing any side reactions. Once the cryo-TEM holder was transferred into TEM column, the temperature was maintained at around −170 °C using liquid nitrogen. For cryo-TEM preparation of NCM811 cathodes, conventional LiǁNCM811 cells were cycled at 0.333 C for 50 cycles in the voltage range of 2.8–4.3 V and then disassembled in glovebox. After rinsing the cathode, a small piece of cathode was sealed in an airtight container with pure DME inside. Then the sealed airtight container was taken out from glovebox and the sample was dispersed for three minutes by ultrasonic method. After that, the dispersed cathode was dropped on the TEM grids in glovebox and loaded into the cryo-TEM holder for further measurement. The same specialized shutter was also used to prevent air exposure. All cryo-TEM images are taken at around −170 °C to reduce beam damage. For the conventional STEM experiments, the above dispersed sample was dropped on a copper grid, dried for three hours in a vacuum and loaded into the double-tilt holder. The STEM-HAADF and ABF images were recorded at room temperature.

### In situ atomic force microscopy (AFM) characterization

In-situ electrochemical AFM measurement (Bruker Corporation) was performed with a three-electrode cell powered by an electrochemical workstation (CHI760E) in an argon-filled glove box (H_2_O < 0.1 ppm, O_2_ < 0.1 ppm). Cells were assembled with Cu substrate as working electrode and lithium metal stripe as the counter and reference electrodes. During the electrochemical measurement, cells were discharged at a constant current density of 0.5 mA cm^−2^ in which the images of lithium plating process were collected at different times. AFM topography images were collected with the peak force tapping mode and the ScanAsyst-Fluid tips (*k* = 0.7 N m^−1^) were used for their superior force control with a pN-level force between tip and electrode, diminishing the damage to sample surface in the liquid condition. The obtained AFM images were analyzed with NanoScope Analysis software.

### Liquid nuclear magnetic resonance (NMR) characterization

Liquid NMR spectra were recorded with an Agilent 400 MHz DD2 NMR spectrometer with 5 mm ONE NMR Probe at room temperature, which worked at 155.5 MHz on ^7^Li, 100.6 MHz on ^13^C, and 376.49 MHz on ^19^F, respectively. The chemical shift values are given in ppm. ^7^Li chemical shift was referenced to the standard solution: 1 M LiCl in D_2_O for ^7^Li (0 ppm). The external standard solutions were sealed into WILMAD coaxial insert tubes, and inserted into the 5-mm KONTES tubes with electrolytes and sealed with PTFE caps. Mestrelab Research Mnova software was used for data processing.

### Solid-state NMR characterization

Operando solid-state NMR measurements were conducted on a wide-bore Bruker Ascend 500 system equipped with a NEO console in a magnetic field strength of 11.7 T and a ^7^Li resonance frequency being 194.37 MHz using a solenoidal Ag-coated Cu coil. Operando static ^7^Li NMR measurements were performed using an automatic-tuning-and-matching probe (ATM VT X operando WB NMR probe, NMR Service) at room temperature which can allow for an automatic recalibration of the NMR radio-frequency (rf) circuit during an operando electrochemistry experiment. A highly shielded wire with low-pass filters was attached to the probe for electrochemical measurement, which could minimize the interferences between NMR and the electrochemistry circuit. Single-pulse with a π/2 pulse of 4 μs and recycle delay of 1.0 s was applied to acquire the 1D static spectrums. A recycle delay of three times of *T*_1_ was used each time, where *T*_1_ was determined using saturation recovery experiments. The electrochemical cell was simultaneously controlled by a Maccor battery testing system. A plastic capsule cell made out of polyether ether ketone (PEEK) was used for the operando NMR experiments. The cells were assembled using LiFePO_4_ cathode (areal capacity is 2.0 mAh cm^−2^) and Cu foils as working and counter electrodes with both a piece of Celgard and a piece of Glass fiber (Whatman GF/A) as separator. Before measurements, the assembled cells were rested for 2 h in glovebox. The operando capsule cell was aligned in an Ag-coated Cu coil with LiFePO_4_ and Cu foil electrode oriented perpendicular to *B*_0_ and parallel with respect to the *B*_1_ rf-field. During the static ^7^Li NMR measurements, the cells were charged to the capacity of 1 mAh cm^−2^ at current density of 0.5 mA cm^−2^. A charge cut-off capacity of 1 mAh cm^−2^ was used for lithium metal plating on Cu foils and a discharge cut-off voltage of 2.0 V for stripping. During charge-discharge process, NMR spectra were continuously acquired. The chemical shift of ^7^Li was referenced to 1 M aqueous solution of LiCl at 0 ppm. The spectra were processed in the Bruker Topspin software, using the automatic phase and baseline correction. Mestrelab Research Mnova software was used for data processing and analysis.

### Molecular dynamics (MD) simulations

MD simulations were conducted on single and HE electrolyte systems with different lithium salts and concentrations using the LAMMPS package^44^. Molecules and ions were described by the optimized potentials for a liquid simulations all-atom (OPLS-AA) force field^45^. Partial charges were computed by fitting the molecular electrostatic potential at the atomic centers with the Møller–Plesset second-order perturbation method with the correlation-consistent polarized valence cc-pVTZ(-f) basis set^46^. In order to create a certain concentration of salt within DME, a 1:10 salt/solvent ratio is required. Simulation boxes with dimensions of 60 × 60 × 60 Å were randomly packed with 1200 molecules of DME and 120 salt molecules using the software Packmol^47^. A cut-off distance of 1.1 nm was chosen for the Lennard-Jones interactions. A conjugate-gradient energy minimization was first performed on both simulation boxes. A time-step of 0.5 fs was chosen for the MD simulations performed after this point. Isothermal-isobaric ensemble simulations at 300 K was first performed for 5 ns in order to obtain the correct volumes of both systems. Subsequently, both systems were equilibrated at room temperature using canonical ensemble simulations for another 6 ns. The canonical ensemble simulations were continued for another 10 ns, and snapshots of the simulation were obtained every 0.5 ps. The solvation structures of the simulation were analyzed using the Python Materials Genomics (pymatgen) package^48^. The radial density functions and the diffusivities of the lithium ions were computed using the MDAnalysis package^49^.

### Density functional theory (DFT) calculations

Quantum chemical calculations were conducted using DFT method with Becke’s three parameters (B3) exchange functional in Lee-Yang-Parr (LYP) nonlocal correlation functional (B3LYP)^50,51^. All the geometry optimizations were performed with the B3LYP/6-31 + G(d,p) level. The energy calculations were performed at B3LYP/6-311 + +G(3df,3dp) level for more accurate calculation. All DFT calculations were performed by using the Gaussian 09 program package^52^. The solvation structure formation energy was calculated as following Eq. (3):

3 $${E}_{{{{{\rm{Form}}}}}}={E}_{{{{{\rm{cluster}}}}}}-\sum {E}_{{{{{\rm{molecule}}}}}}$$

where *E*_cluster_ is the energy of the solvation structure and *E*_molecule_ is the energy summation of all molecules forming the solvation structure.

## Supplementary information

Supplementary Information

## Data Availability

The data that support the findings within this paper are available from the corresponding author on request.
