# Supplementary material for: High entropy liquid electrolytes for lithium batteries
Source: Nat Commun. 2023 Jan 27;14:440. doi: 10.1038/s41467-023-36075-1 (PMC9918526; doi:10.1038/s41467-023-36075-1)
Supplement: Supplementary file 1 — Supplementary Information [file 41467_2023_36075_MOESM1_ESM.docx]

Supplementary information

**High entropy liquid electrolytes for lithium batteries**

Qidi Wang^1,8^, Chenglong Zhao^1,8^*, Jianlin Wang^2,8^, Zhenpeng Yao^3,4,8^, Shuwei Wang^5^, Sai Govind Hari Kumar^6^, Swapna Ganapathy^1^, Stephen Eustace^7^, Xuedong Bai^2^, Baohua Li^5^, Marnix Wagemaker^1^*

^1^Department of Radiation Science and Technology, Delft University of Technology, Delft, 2629JB, Netherlands.

^2^State Key Laboratory for Surface Physics, Institute of Physics, Chinese Academy of Sciences, Beijing, 100190, China.

^3^The State Key Laboratory of Metal Matrix Composites, School of Materials Science and Engineering, Shanghai Jiao Tong University, Shanghai, 200240, China.

^4^Center of Hydrogen Science, Shanghai Jiao Tong University, Shanghai, 200240, China.

^5^Shenzhen Key Laboratory on Power Battery Safety and Shenzhen Geim Graphene Center, School of Shenzhen International Graduate School, Tsinghua University, Guangdong, 518055, China.

^6^Department of Chemistry and Computer Science, University of Toronto, Toronto, Ontario, M5S 3H6, Canada.

^7^Department of Biotechnology, Delft University of Technology, Delft, 2629HZ, Netherlands.

^8^These authors contributed equally: Qidi Wang, Chenglong Zhao, Jianlin Wang, Zhenpeng Yao

*Corresponding authors: c.zhao-1@tudelft.nl; [m.wagemaker@tudelft.nl](mailto:m.wagemaker@tudelft.nl).

**Supplementary Notes**

**Supplementary Note 1.** To study the effect of HE electrolytes, several criteria have been taken into consideration while selecting the principal components. DME is selected as solvent because it strongly dissociates from various alkali metal salts, due to its high donor number. The salt concentration is chosen to be 0.6 M for two main reasons. First, because this is commercially compatible (higher concentrations increase the cost of batteries considerably, Supplementary Fig. 1). Second, because it offers a more challenging condition for the stability of the electrode/electrolyte interface. The selection criteria of the salts are their solubility in the DME solvent, commercial availability and the presence of oxygen containing anionic groups, aiming to balance the interaction between lithium-ions and DME. Based on these criteria, LiFSI, LiTFSI, LiDFOB and LiNO_3_ were selected to prepare the 0.6 M HE electrolyte, resulting in a uniform and stable solution.

**Supplementary Note 2.** The oxidation stability of single salt electrolytes is evaluated using Li||LiFePO_4_ cells at a low current density of 0.02C (1C=150 mAh g^-1^). A charge cut-off voltage of 5.0 V is set to study the oxidation stability of the electrolytes, making use of the absence of a redox reaction of LiFePO_4_ above ~3.8 V (Supplementary Fig. 4). This results in the onset of oxidation at ~4.81, 4.62, 4.36, and 4.19 V vs Li/Li^+^ for 0.6 M LiDFOB-DME, 0.6 M LiTFSI-DME 0.6 M LiFSI-DME and 0.36 M LiNO_3_-DME respectively. The reduction stability of single salt electrolytes is evaluated in Li||Cu cells (Supplementary Fig. 5-8), where the 0.6 M LiFSI-DME electrolyte shows the most reversible cycling with a Coulombic efficiency (CE) > 97.0% after 50 cycles. The other single salt electrolytes result in a CE < 50.0% after 50 cycles, and 0.36 M LiNO_3_-DME is not able to cycle at all showing large overpotentials of approximately ~ 400 mV, and large stripping capacities due to electrolyte decomposition (Supplementary Fig. 8). These results are qualitatively summarized in Fig. 1c, where 0.6 M LiFSI-DME is concluded to show the best overall stability in single salt electrolytes, and is selected as the control group in the detailed study of the 0.6 M HE-DME electrolyte. The different combination of salts in the electrolyte are also tested (Supplementary Fig. 9). From the comparisons it is clear that the HE electrolyte stands out in its performance.

**Supplementary Note 3.** Symmetric Li||Li cells are used to test the cycling stability and overpotential in both electrolytes, where the 0.6 M HE-DME electrolyte exhibits overpotentials of ~18 mV, ~10 mV and ~8 mV after the 1^st^, 50^th^ and 200^th^ cycle respectively at a current density of 1 mA cm^−2^, outperforming the single salt electrolytes (Supplementary Fig. 11 and 12), corresponding to a lower electrochemical impedance (Supplementary Fig. 12). Furthermore, the cycling stability at current densities of 0.5, 1.0, 3.0 and 5.0 mA cm^−2^ are evaluated, demonstrating the competitive conductivity of this 0.6 M HE-DME electrolyte (Supplementary Fig. 13). In contrast, the 0.6 M LiFSI-DME electrolyte is not able to cycle at high current densities in symmetric Li||Li cells. This can be attributed to its limited conductivity and SEI layer that results in concentration polarization at the surface of the electrodes^1^. The 0.6 M HE-DME electrolyte has a slightly higher reduction potential for the decomposition of anionic groups at ~1.32 V, and the current density is relatively small according to the cyclic voltammetry (CV) measurements of the Li||Cu cells (Supplementary Fig. 14-19). After the initial cycles, the reduction peak disappears (Supplementary Fig. 15), indicating that an SEI has been formed during the initial cycles^2^.

**Supplementary Note 4.** The denser and better connected lithium metal deposits formed in the 0.6 M HE-DME electrolyte is beneficial for electron transport, allowing efficient lithium metal stripping and suppressing the formation of “dead”/inactive lithium metal^3^. At the same time it minimizes the exposed surface area resulting in less electrolyte decomposition, suppressing the formation of porous and dendritic lithium on subsequent lithium metal deposition^4^.

**Supplementary Note 5.** The process of lithium metal deposition on the Cu current collector is also investigated with in-situ electrochemical atomic force microscopy (AFM), where in contrast to a regular cell, there is no pressure exerted by the separator on the lithium metal. At the early stage of plating of 36 s (0.005 mAh cm^-2^), for both electrolytes, the Cu electrodes are covered by lithium nanoparticles, the density of which appears slightly higher in the 0.6 M HE-DME electrolyte (Supplementary Fig. 39 and 40). After plating for 72 s (0.01 mAh cm^-2^), a distinct difference in plating emerges between the two electrolytes as larger particle sizes are observed in the 0.6 M HE-DME electrolyte. For the 0.6 M LiFSI-DME electrolyte, even more nanoparticles are observed as compared to 36 s of deposition, which continues up to at least 144s (0.02 mAh cm^-2^) (Supplementary Fig. 39). From 216 to 1080 s, part of these nanoparticles gradually grows in size resulting in a porous morphology. In comparison, the deposited Li-metal particles in 0.6 M HE-DME electrolyte grow larger into a more compact morphology (Supplementary Fig. 40). At 2520 s (0.35 mAh cm^-2^) the difference in particle size becomes even larger, being in the range of 10-500 nm in the 0.6 M LiFSI-DME electrolyte and in the range of 2-5 µm in the 0.6 M HE-DME electrolyte (Supplementary Fig. 39-41). The continuous nucleation of Li-metal deposits may be related to the much higher overpotential during lithium metal deposition in the 0.6 M LFSI-DME electrolyte which can be related to the lower conductivity of the electrolyte and the SEI^5,6^.

**Supplementary Note 6.** The SEI composition is further studied by X-ray photoelectron spectroscopy (XPS) (Supplementary Fig. 51-59). Based on the survey spectra (Supplementary Fig. 51), F, C and O elements dominate the SEI formed in the 0.6 M HE-DME electrolyte, and much less F is present in the SEI formed in the 0.6 M LiFSI-DME electrolyte, even with different sputtering depths. With increasing sputtering depth, looking deeper in the SEI formed in the 0.6 M HE-DME electrolyte, the F and O content increases and the C content decreases, indicating the initial decomposition products on the Cu substrate result from the anionic groups in the 0.6 M HE-DME electrolyte. At the largest sputtering depth, B appears originating from the DFOB^-^ anion (Supplementary Fig. 59), consistent with its high reduction potential (Supplementary Fig. 14). In addition, high-resolution O 1s, F 1s, and N 1s spectra confirm that the inorganic Li-F, Li-N, B-F, Li-O and B-O species dominate the SEI in the 0.6 M HE-DME electrolyte. In the 0.6 M LiFSI-DME electrolyte, the similar amount of S content before and after sputtering indicates continuous decomposition of the FSI^-^ group. These observations indicate that in comparison to the 0.6 M LiFSI-DME electrolyte, the 0.6 M HE-DME electrolyte results in larger, crystalline lithium metal particles, with a thinner and inorganic rich layered SEI. The latter can be held responsible for more facile and homogeneous Li-ion supply, supporting dense lithium metal growth, and less decomposition of electrolyte species, both of which explain the better reversibility of the lithium metal anode in combination with the 0.6 M HE-DME electrolyte.

**Supplementary Note 7.** Via the Knight shift, lithium metal can be distinguished from the diamagnetic lithium species in the SEI, and the amount can be quantified under the condition that the deposits are significantly smaller than the skin depth, the penetration depth, of the radiofrequency field (11 µm for the present measurements)^7^. As a result, these measurements allow the quantification of the amount of “dead” lithium metal after stripping, representing lithium metal deposits that are electronically disconnected from the Cu current collector.^7^ Combining this with the CE from the electrochemical measurement, the amount of lithium in the SEI can be quantified^8^. Additionally, operando solid-state ^7^Li NMR provides information on the evolution of the lithium metal morphology based on the characteristic chemical shifts of mossy structures (at ~260 ppm) and dendritic lithium (at ~270-280 ppm).^9-11^ In this work, lithium corrosion is not taken into account based on the relatively low corrosion current compared to the plating/stripping current^8^.

**Supplementary Note 8**. The CEI in the 0.6 M HE-DME electrolyte is rich in O, with a lower concentration as compared to the bulk cathode material, and in contrast the TM elements (Ni, Co and Mn) are only observed in the bulk cathode material indicating the CEI inhibits dissolution of TM ions. Generally, TM ion dissolution plays an important role in these systems, where DME oxidation generates acidic species that degrade the surface structure of the cathode^12^. The STEM-ABF image further demonstrates that TM ions do not participate in the formation of the CEI where a layer around 1.7 nm is only found in the ABF image (Fig. 4d) due to its ability to observe light elements, and not in the HAADF image which is sensitive to the heavier elements. This is thinner than observed with cryo-TEM, most likely because of the air and beam exposure of the CEI component.

**Supplementary Note 9****.** To further investigate the elemental distribution on the surface of the NMC811 (after cycling), XPS depth profiling analysis is carried out after cycling in the 0.6 M HE-DME electrolyte (Supplementary Fig. 66-73). The survey spectra (Supplementary Fig. 66) and atomic ratio’s (Supplementary Fig. 67) show that C, O and F are the dominant species in the CEI layer, consistent with the cryo-STEM EELS mapping results. The C 1*s*, O 1*s*, N 1*s* and S 2*p* spectra (Supplementary Fig. 68-71), indicate the presence of C–O, C=O, –CF_x_, N–O, S–N and –SO_x_, suggesting that the various anionic groups of the HE-DME electrolyte are involved in the formation of the CEI layer. Based on the F 1*s* and Li 1*s* spectra, the CEI formed in the 0.6 M HE-DME electrolyte is rich in LiF, the concentration of which increases with the depth in the CEI (Supplementary Fig. 72 and 73), which can be held responsible for the high-voltage stability of the CEI layer^13,14^.

**Supplementary Note 10****.** Based on the favorable, entropy-driven properties of the studied four-component 0.6 M HE electrolyte, a five-component 0.6 M HE electrolyte is prepared to further increase the entropy aims to improve functional properties. Lithium bis(pentafluoroethanesulfonyl)imide (LiBETI) is introduced as the fifth component to replace parts of the LiTFSI and LiDFOB salts. It was selected because of its larger complexity, further contributing to a larger entropy of mixing and additionally because of its higher oxidation stability^15^. This results in an electrolyte composition of 0.15 M LiFSI, 0.1 M LiTFSI, 0.1 M LiDFOB, 0.1 M LiBETI and 0.15 M LiNO_3_ with DME as the solvent, which is referred to as five-component 0.6M HE electrolyte. The stability towards lithium metal is investigated using Li||Cu cells (Supplementary Fig. 85a and 85b), demonstrating in direct comparison with the four-component 0.6M HE electrolyte a higher reversibility with CE exceeds 99.0%, which is related to the even more compact lithium metal deposition (Supplementary Fig. 85c). Furthermore, the oxidation stability of the five-component 0.6 M HE electrolyte is also improved, up to 4.61 V, after which the oxidation quickly passivates (Supplementary Fig. 85d). On comparing the 4 and 5 component HE electrolytes in rate capability and cycling performance tests in Li||NCM811 cells, it is found that the latter has a slightly higher capacity retention when charged/discharged at 6.0C (Supplementary Fig. 85e and 86) as well as a slightly better capacity retention at a charge/discharge rate of 0.333C (Supplementary Fig. 85f and 87). Thereby, a second example is provided of a multi component salt electrolyte, where we propose that the wider diversity in solvation structures leads to weaker DME solvation, finally resulting in an inorganic rich and more stable SEI as well as in a higher lithium-ion conductivity, responsible for its improved performance.

**Supplementary Figures**

**Supplementary Fig. 1. Illustration of electrolyte properties vs. lithium-salt concentration vs. electrochemical stability in liquid electrolytes.** Three parts of dilute, intermediate and concentrated solutions are shown, where in general electrochemical stability against anode/cathode are dominated by solvents for dilute systems and dominated by salts for concentrated systems.


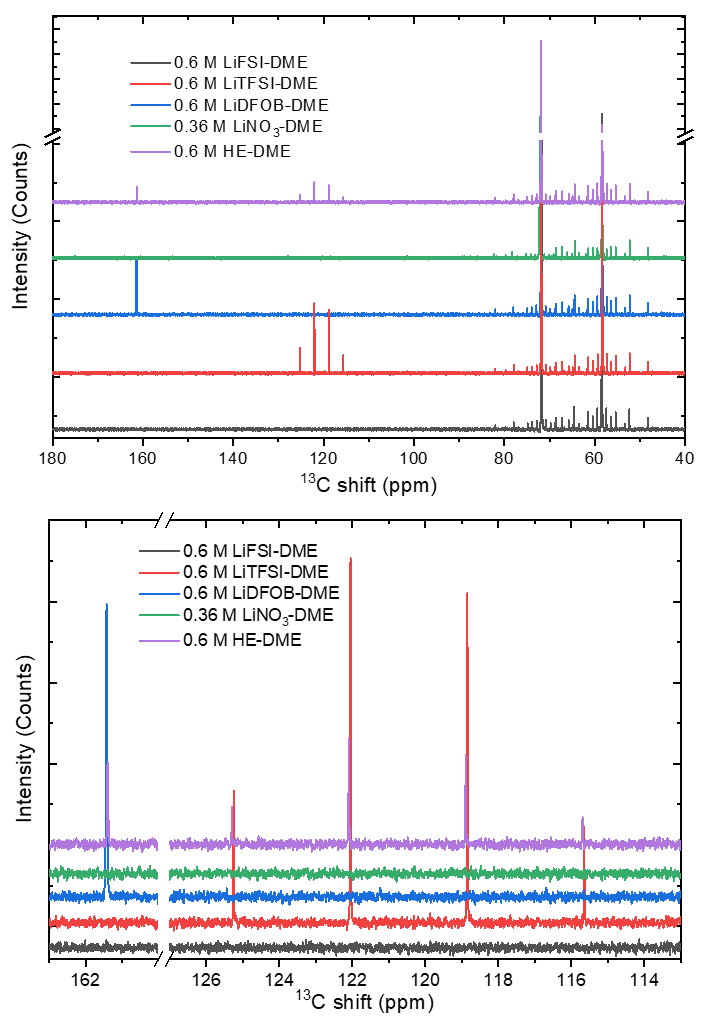


**Supplementary Fig. 2. Liquid** **^13^C nuclear magnetic resonance (NMR) spectra of single salt electrolytes and the as-prepared HE-DME electrolyte**. The top figure is the complete ^13^C chemical shift range; and the bottom shows sections of the spectra corresponding to the salt part. Compared with the ^13^C peaks at around 115.7, 118.9, 122 and 125.5 ppm assigned to TFSI^-^ group, these peaks showed an upfield shift in HE-DME electrolyte, but compared with ^13^C peak at around 161.5 ppm assigned to the DFOB^-^ group, the peak moved downfield in the HE-DME electrolyte.


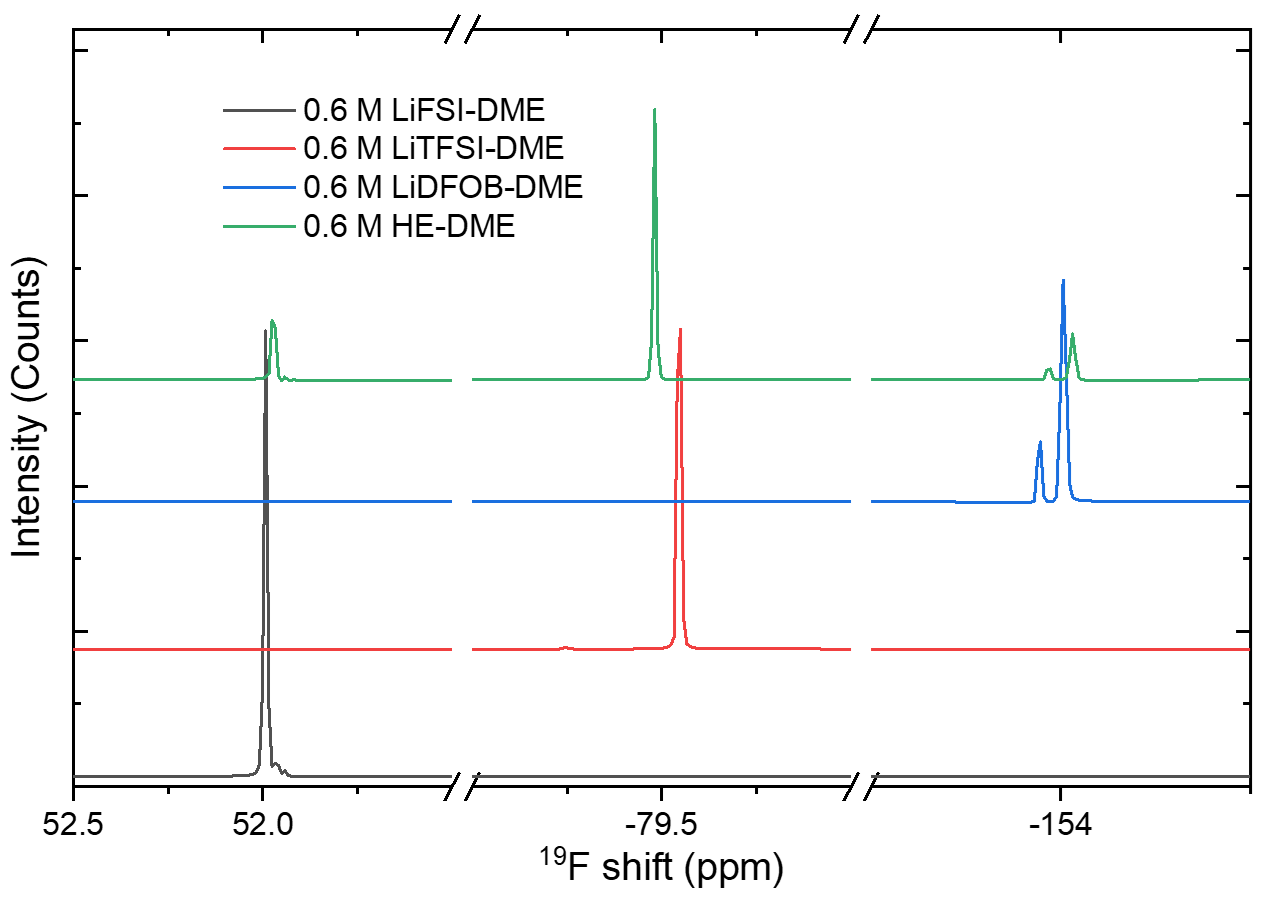


**Supplementary Fig. 3. Liquid** **^19^F NMR spectra of single salt electrolytes and the as-prepared HE-DME electrolyte**. The ^19^F NMR spectra in the HE-DME electrolyte include three parts, the peak around 52.0 ppm from FSI^-^ group, the peak around -80.0 ppm from TFSI^-^ group and the peak around -154.0 ppm from DFOB^-^ group. Compared with the peak in single salt electrolytes, the peaks at around 52.0 ppm and -154.0 ppm move upfield, yet the peak around -80.0 ppm moves downfield, indicating that different solvation structures of salt anion groups exist in the HE-DME electrolyte.


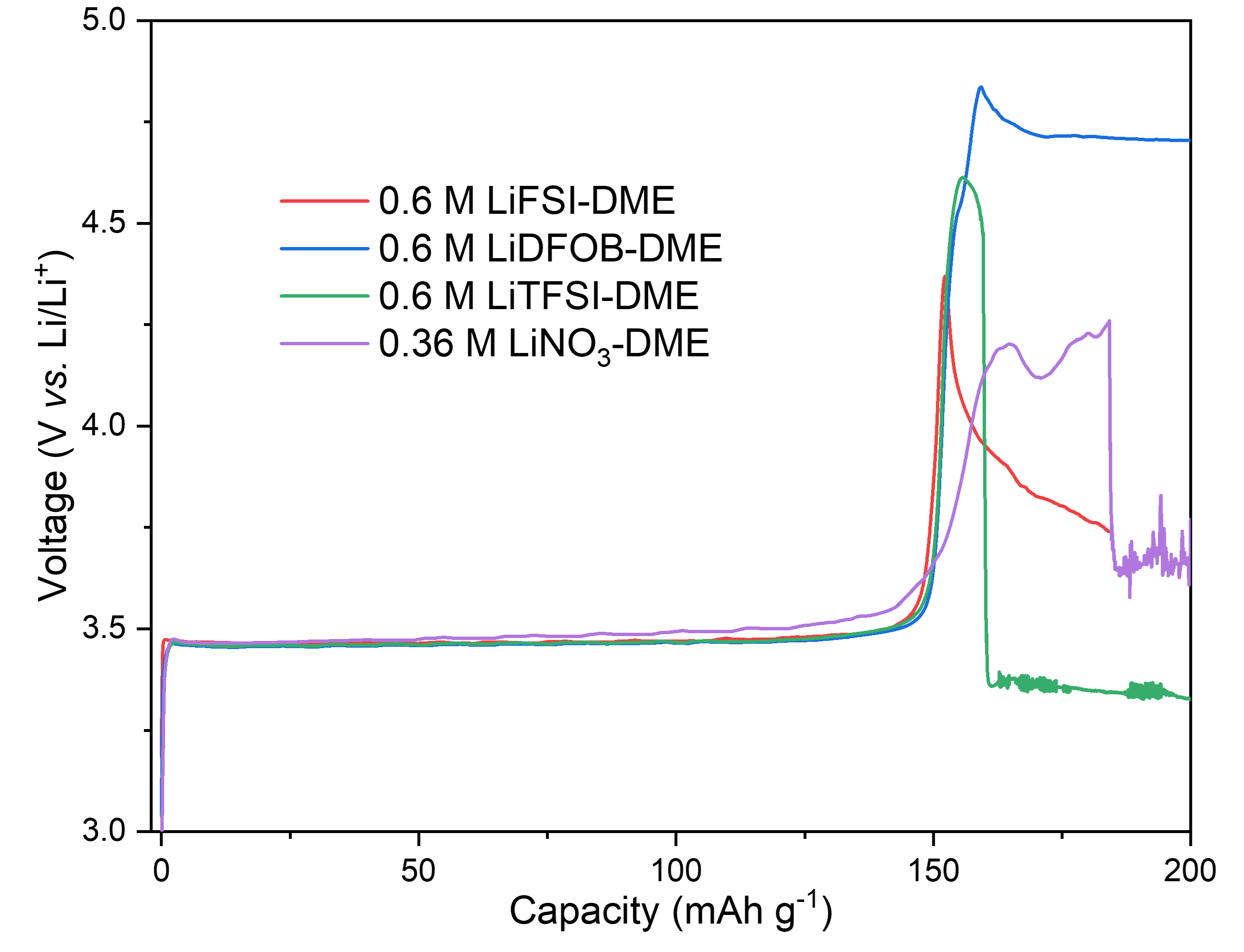


**Supplementary Fig. 4. The oxidation and reduction stability of the single salt electrolytes.** Galvanostatic charge profiles of Li||LiFePO_4_ cells with different electrolytes at a current density of 0.02C (1C=150 mAh g^-1^). A charge cut-off voltage of 5.0 V was set to study the oxidation stability of electrolytes. LiFePO_4_ cathode was chosen as it does not show the extra redox reaction from Fe^2+^ to Fe^3+^ on being charged to above ~3.8 V.


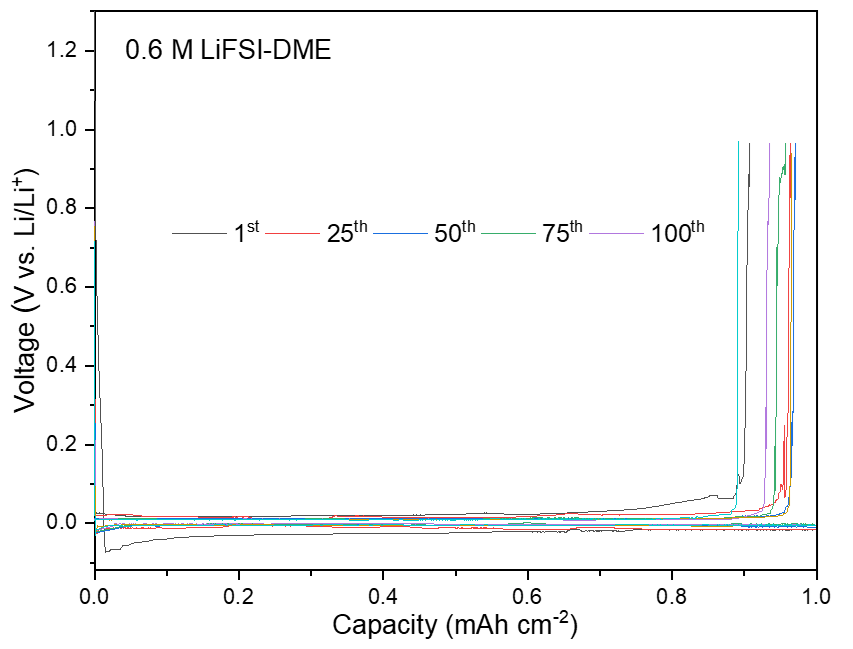


**Supplementary Fig. 5. Galvanostatic lithium plating/stripping profiles of Li||Cu cell with 0.6 M LiFSI-DME electrolyte.** The cells were plated for 2 h at 0.5 mA cm^−2^ followed by stripping to a cut-off voltage of 1.0 V vs. Li/Li^+^.


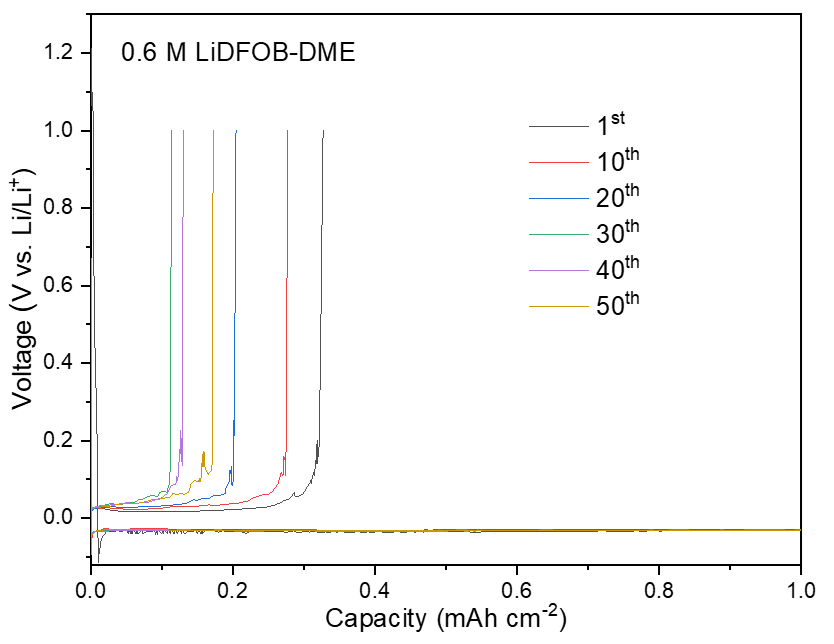


**Supplementary Fig. 6. Galvanostatic lithium plating/stripping profiles of Li||Cu cell with 0.6 M LiDFOB-DME electrolyte.** The cells were plated for 2 h at 0.5 mA cm^−2^ followed by stripping to a cut-off voltage of 1.0 V vs. Li/Li^+^.


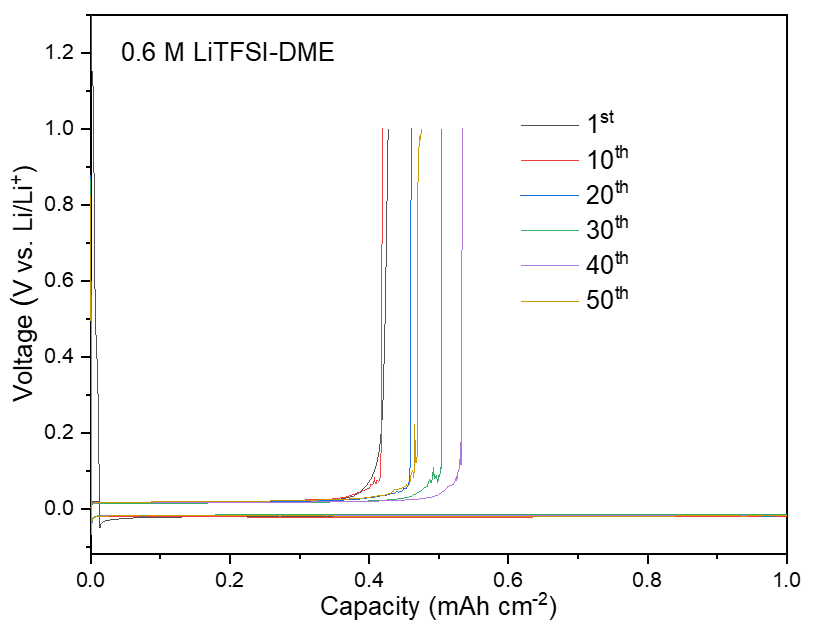


**Supplementary Fig. 7. Galvanostatic lithium plating/stripping profiles of Li||Cu cell with 0.6 M LiTFSI-DME electrolyte.** The cells were plated for 2 h at 0.5 mA cm^−2^ followed by stripping to a cut-off voltage of 1.0 V vs. Li/Li^+^.


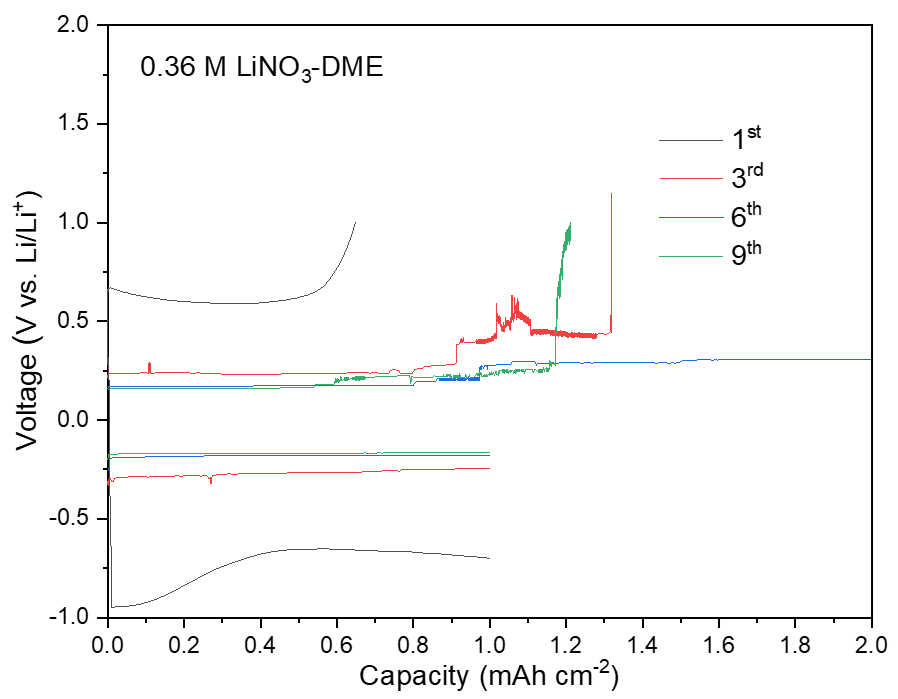


**Supplementary Fig. 8. Galvanostatic lithium plating/stripping profiles of Li||Cu cell with 0.36 M LiNO_3_-DME electrolyte.** The cells were plated for 2 h at 0.5 mA cm^−2^ followed by stripping to a cut-off voltage of 1.0 V vs. Li/Li^+^.

**
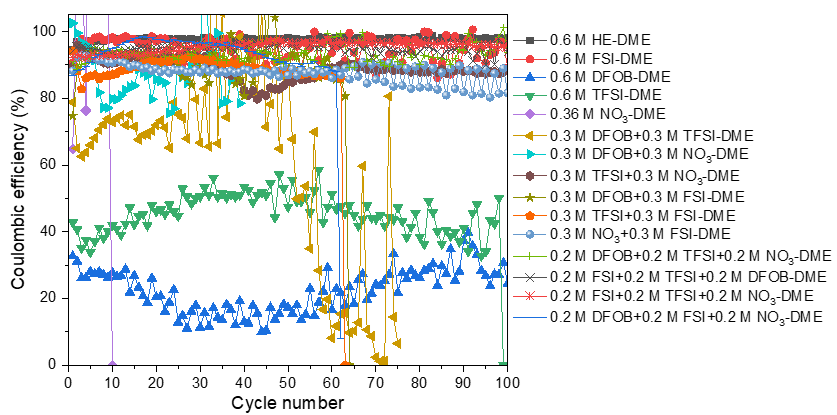
**

**Supplementary Fig. 9. Lithium plating/stripping Coulombic efficiency (CE) in Li||Cu cells using various electrolytes.** Lithium was electrodeposited at 0.5 mA cm^−2^ to a total capacity of 1 mAh cm^−2^.

**Supplementary Fig. 10. Potentiostatic charge profiles of as-prepared 0.6 M HE-DME electrolyte and 0.6 M LiFSI-DME electrolytes.** Li||Al cells were used to study the corrosion current of Al foil in different electrolytes at the polarization potential of 4.2 V vs. Li/Li^+^ for 24 h. In the 0.6 M LiFSI-DME electrolyte, the anodic current dramatically increased after a short time of about 2 h, suggesting a rapid dissolution of Al at high potential, whereas the HE-DME electrolyte showed a stable anodic current under the same condition.


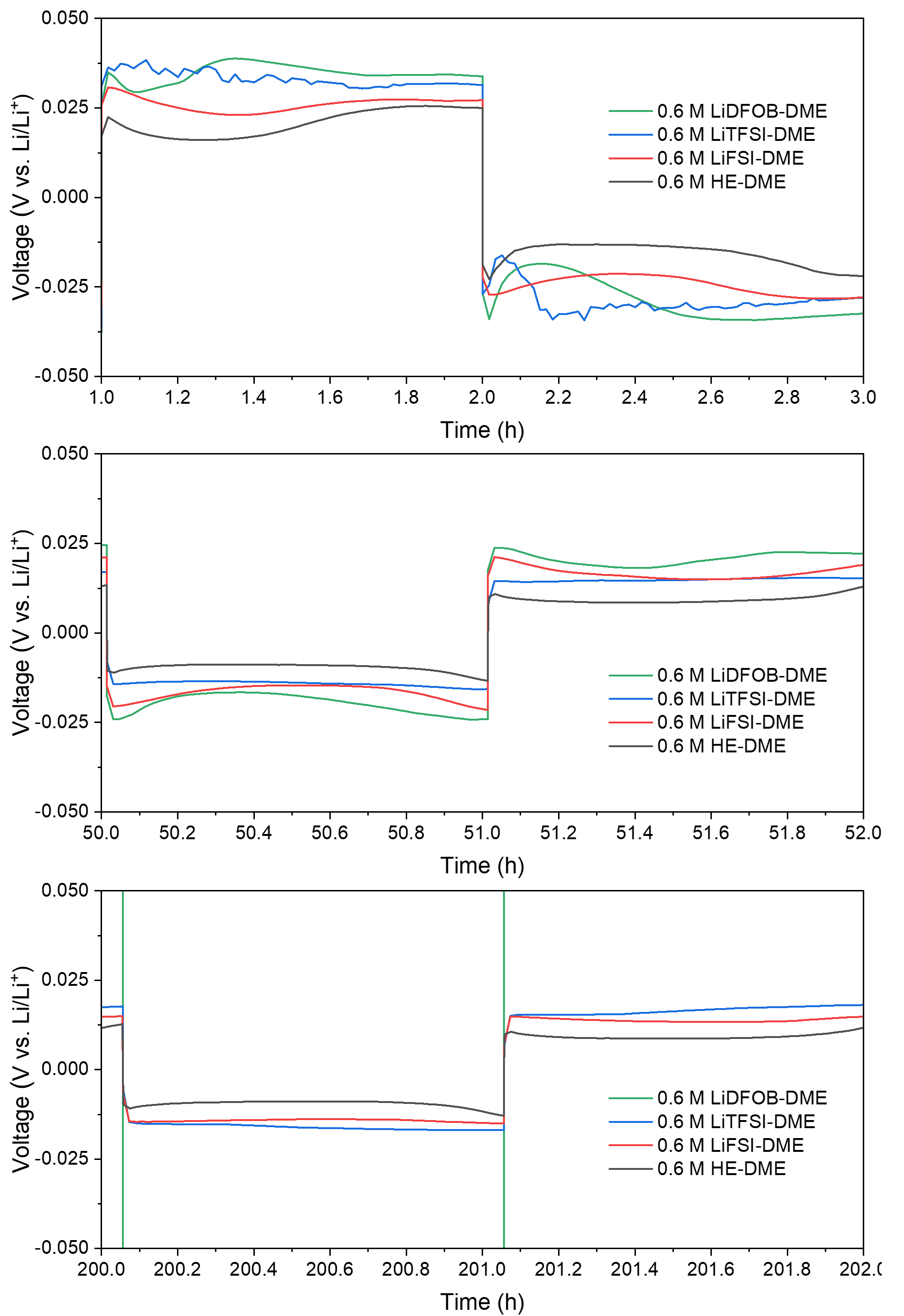


**Supplementary Fig. 11. Enlarged voltage profiles of symmetric Li||Li cells cycled in different electrolytes.** The LiǁLi symmetric were cycled under a current density of 1 mA cm^-2^ with each plating/stripping time of 1 h.


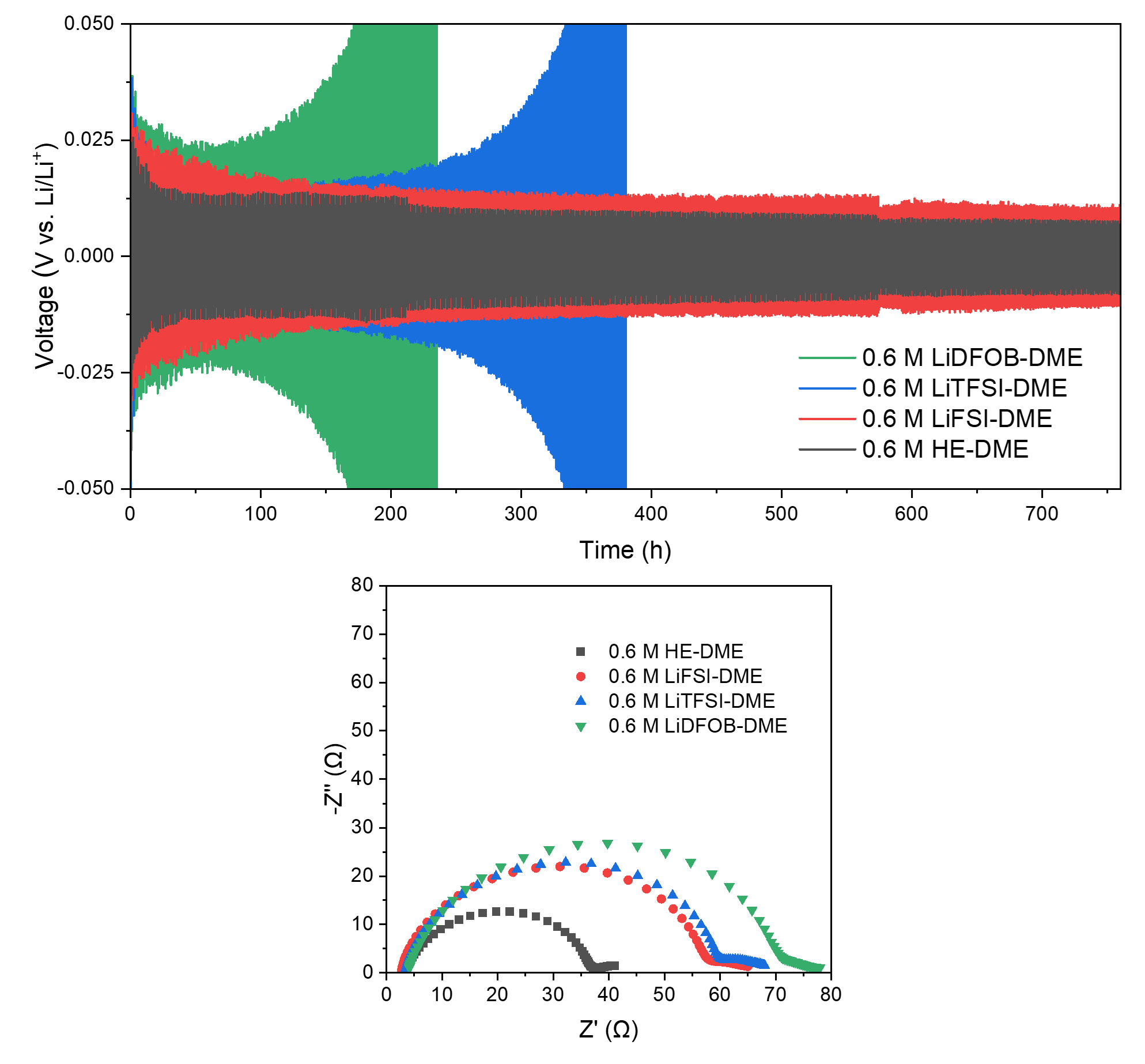


**Supplementary Fig. 12. Evolution of voltage profiles of symmetric Li||Li cells and electrochemical impedance spectra (EIS) in different electrolytes.** The LiǁLi symmetric cells were cycled under a current density of 1 mA cm^-2^ with each plating/stripping time of 1 h.


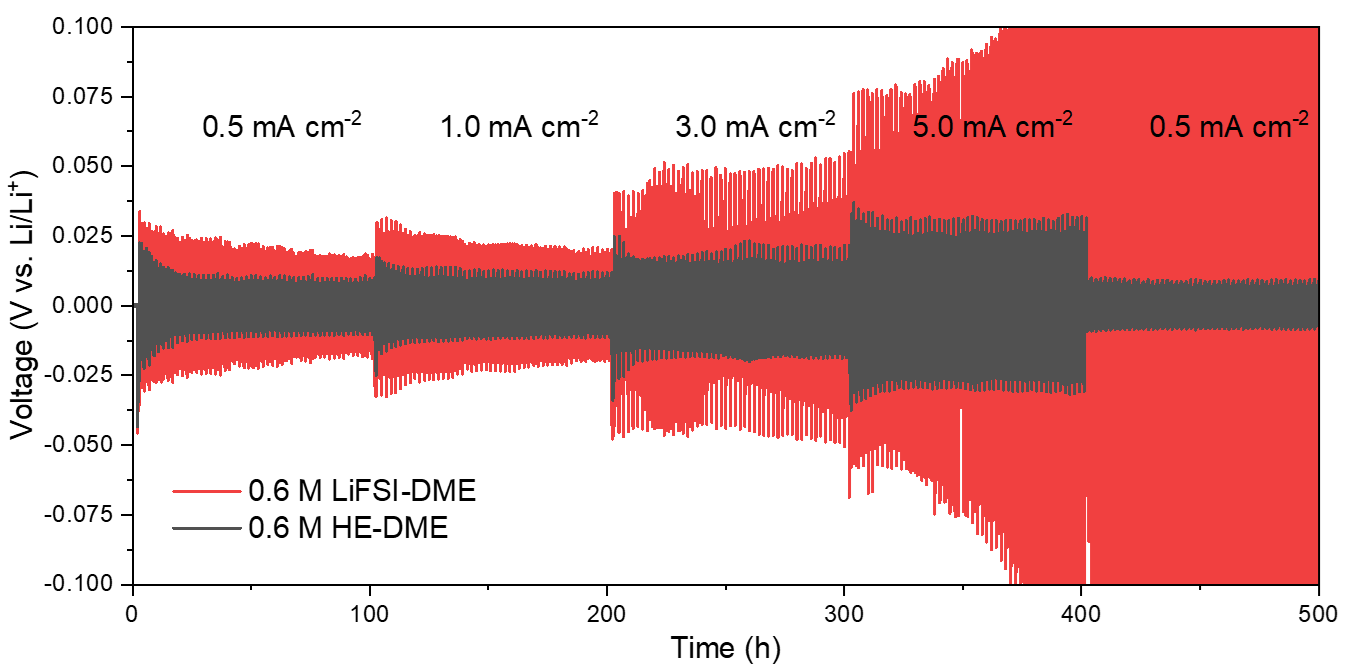


**Supplementary Fig. 13. Rate profile for symmetric cells with different electrolytes.** Li||Li cells at current density from 0.5 to 5 mA cm^-2^ with each plating/stripping time of 1 h.


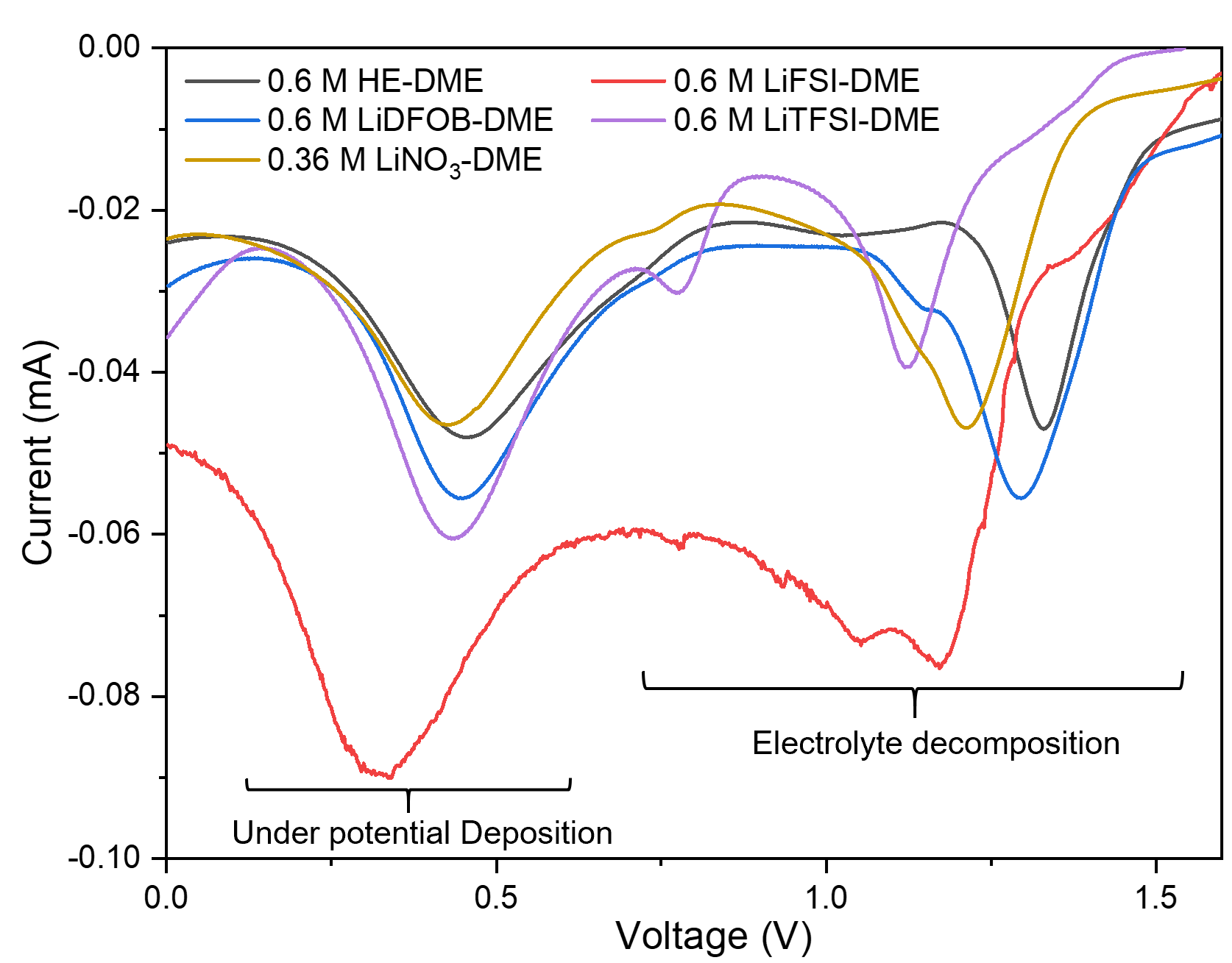


**Supplementary Fig. 14.** **Cyclic voltammetry (CV) curves of Li||Cu cells.** Cells were tested at a scan rate of 0.8 mV s^-1^ from -0.1 to 2.5 V vs. Li/Li^+^.


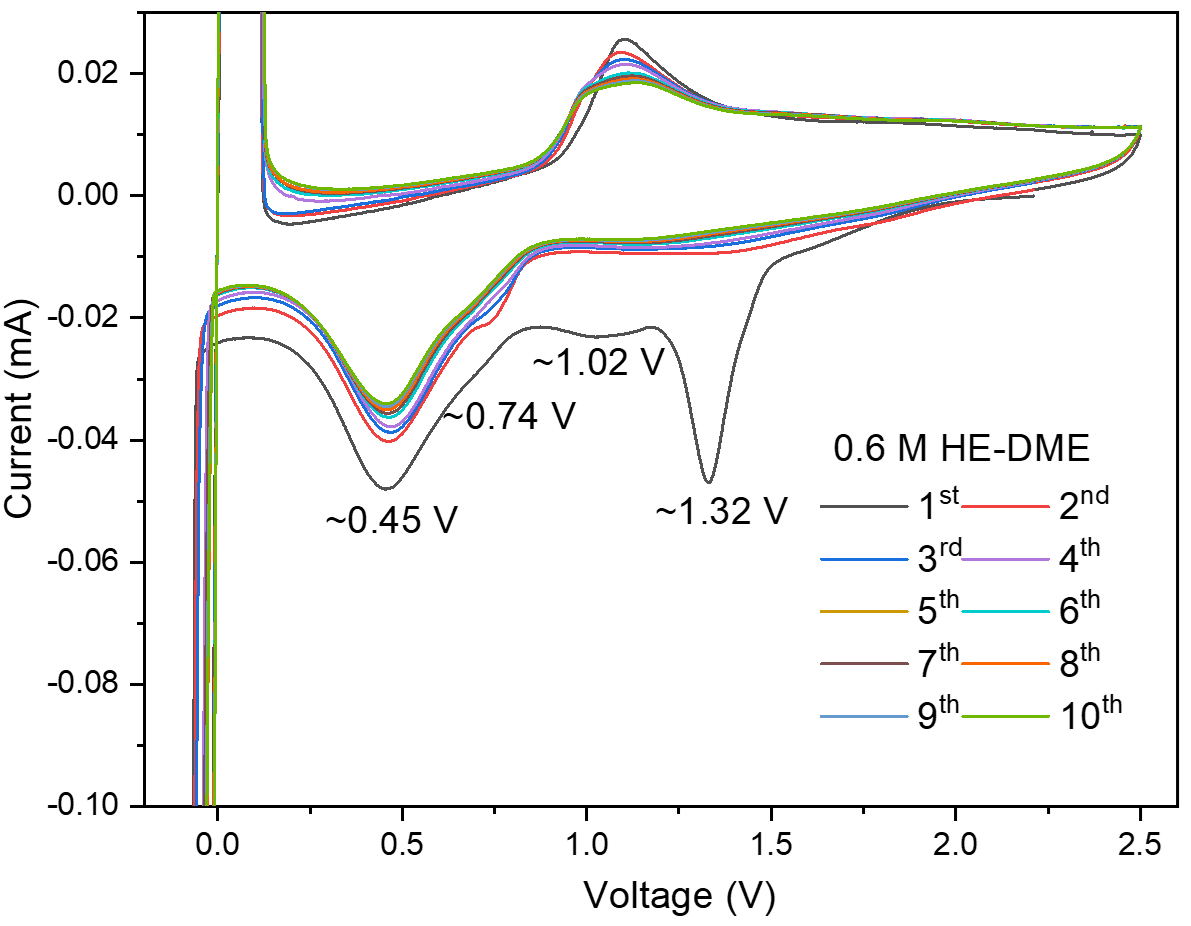


**Supplementary Fig. 15. CV curves of Li||Cu cell in 0.6 M HE-DME electrolyte.** A scan rate of 0.8 mV s^-1^ was applied with a voltage range from -0.1 to 2.5 V vs. Li/Li^+^.


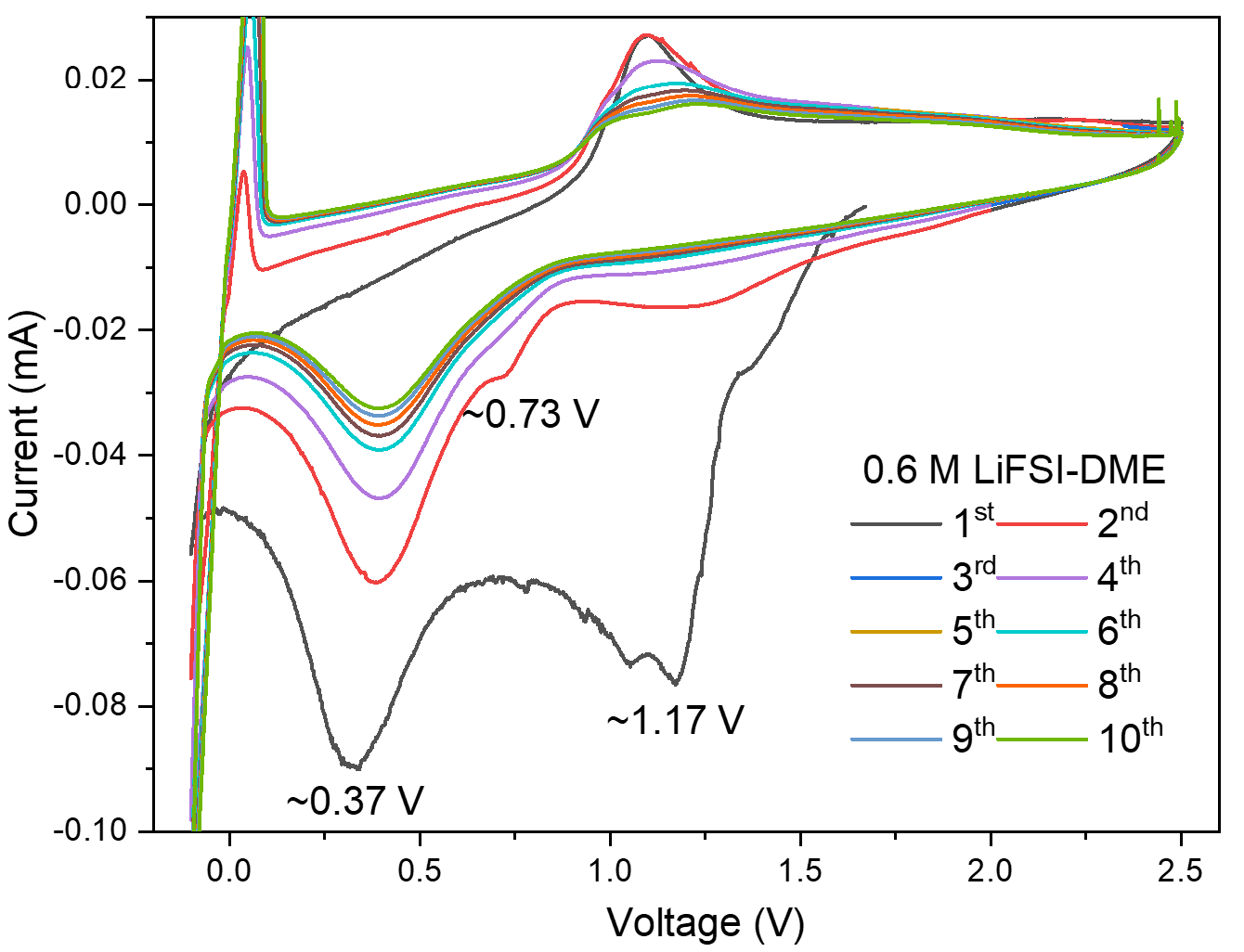


**Supplementary Fig. 16. CV curves of Li||Cu cell in 0.6 M LiFSI-DME electrolyte.** A scan rate of 0.8 mV s^-1^ was applied with a voltage range from -0.1 to 2.5 V vs. Li/Li^+^.


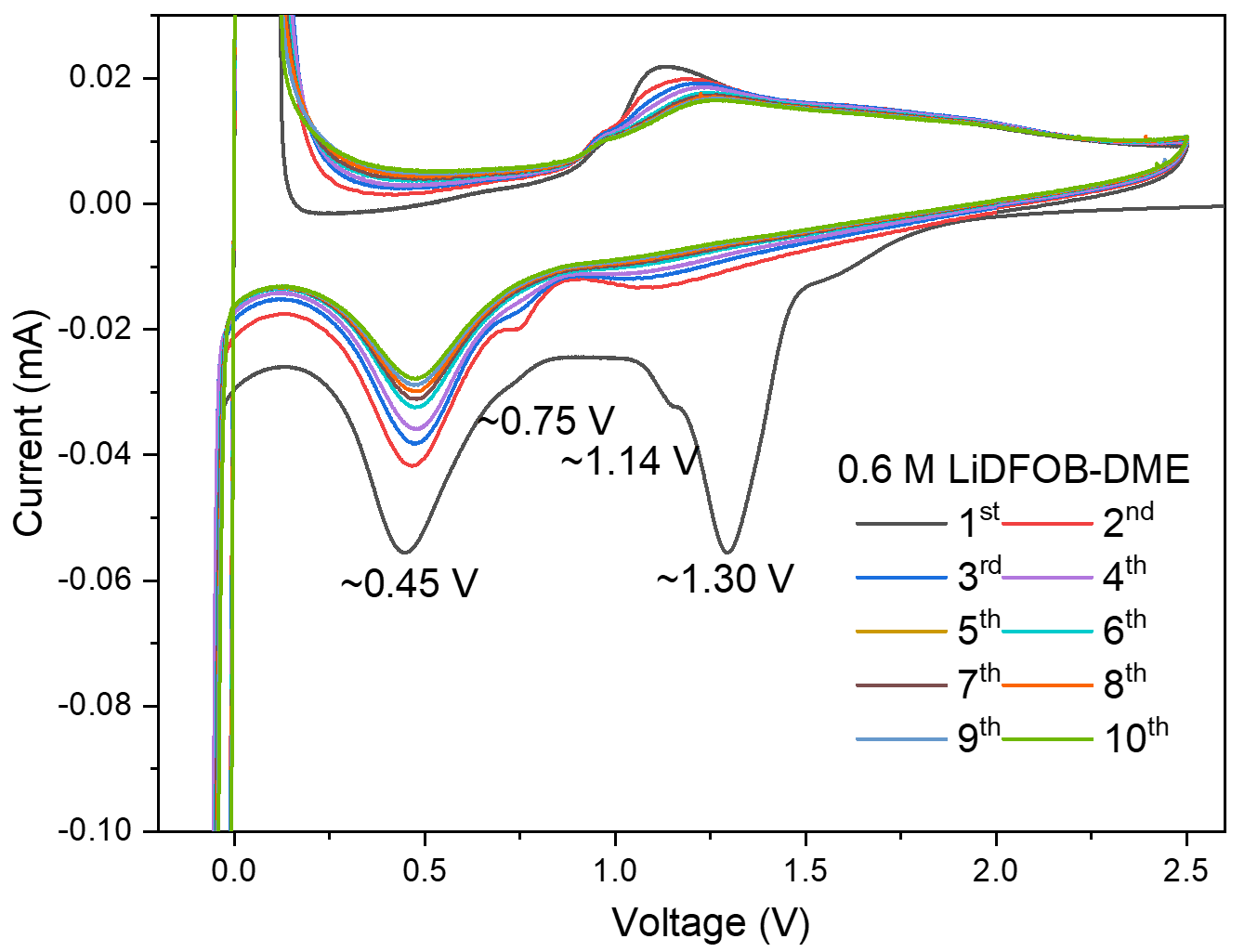


**Supplementary Fig. 17. CV curves of Li||Cu cell in 0.6 M LiDFOB-DME electrolyte.** A scan rate of 0.8 mV s^-1^ was applied with a voltage range from -0.1 to 2.5 V vs. Li/Li^+^.


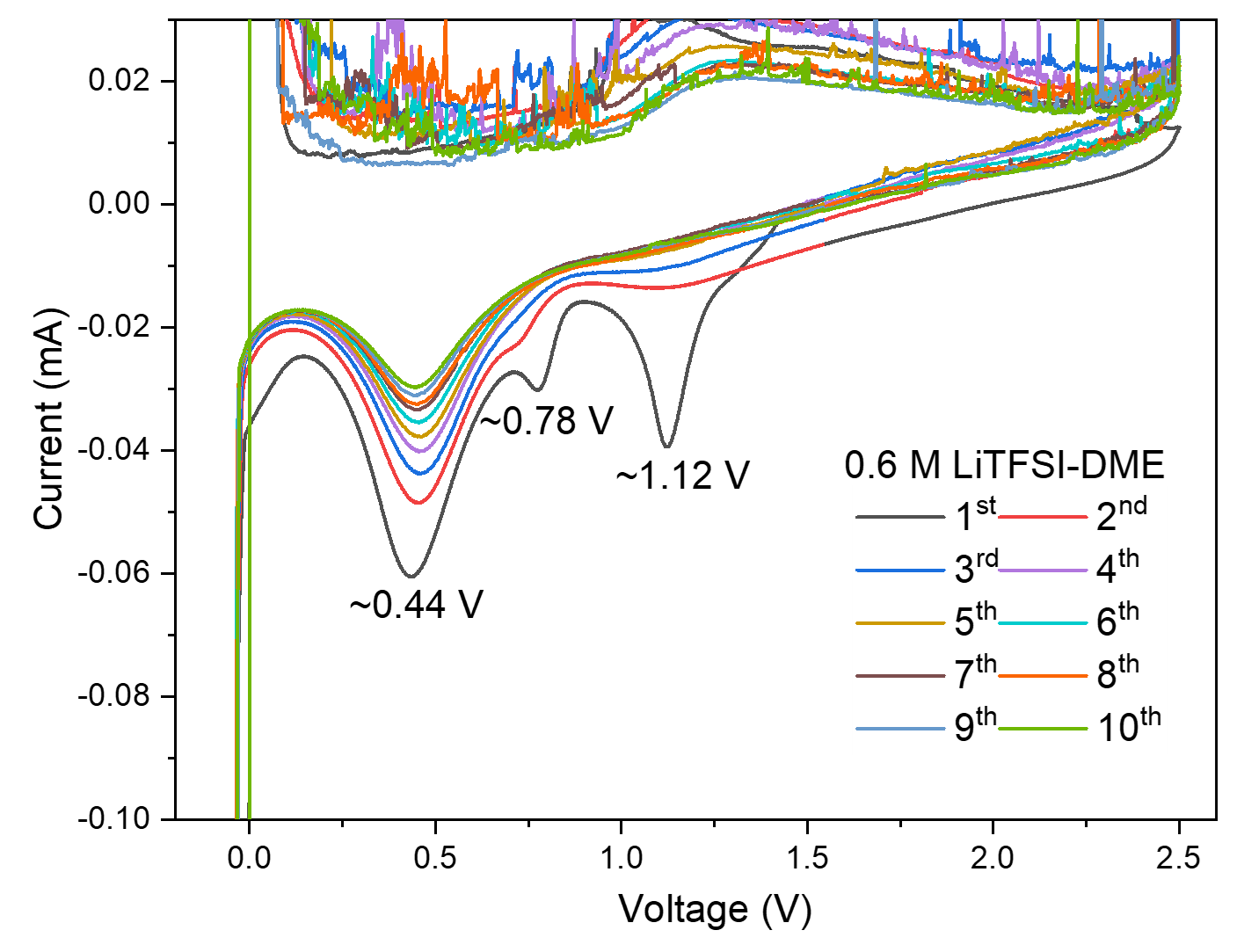


**Supplementary Fig. 18. CV curves of Li||Cu cell in 0.6 M LiTFSI-DME electrolyte.** A scan rate of 0.8 mV s^-1^ was applied with a voltage range from -0.1 to 2.5 V vs. Li/Li^+^.


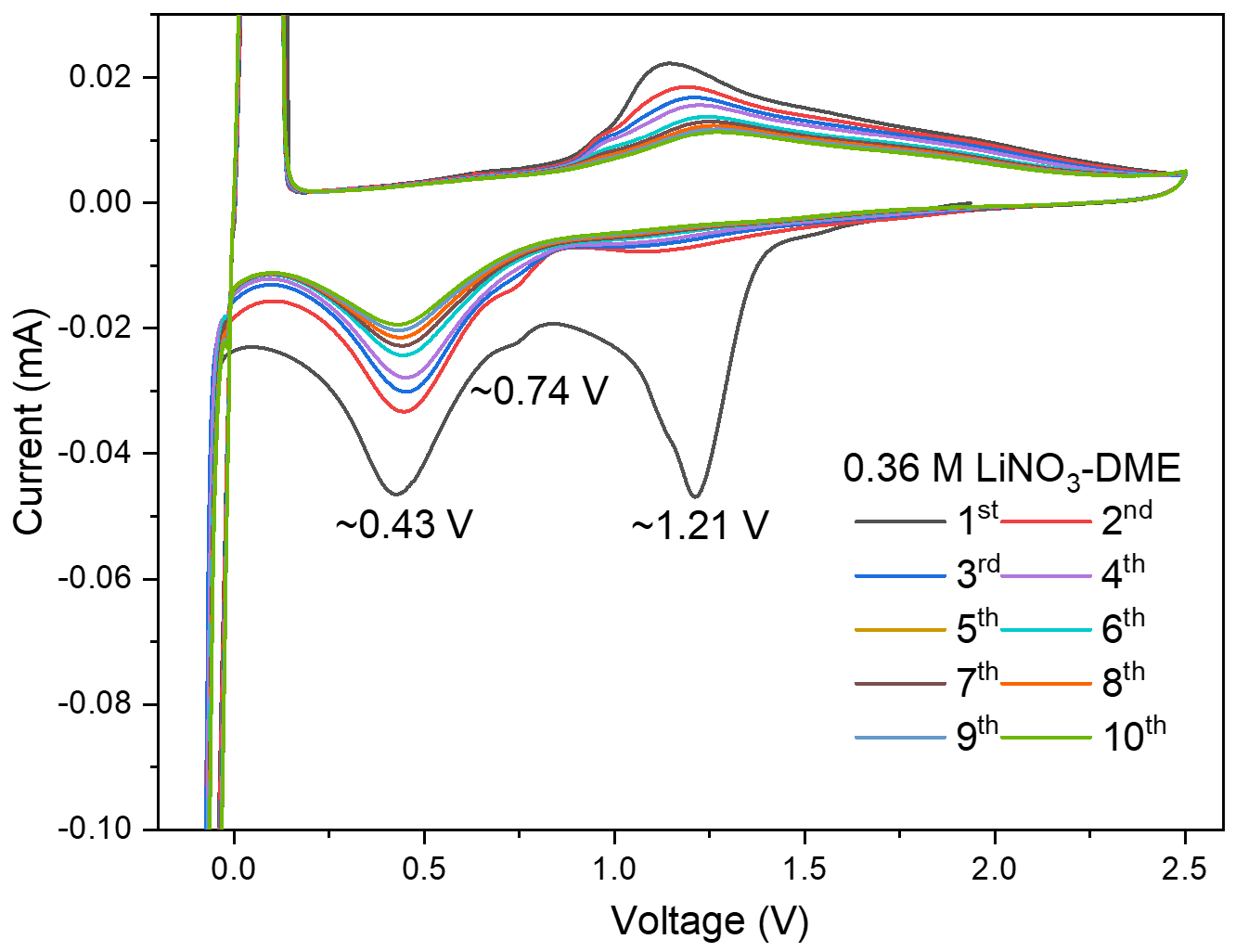


**Supplementary Fig. 19. CV curves of Li||Cu cell in 0.36 M LiNO_3_-DME electrolyte.** A scan rate of 0.8 mV s^-1^ was applied with a voltage range from -0.1 to 2.5 V vs. Li/Li^+^.

**Supplementary Fig. 20. Chronoamperometry profile of symmetric** **Li||Li cells in HE-DME electrolyte under a polarization voltage of 10 mV.** Insets showed the EIS before and after polarization.


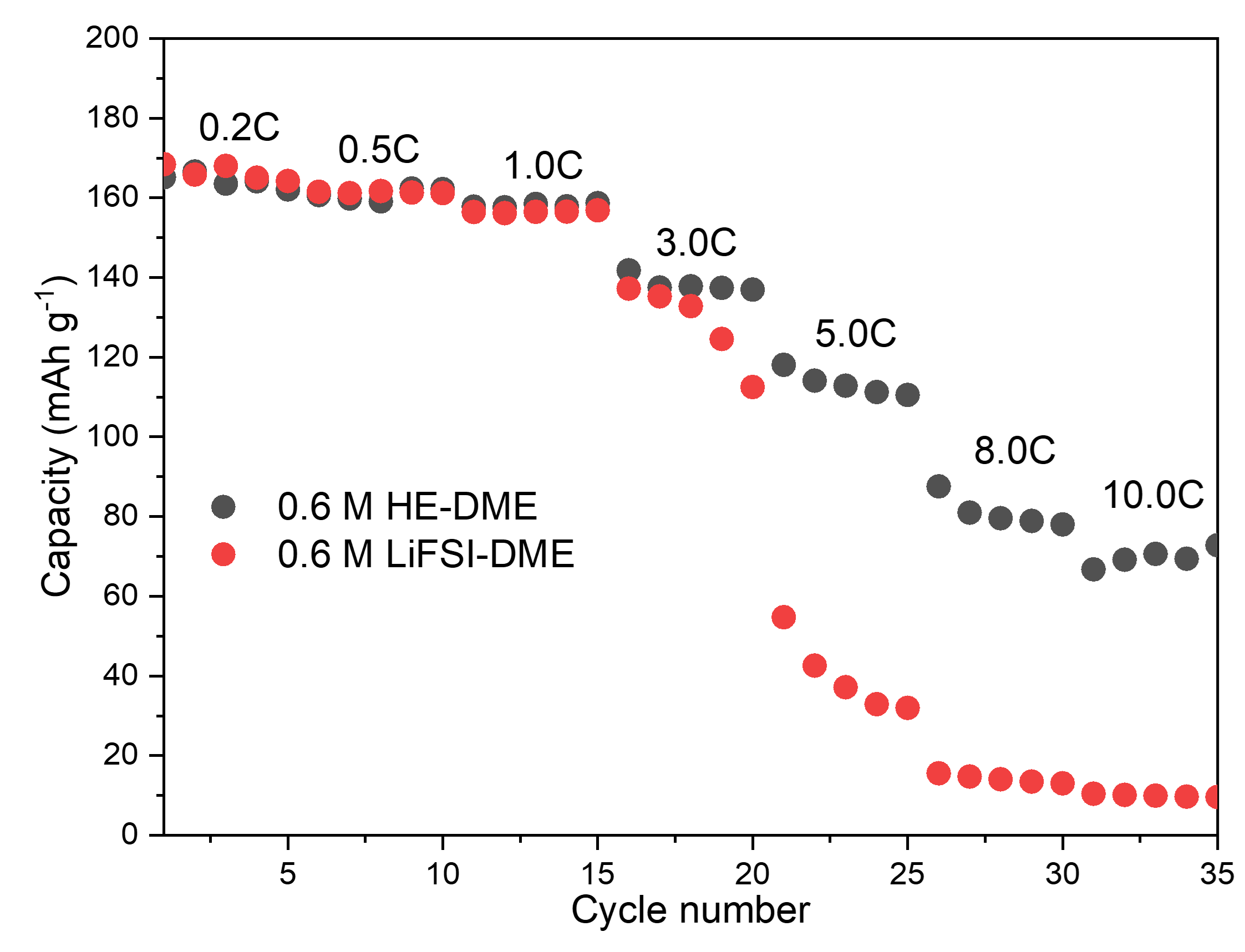


**Supplementary Fig. 21.** **Rate performance of** **Li||Li_4_Ti_5_O_12_ cells.** Cells were evaluated by increasing the charging/discharging rate from 0.2 to 10.0C in different electrolytes within a voltage window of 1.0-2.5 V vs. Li/Li^+^ (1C = 165 mA g^-1^).


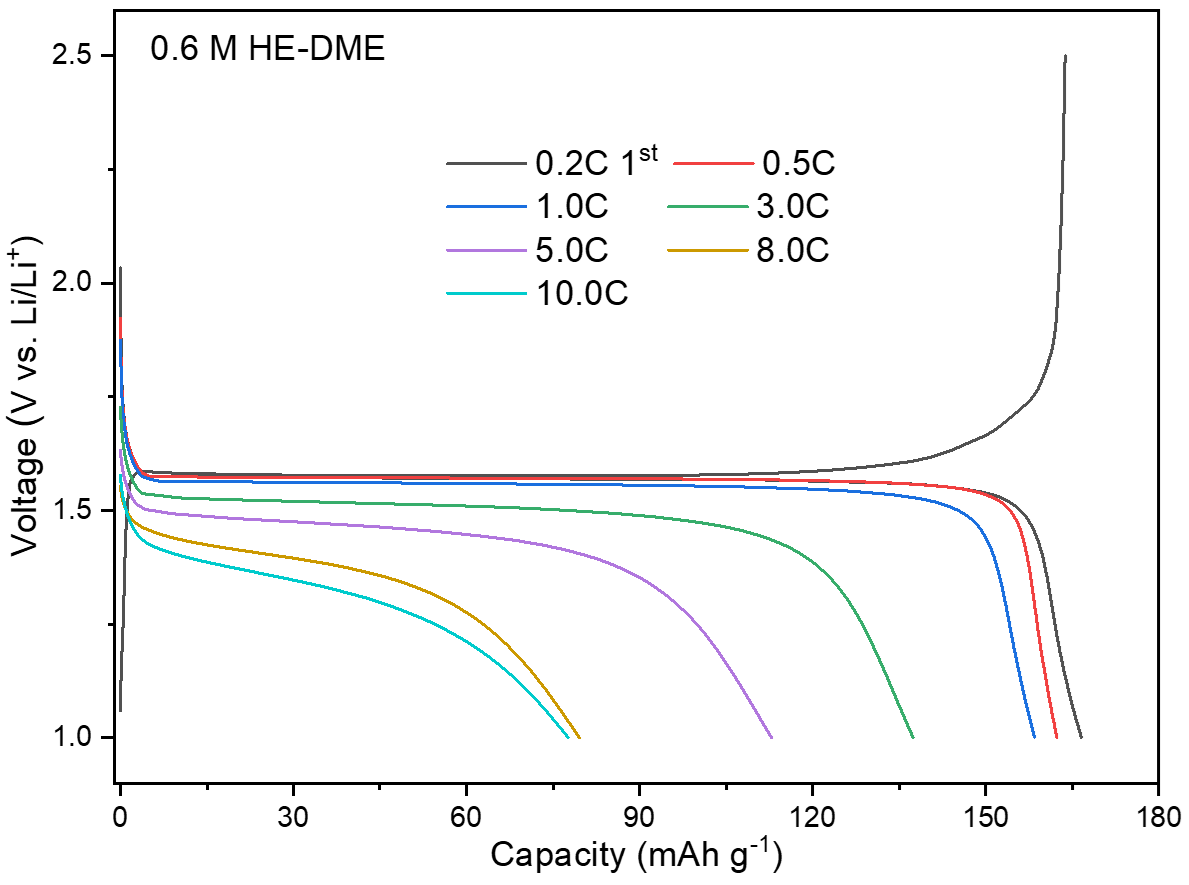


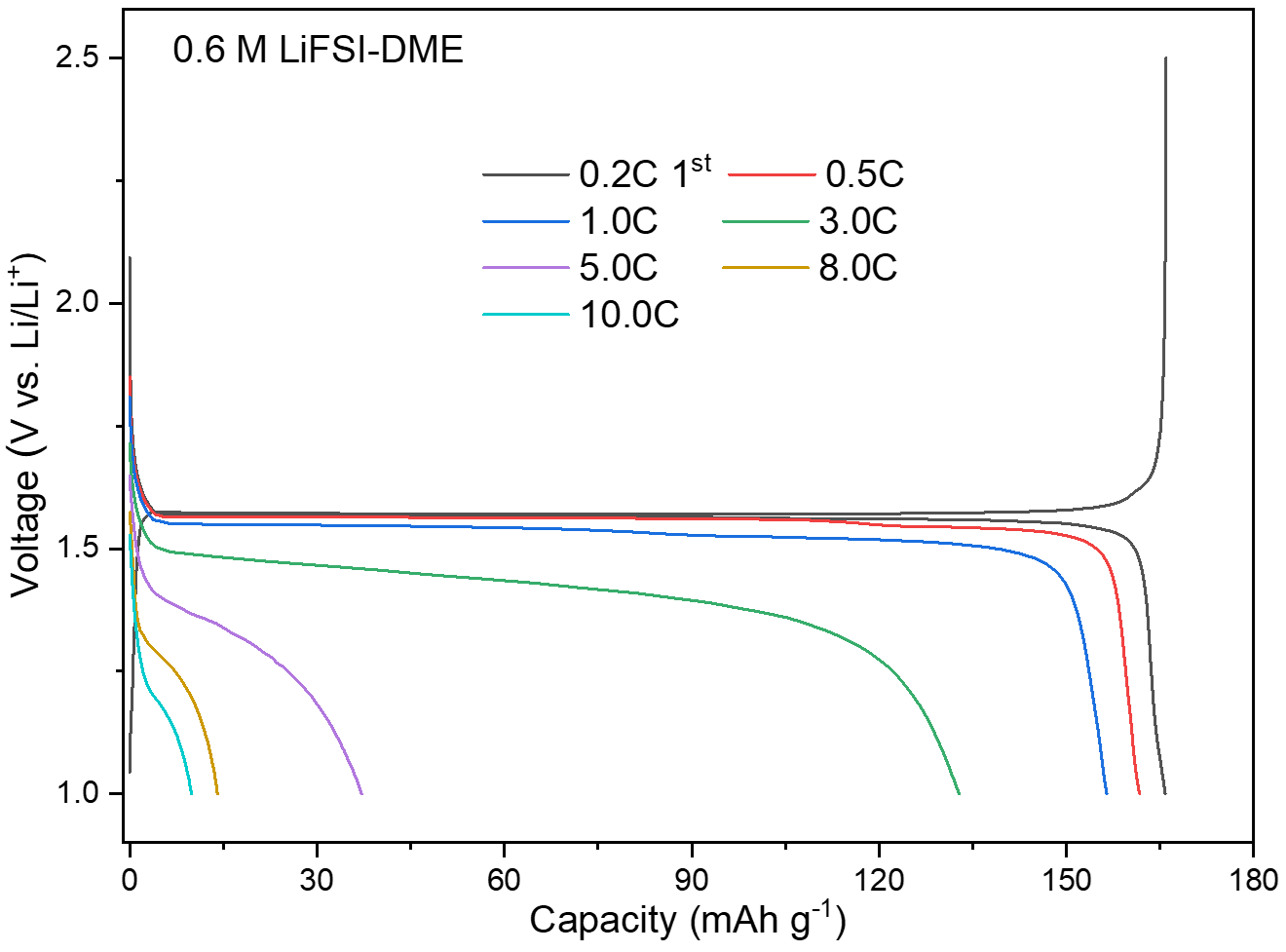


**Supplementary Fig. 22. Galvanostatic charge/discharge curves of Li||Li_4_Ti_5_O_12_ cells**. Cells were evaluated by increasing the charging/discharging rate from 0.2 to 10.0 C in the 0.6 M HE-DME and 0.6 M LiFSI-DME electrolytes within a voltage window of 1.0-2.5 V vs. Li/Li^+^.


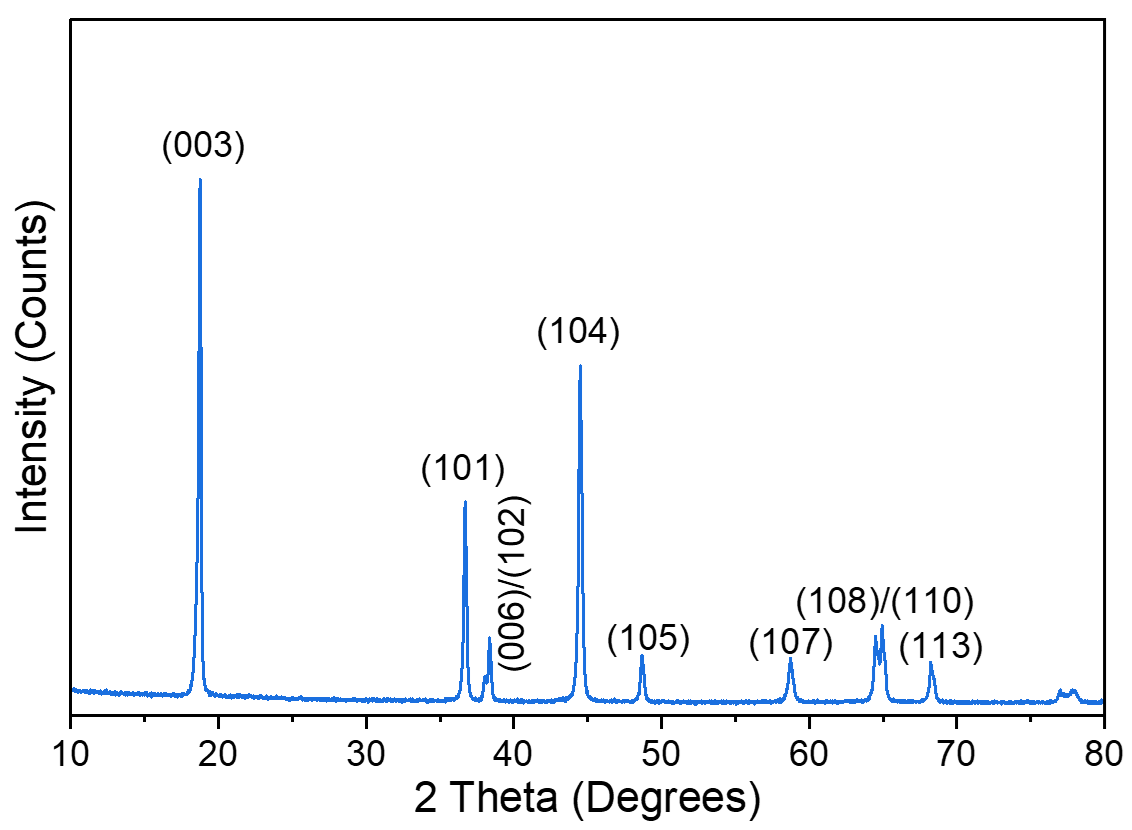


**Supplementary Fig. 23. X-ray diffraction pattern of the prepared NCM811 oxide.**


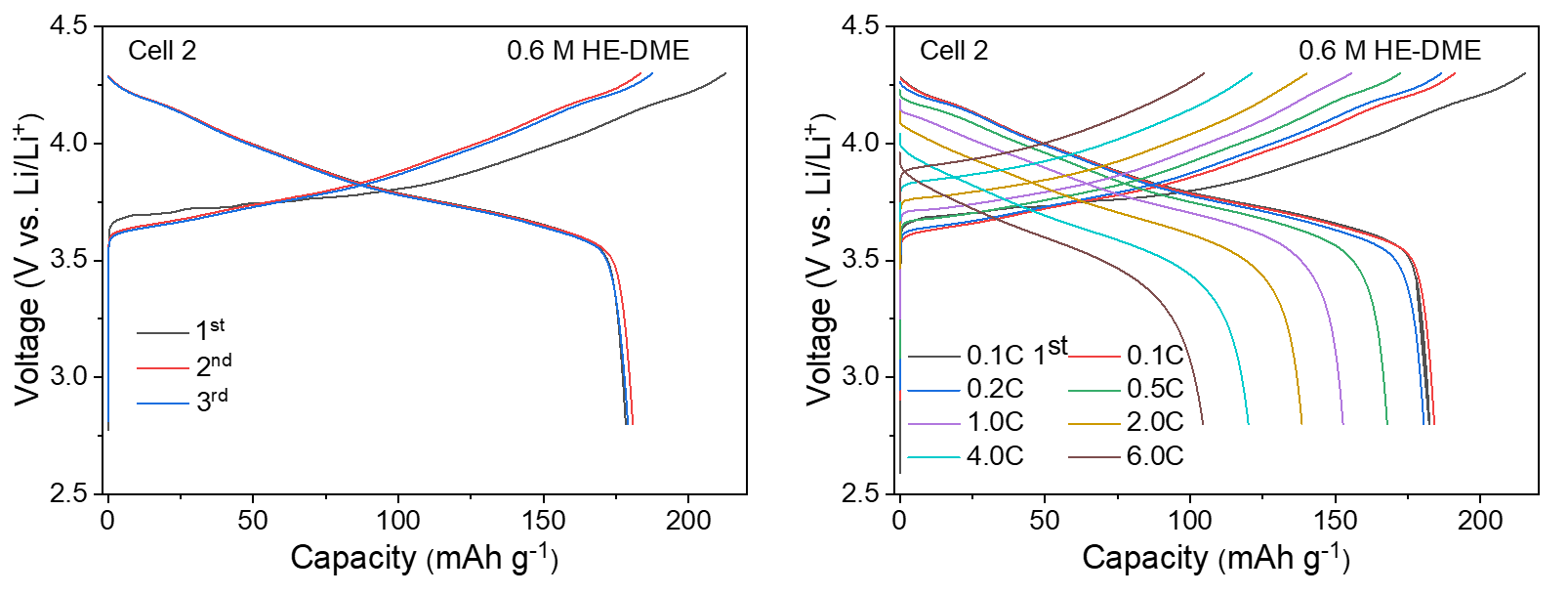


**Supplementary Fig. 24. Galvanostatic charge/discharge curves of Li||NCM811 cells of 0.6 M HE-DME electrolyte.** Cells were cycled at a rate of 0.1C (left) and different rates (right) in the voltage range of 2.8-4.3 V.


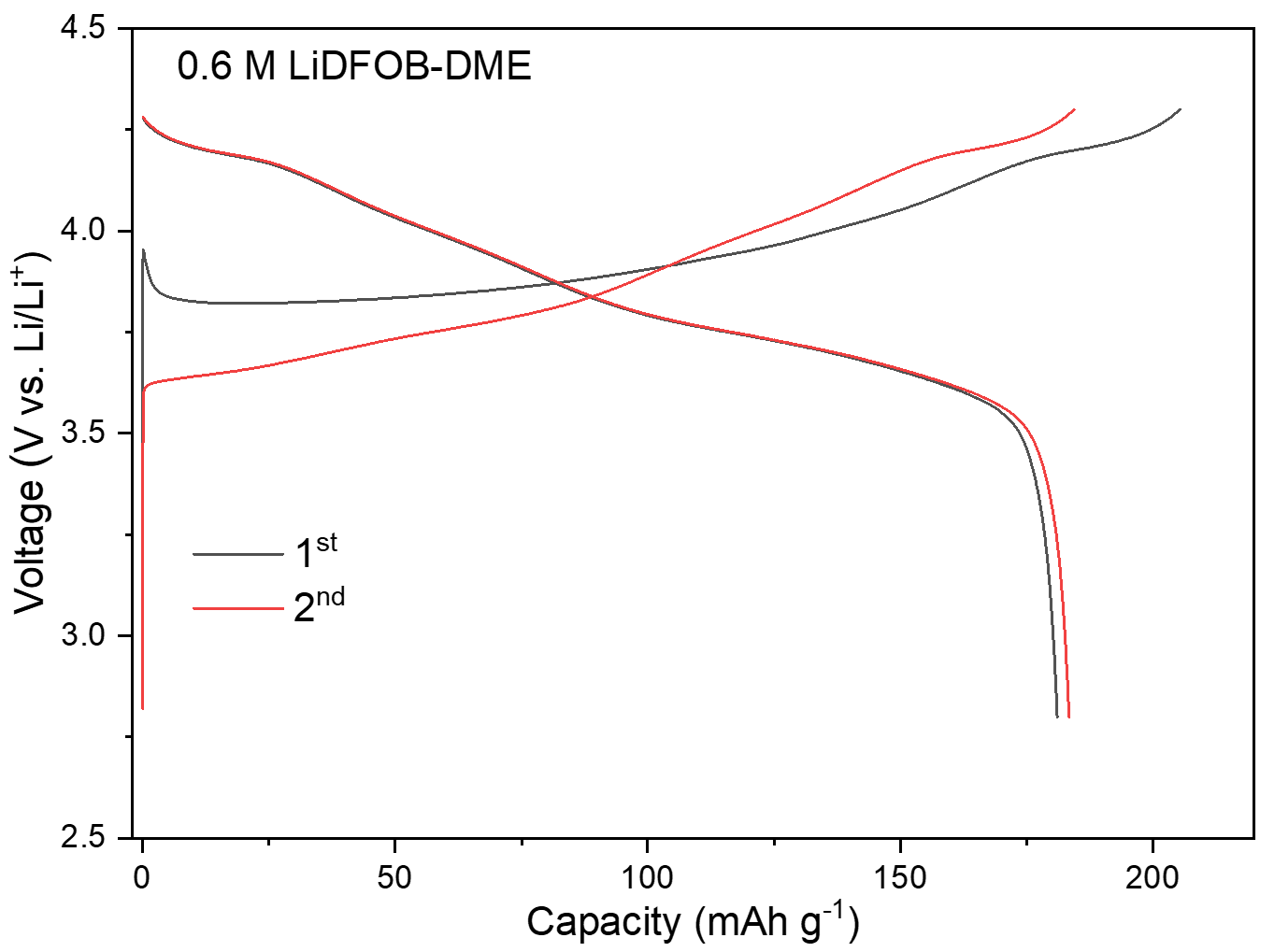


**Supplementary Fig. 25. Galvanostatic charge/discharge curves of Li||NCM811 cells in 0.6 M LiDFOB-DME electrolyte.** Cells were cycled within the voltage range of 2.8-4.3 V at a rate of 0.1C.


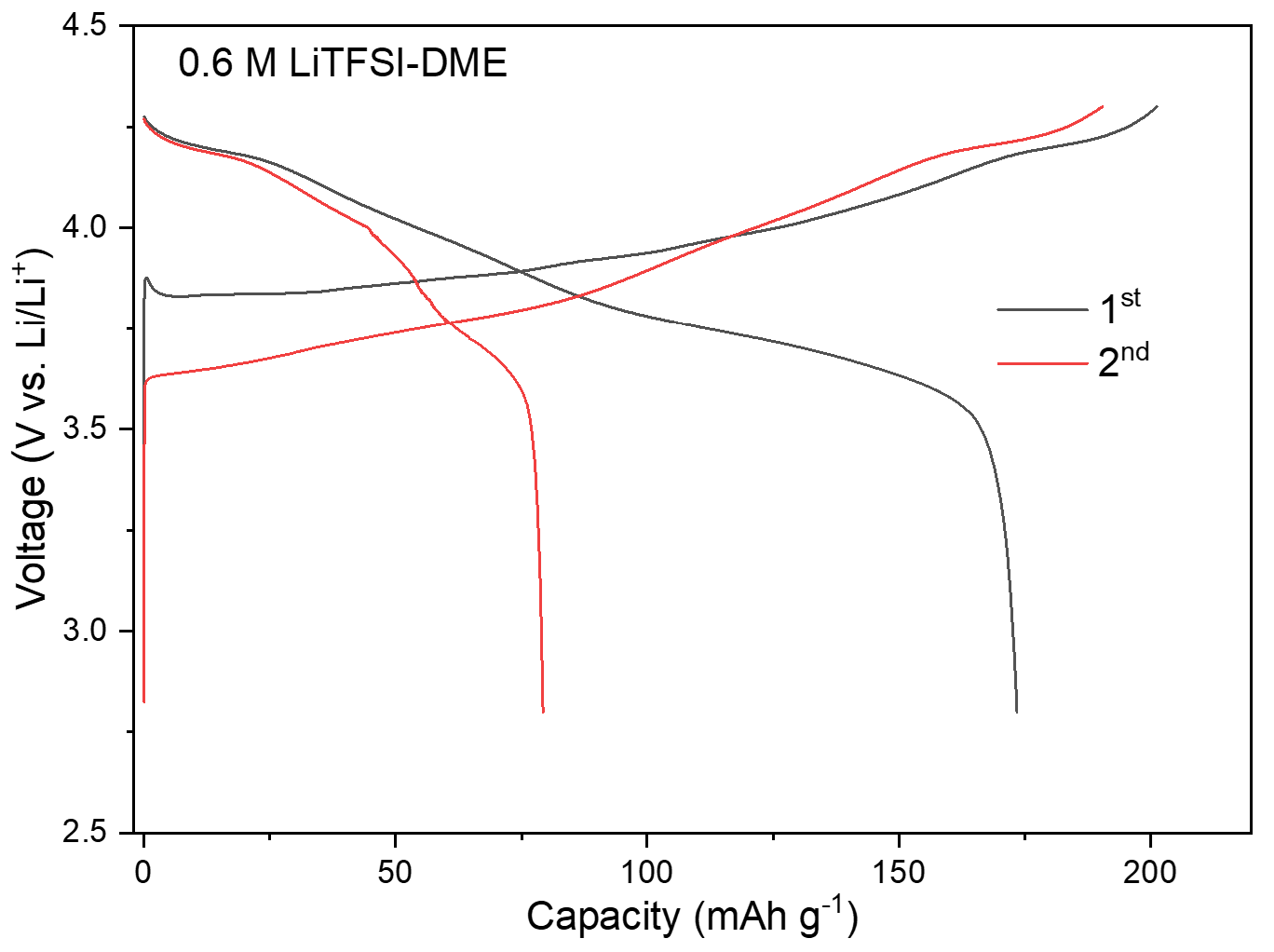


**Supplementary Fig. 26. Galvanostatic charge/discharge curves of Li||NCM811 cells in 0.6 M LiTFSI-DME electrolyte.** Cells were cycled within the voltage range of 2.8-4.3 V at a rate of 0.1C.


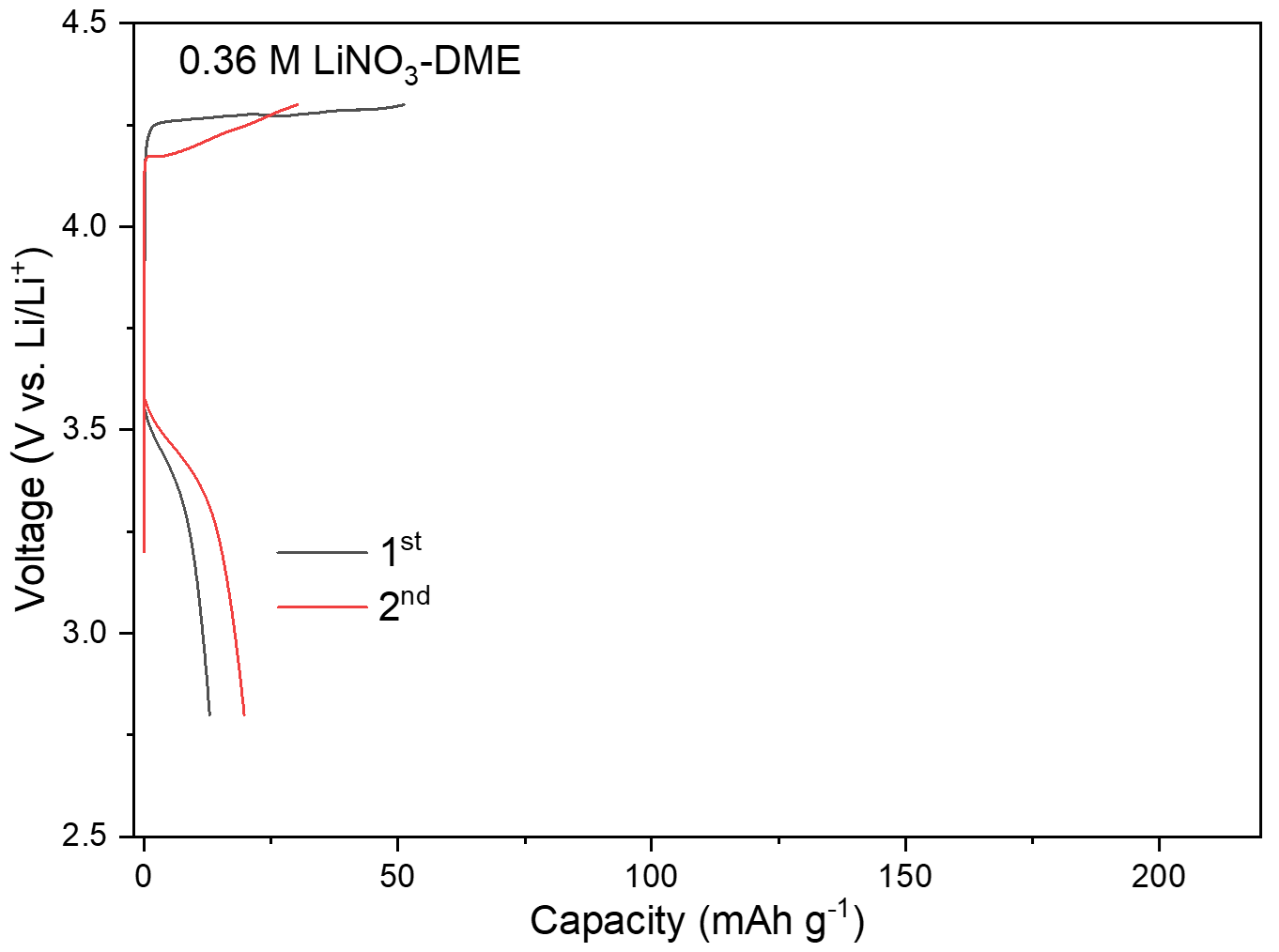


**Supplementary Fig. 27. Galvanostatic charge/discharge** **curves of Li||NCM811 cells in 0.36 M LiNO_3_-DME electrolyte.** Cells were cycled within the voltage range of 2.8-4.3 V at a rate of 0.1C.


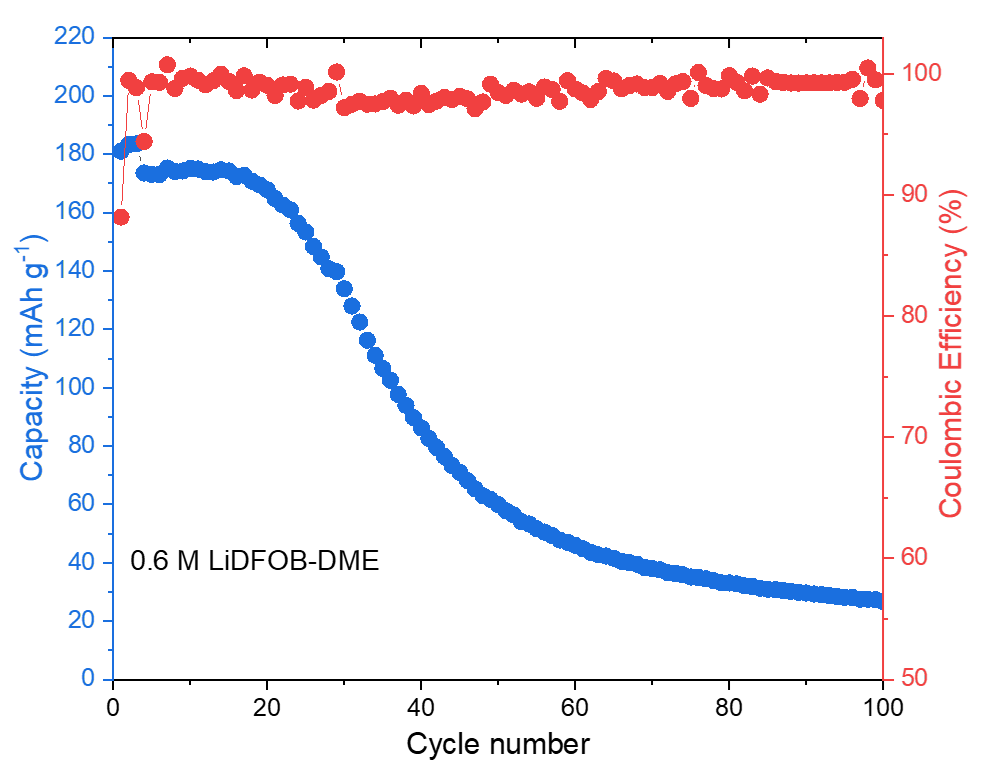


**Supplementary Fig. 28. Cycling performance of Li||NCM811 cells with 0.6 M LiDFOB-DME electrolyte.** Cells were cycled between 2.8 and 4.3 V at a 0.1C rate for three cycles before cycling at a 0.333C rate.


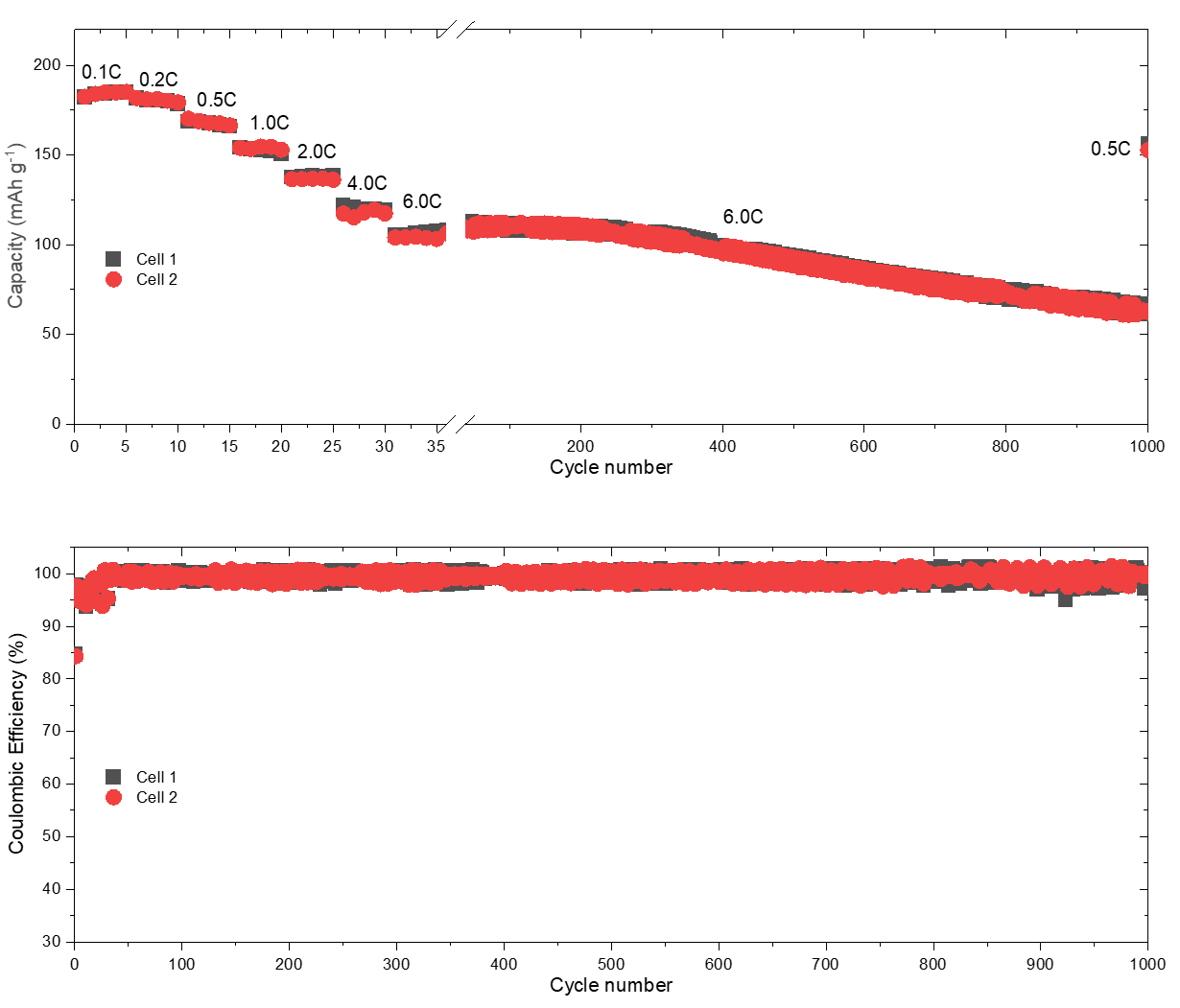


**Supplementary Fig. 29. Electrochemical rate capabilities and CE of Li||NCM811 cells in the 0.6 M HE-DME electrolyte.** Cells were cycled between 2.8 and 4.3 V from 0.1 to 6.0C, after which long-term cycling at 6.0C was performed up to 1000 cycles, and then a recovery cycle of 0.5C was set.


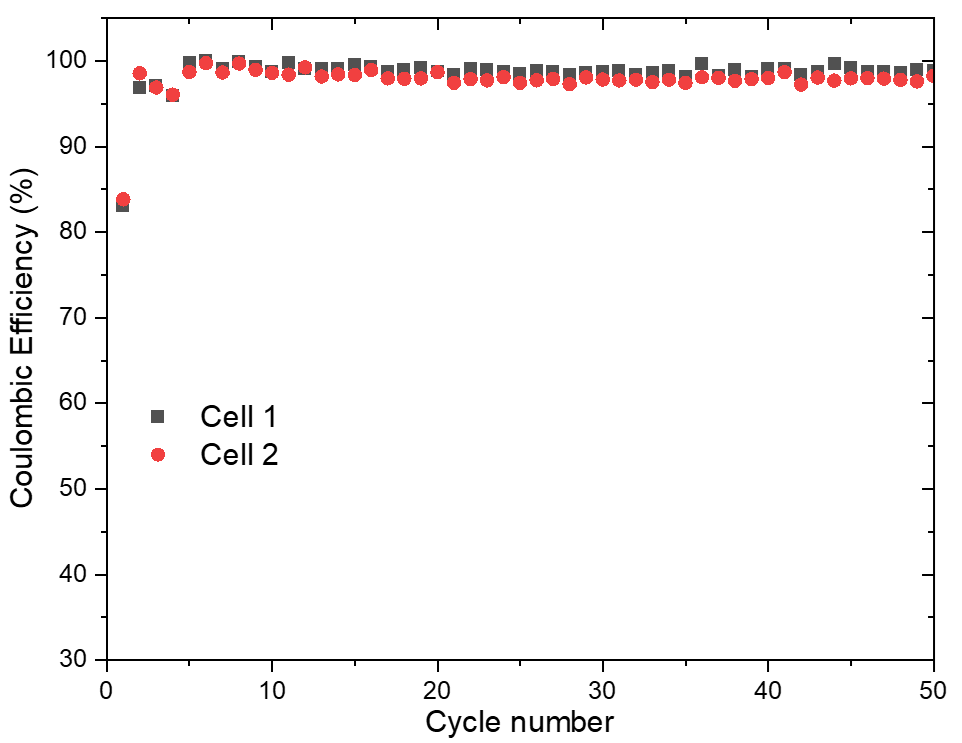


**Supplementary Fig. 30.** **CE of Li||NCM811 cells in the 0.6 M HE-DME electrolyte.** Two representative cells are shown.


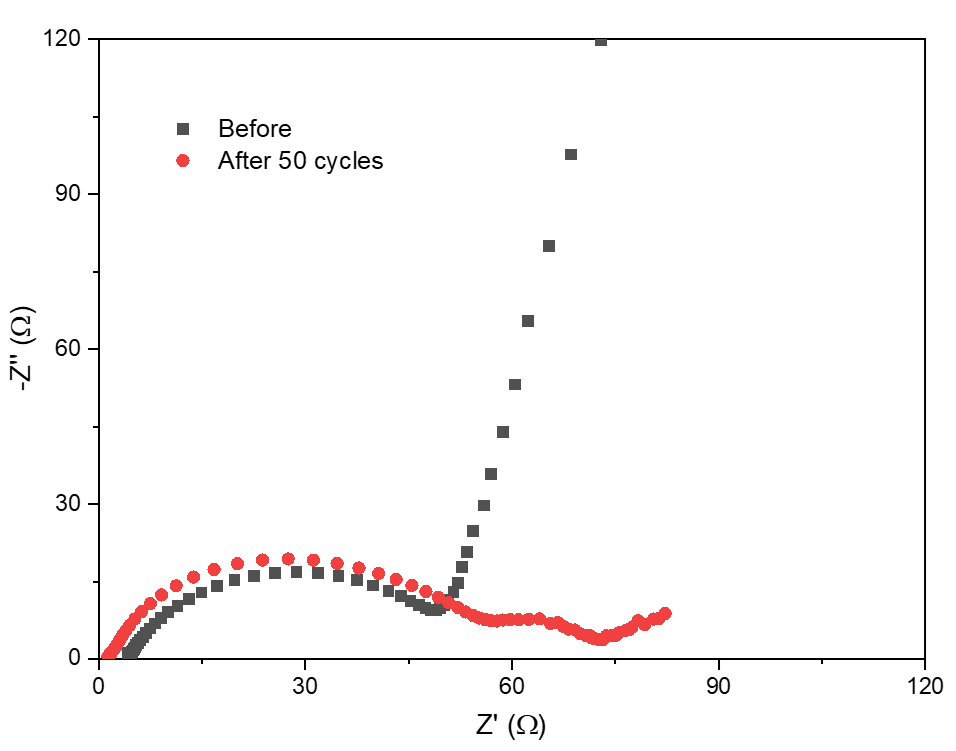


**Supplementary Fig. 31. EIS of Li||NCM811 cells in 0.6 M HE-DME electrolyte** **before and after cycling.**


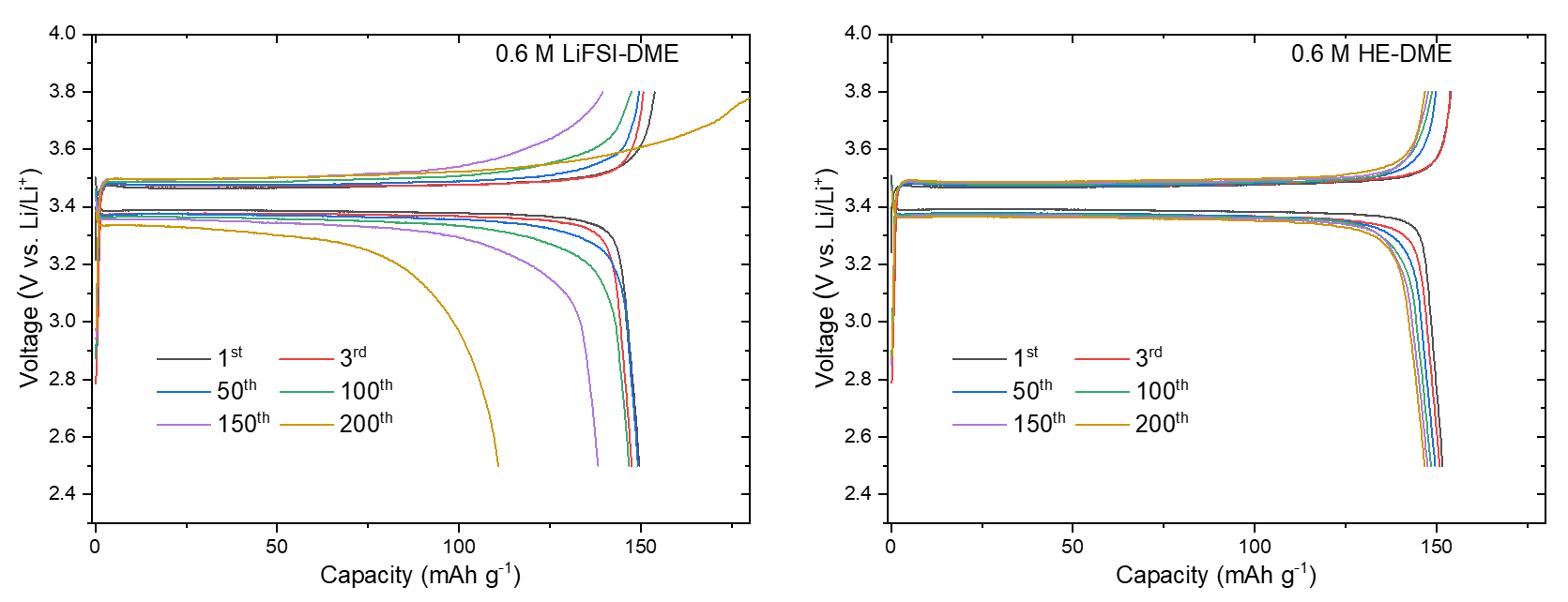


**Supplementary Fig. 32. Galvanostatic charge/discharge curves of Li||LiFePO_4_ cells in electrolytes.** Cells were cycled within the voltage range of 2.5-3.8 V at a rate of 0.2C for three cycles before cycling at a 1.0C rate.


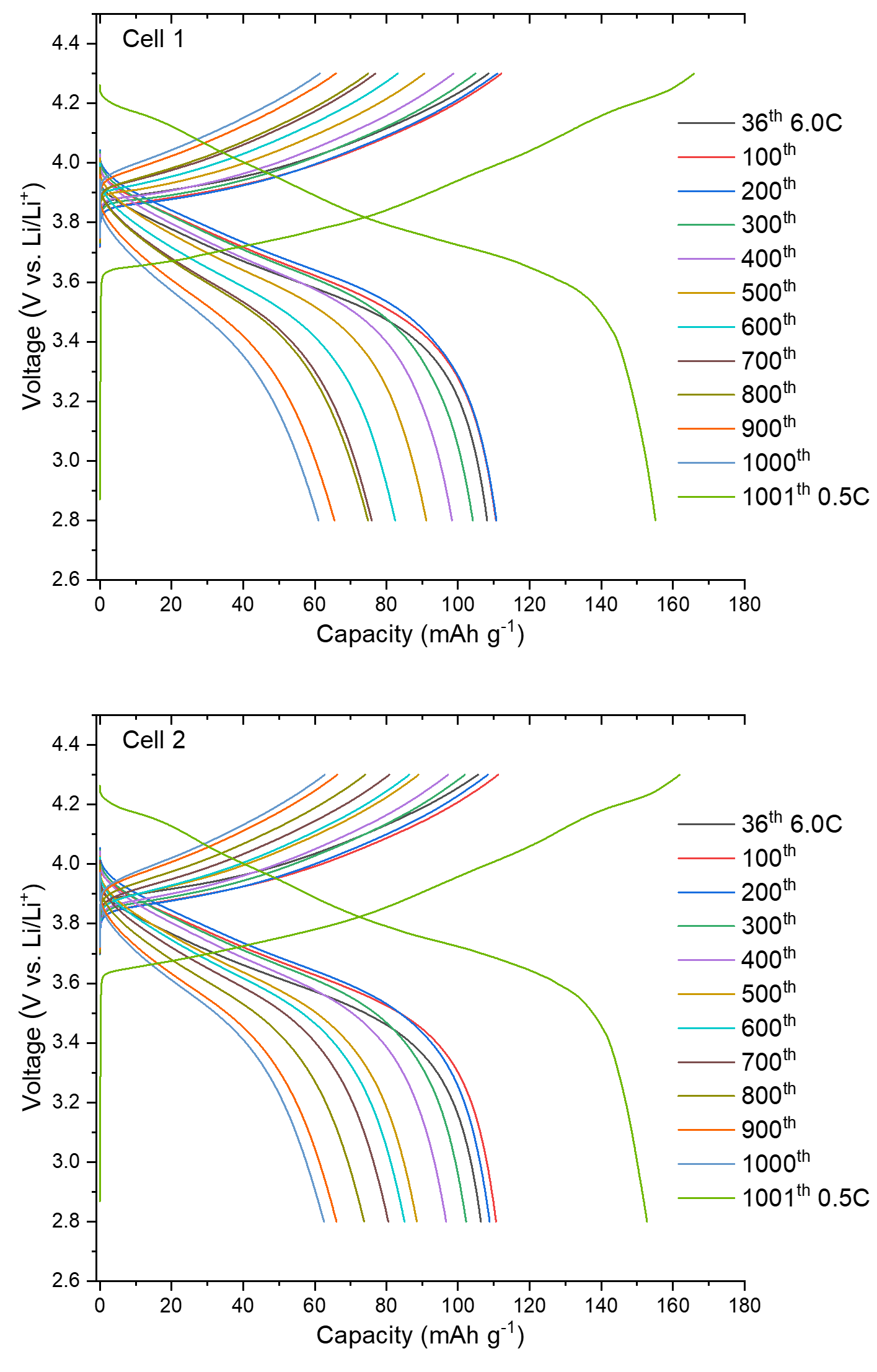


**Supplementary Fig. 33. Galvanostatic charge/discharge curves of the Li||NCM811 cells in the 0.6 M HE-DME electrolyte at a rate of 6.0C.** The 1001^th^ cycle was a recovery cycle performed at 0.5C.


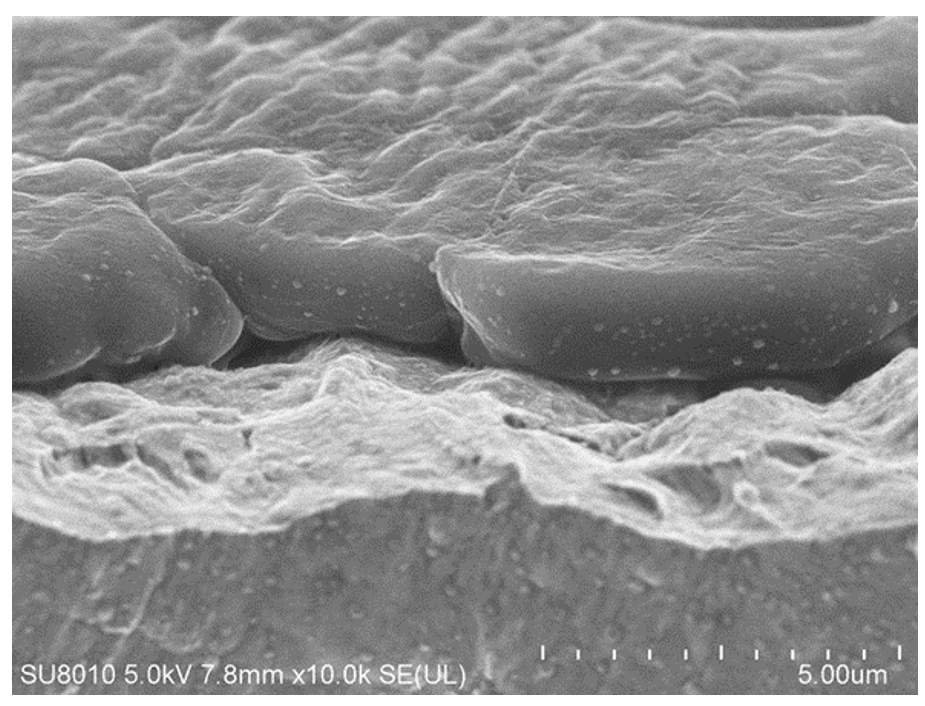


**Supplementary Fig. 34. Scanning electron microscopy (SEM) image of deposited lithium metal in the 0.6 M HE-DME electrolyte from a cross-sectional view.** Cells were cycled at a current density of 0.5 mA cm^-2^ to the capacity of 1 mAh cm^-2^ on Cu foils.


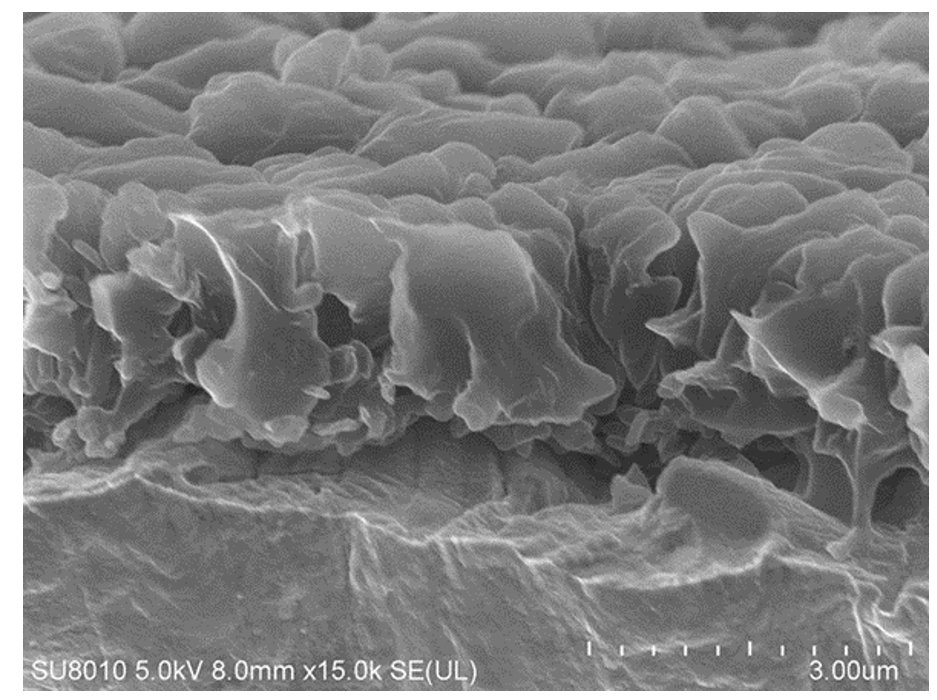


**Supplementary Fig. 35. SEM image of deposited lithium metal in 0.6 M LiFSI-DME electrolyte from a cross-sectional view.** Cells were cycled at a current density of 0.5 mA cm^-2^ to a capacity of 1 mAh cm^-2^ on Cu foils.


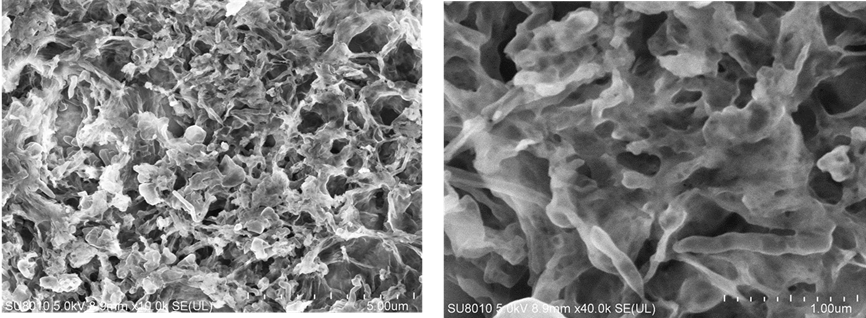


**Supplementary Fig. 36. SEM images of stripped lithium metal in 0.6 M LiFSI-DME electrolyte from a top view.** Cells were cycled at a current density of 0.5 mA cm^-2^ with plating for 2 h followed by stripping to a cut-off voltage of 1.0 V vs. Li/Li^+^.


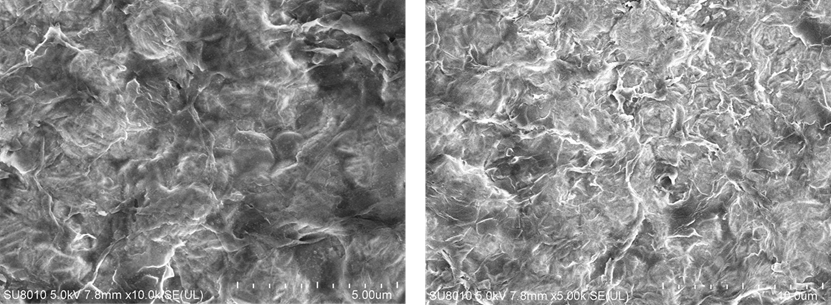


**Supplementary Fig. 37. SEM images of stripped lithium metal in 0.6 M HE-DME electrolyte from a top view.** Cells were cycled at a current density of 0.5 mA cm^-2^ with plating for 2 h followed by stripping to a cut-off voltage of 1.0 V vs. Li/Li^+^.


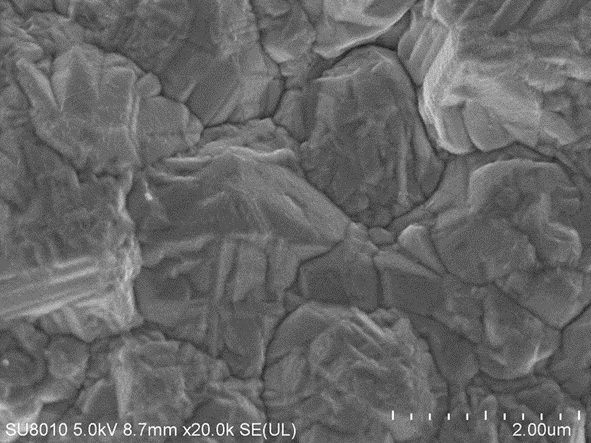


**Supplementary Fig. 38. SEM image of Cu foil.** Top view image of bare Cu foil used for current collector.


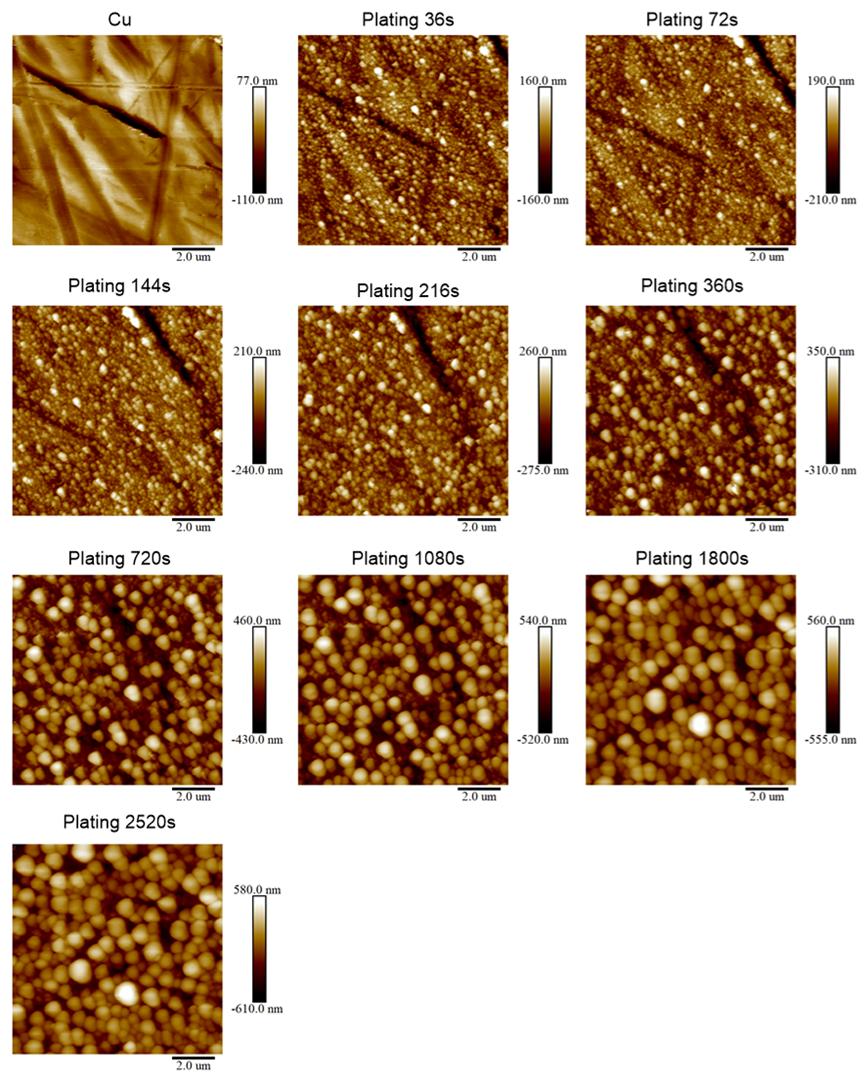


**Supplementary Fig. 39. I****n-situ electrochemical atomic force microscopy (AFM) images of deposited lithium metal in 0.6 M LiFSI-DME electrolyte.** During the electrochemical measurement, cells were discharged at a constant current density of 0.5 mA cm^-2^ in which the images of the lithium plating process were collected at different times.


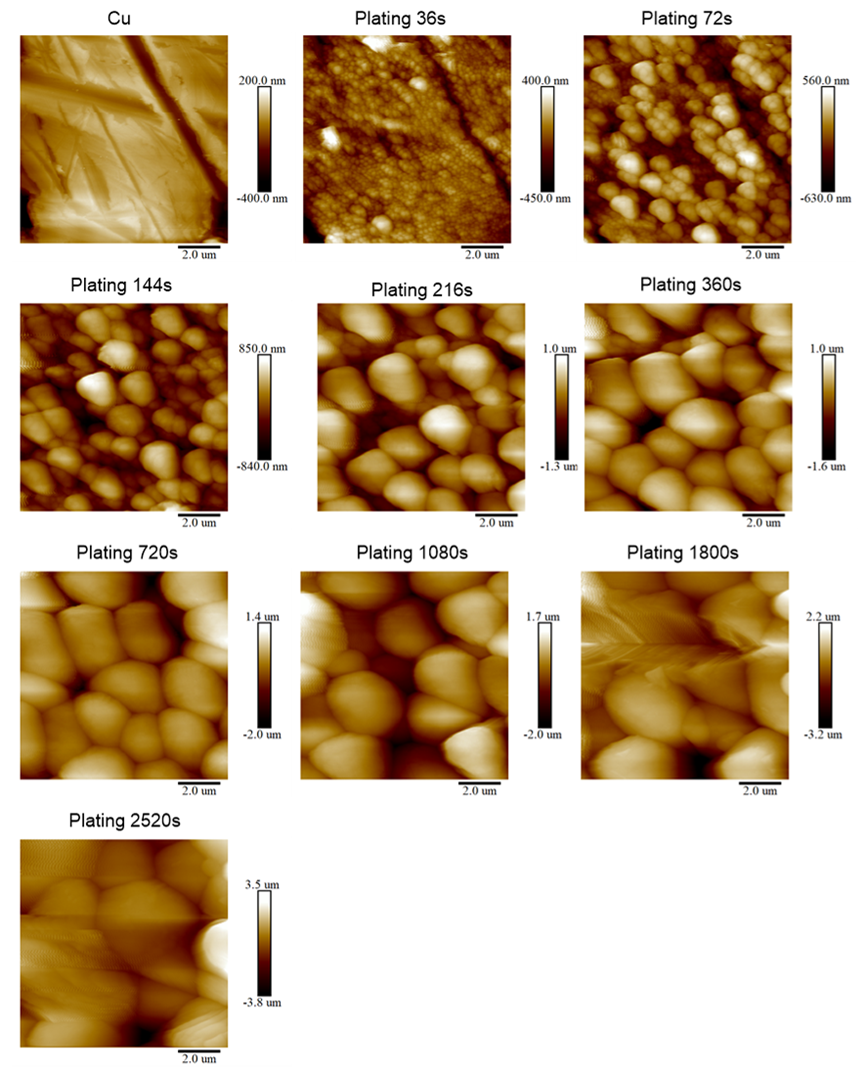


**Supplementary Fig. 40. In-situ electrochemical AFM images of deposited lithium metal in 0.6 M HE-DME electrolyte.** During the electrochemical measurement, cells were discharged at a constant current density of 0.5 mA cm^-2^ in which the images of the lithium plating process were collected at different times.


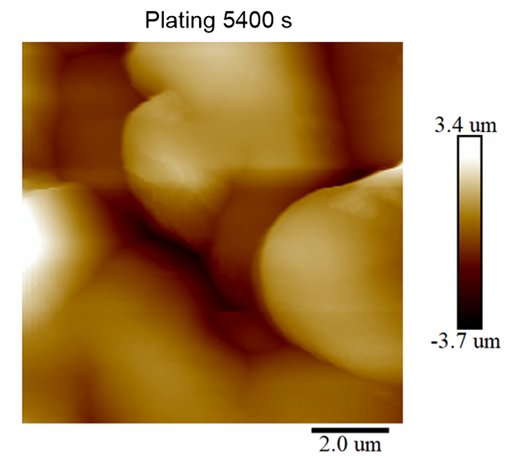


**Supplementary Fig. 41. In-situ electrochemical AFM image of deposited lithium metal in 0.6 M HE-DME electrolyte.** Image of deposited lithium metal was collected after 5400 s of deposition.


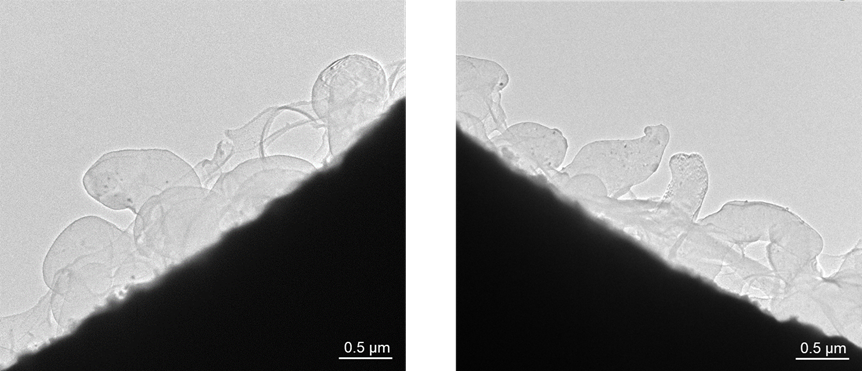


**Supplementary Fig. 42. Microstructure of deposited lithium metal from cryo-transmission electron microscopy (Cryo-TEM) using the 0.6 M HE-DME electrolyte.**


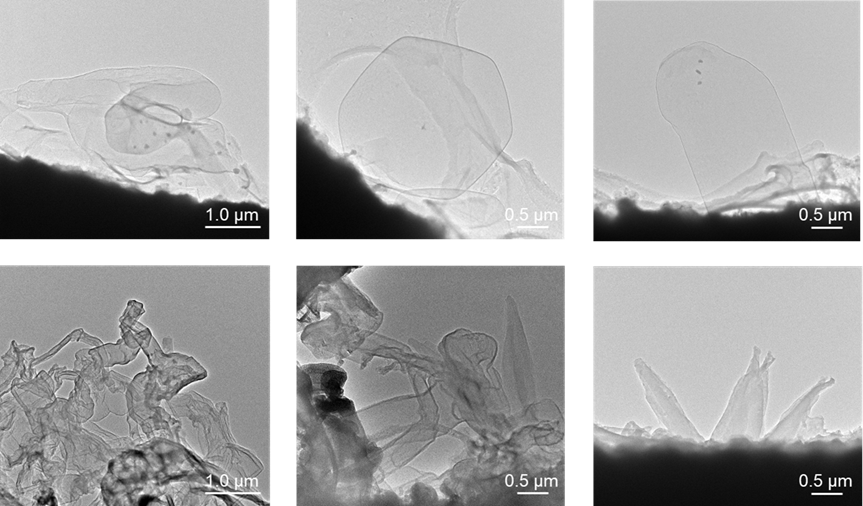


**Supplementary Fig. 43. Microstructure of deposited lithium metal from cryo-TEM images recorded from different sites.** The top images were from the 0.6 M HE-DME electrolyte and the bottom were from the 0.6 M LiFSI-DME electrolyte.


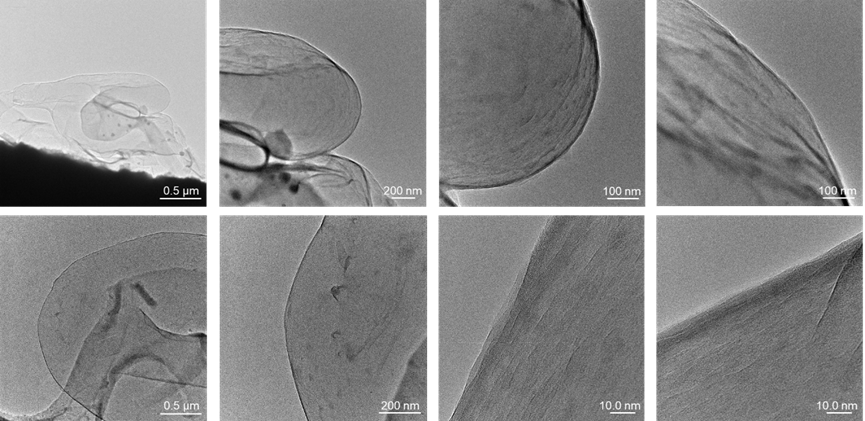


**Supplementary Fig. 44. Microstructure of deposited lithium metal from cryo-TEM images recorded from different sites.** Images were recorded on Lithium metal deposited in the 0.6 M HE-DME electrolyte.


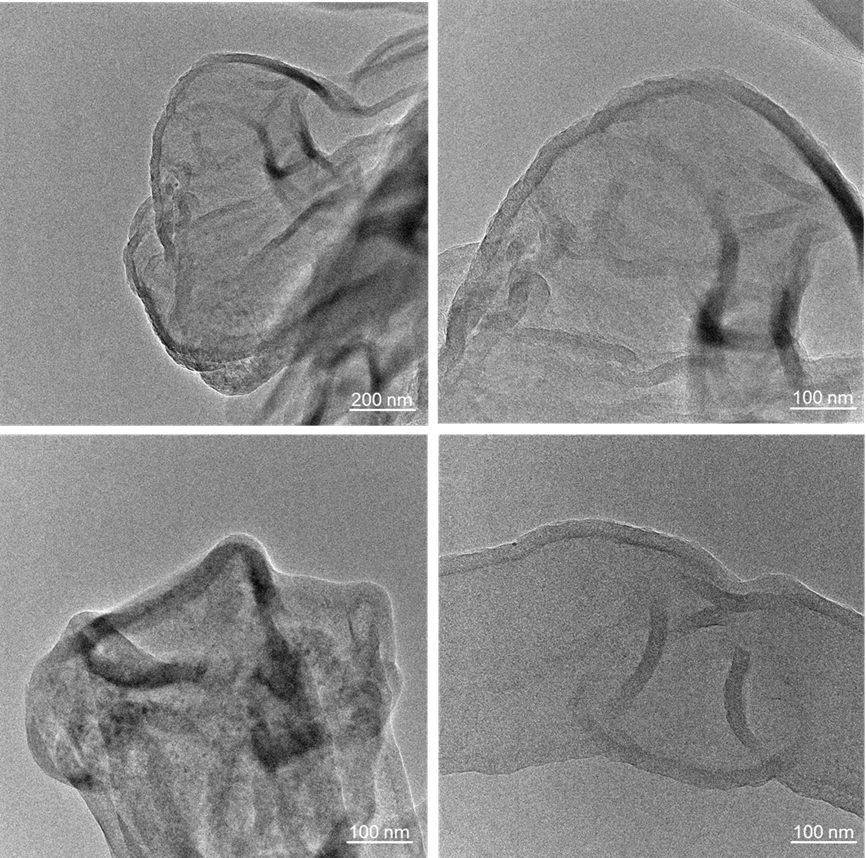


**Supplementary Fig. 45. Microstructure of deposited lithium metal from cryo-TEM images recorded from different sites.** Images were recorded on using the 0.6 M LiFSI-DME electrolyte.


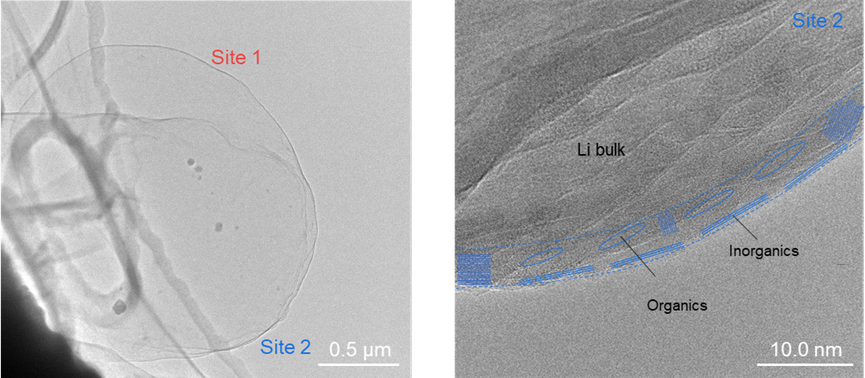


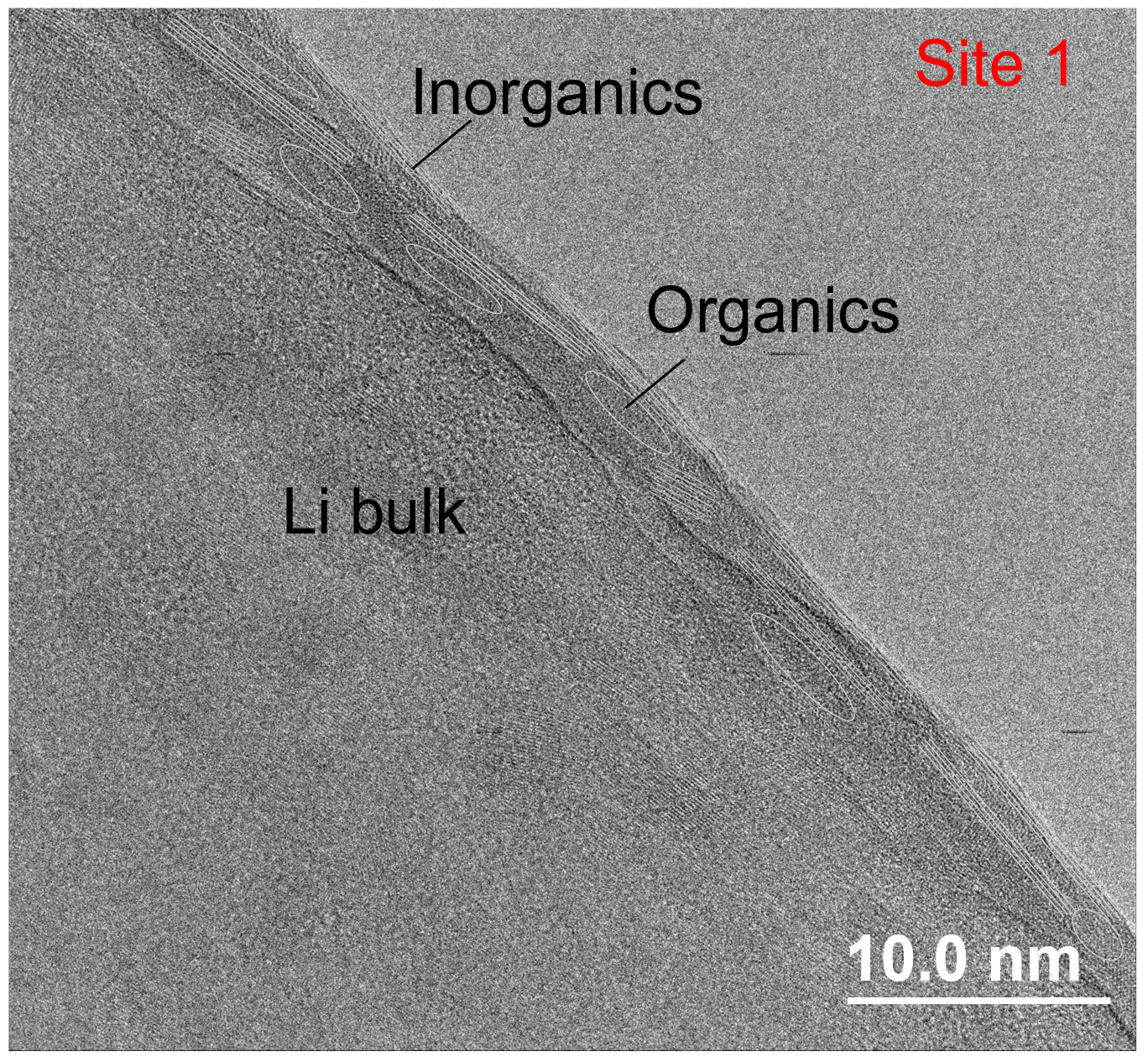


**Supplementary Fig. 46. Microstructure of deposited lithium metal and interfacial phase from cryo-TEM images on using the 0.6 M HE-DME electrolyte.** The left panel is the microstructure and the right is the solid electrolyte interphase (SEI), the insets showing the distribution of inorganics and organics in the SEI. Site 1 is shown in **Fig. 2f**.


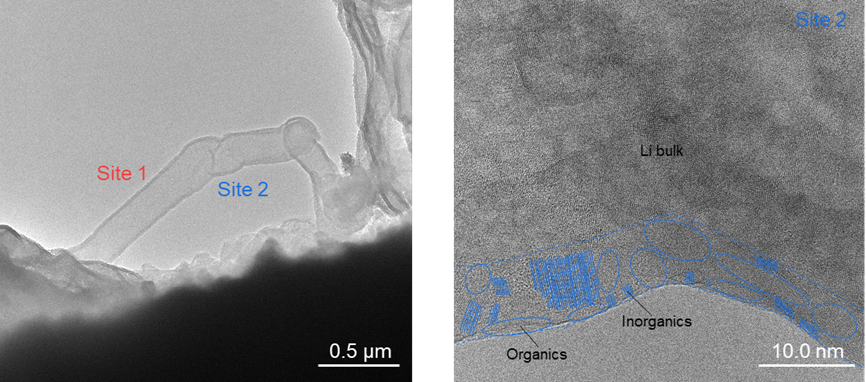


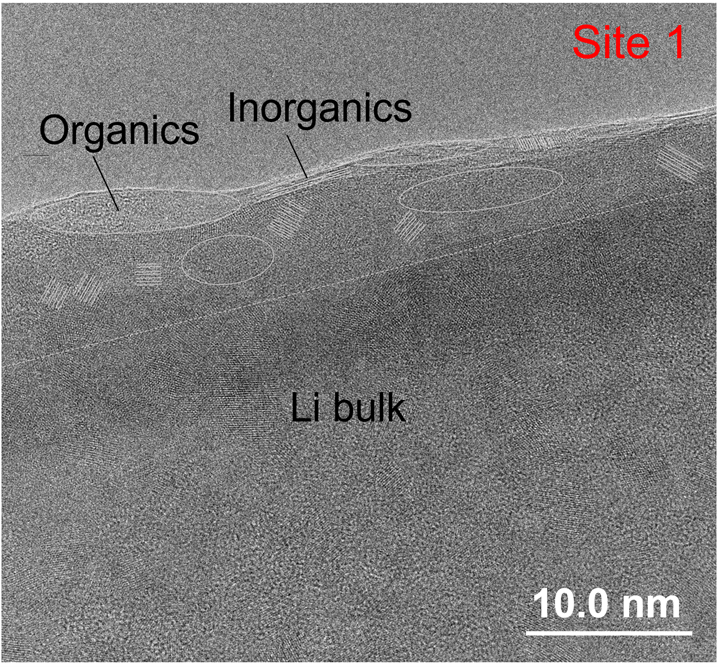


**Supplementary Fig. 47. Microstructure of deposited lithium metal and interfacial phase from cryo-TEM images on using a 0.6 M LiFSI-DME electrolyte.** The left panel is the microstructure and the right is the SEI, the insets showing the distribution of inorganics and organics in the SEI. Site 1 is shown in **Figure 2h**.


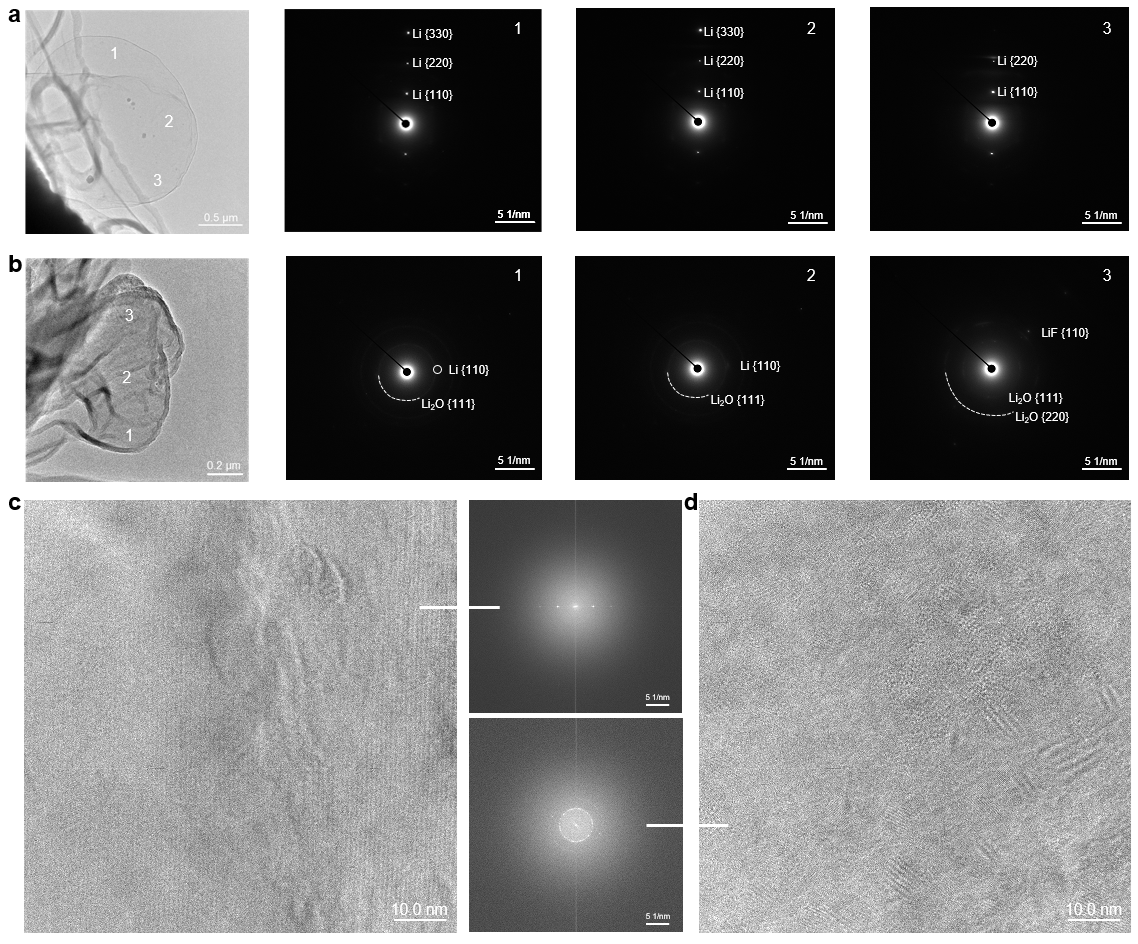


**Supplementary Fig. 48. Structures of deposited lithium metal from cryo-TEM images. a,** Images of deposited lithium metal from 0.6 M HE-DME electrolyte and its selected area electron diffraction (SAED) at different sites. **b,** Images of deposited lithium metal from 0.6 M LiFSI-DME electrolyte and its SAED at different sites. High-resolution TEM images of deposited lithium metal in **c**, 0.6 M HE-DME electrolyte and **d**, 0.6 M LiFSI-DME electrolyte and the corresponding SAED.


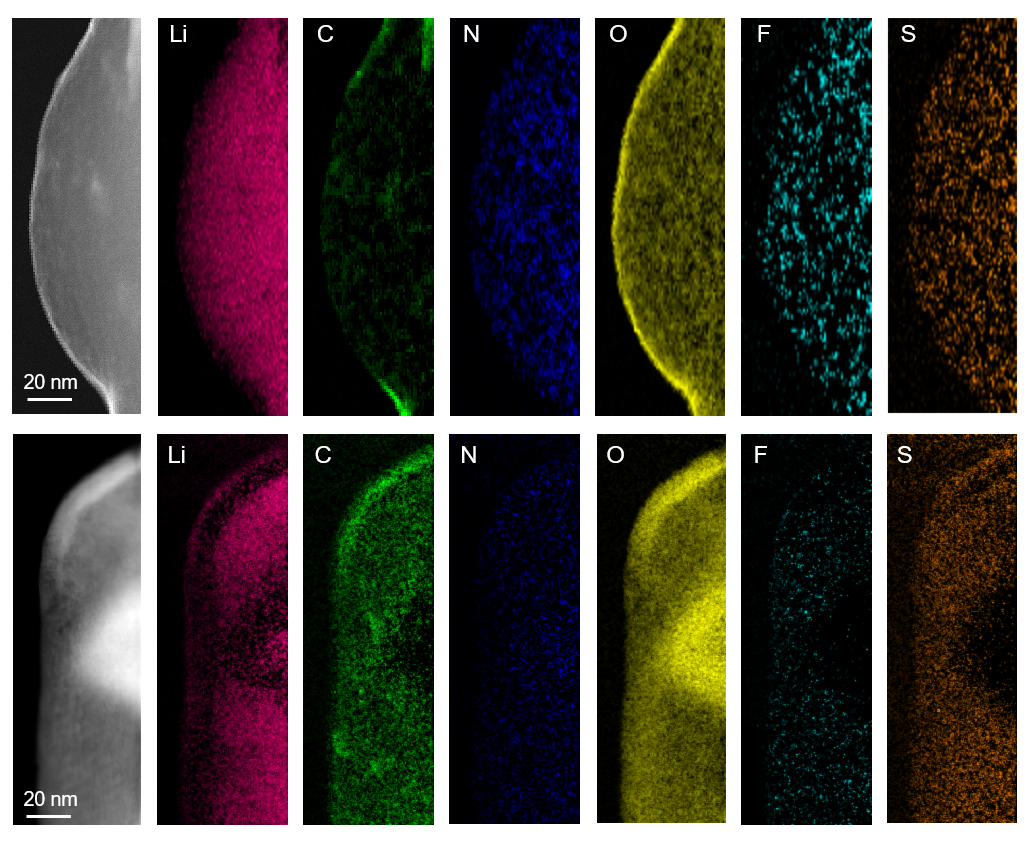


**Supplementary Fig. 49.** **Electron energy loss spectroscopy (EELS) mapping of deposited lithium metal.** Top panels are from the 0.6 M HE-DME electrolyte and the bottom are from the 0.6 M LiFSI-DME electrolyte.


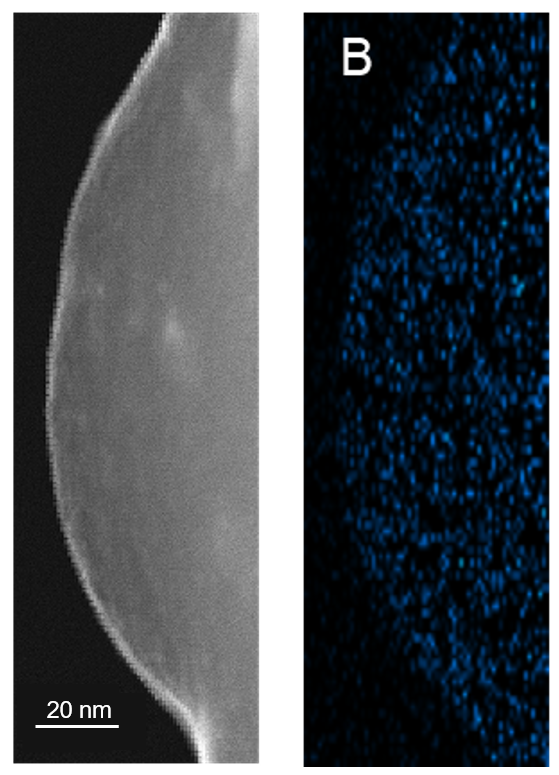


**Supplementary Fig. 50. Cryo-STEM EELS mapping of deposited lithium metal.** Image of B from LiDFOB salt in 0.6 M HE-DME electrolyte.


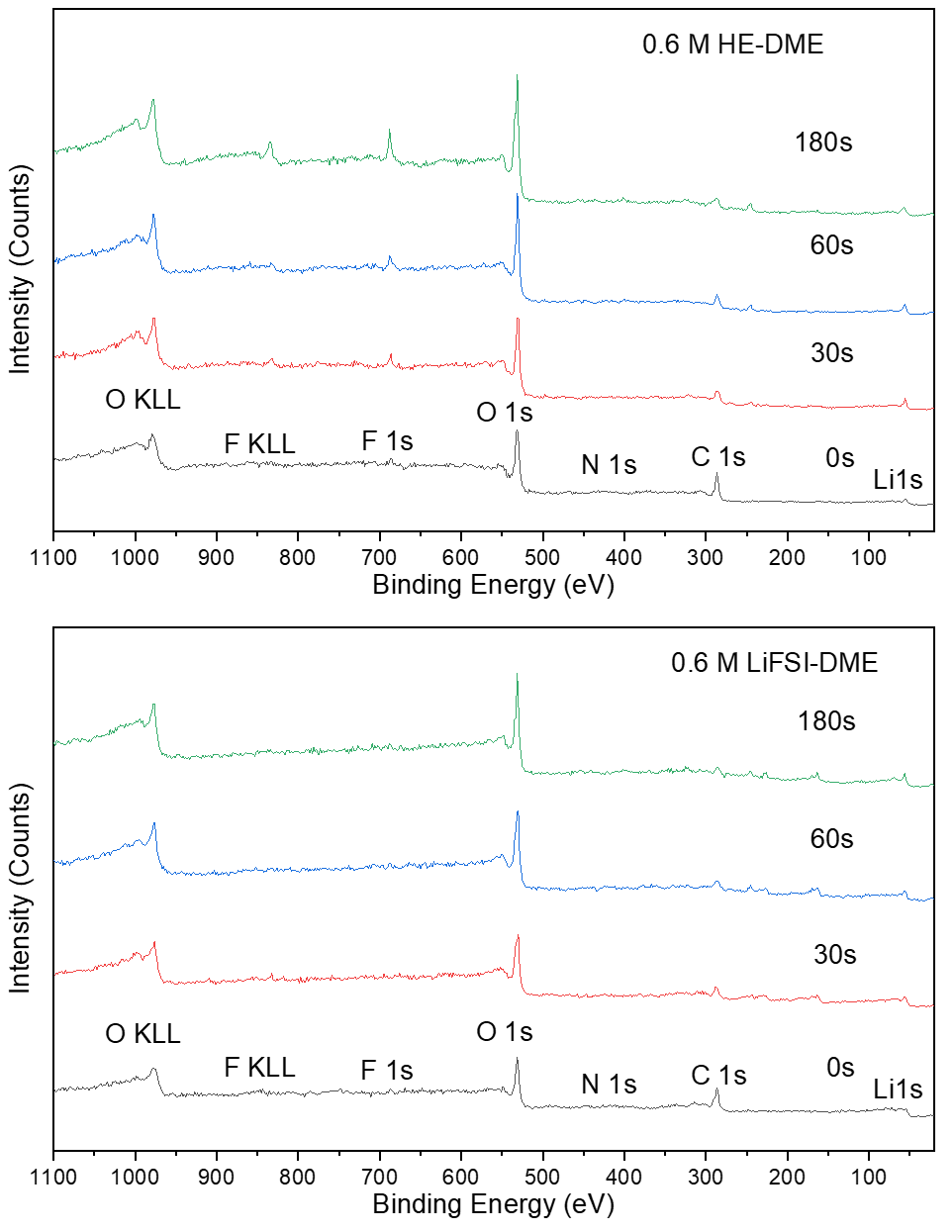


**Supplementary Fig. 51. Surveys of X-ray photoelectron spectroscopy (XPS) spectra of lithium metal electrodes in 0.6 M HE-DME and 0.6 M LiFSI-DME electrolytes.** Spectra were recorded after cycles from the surface of Cu electrodes at different depths.


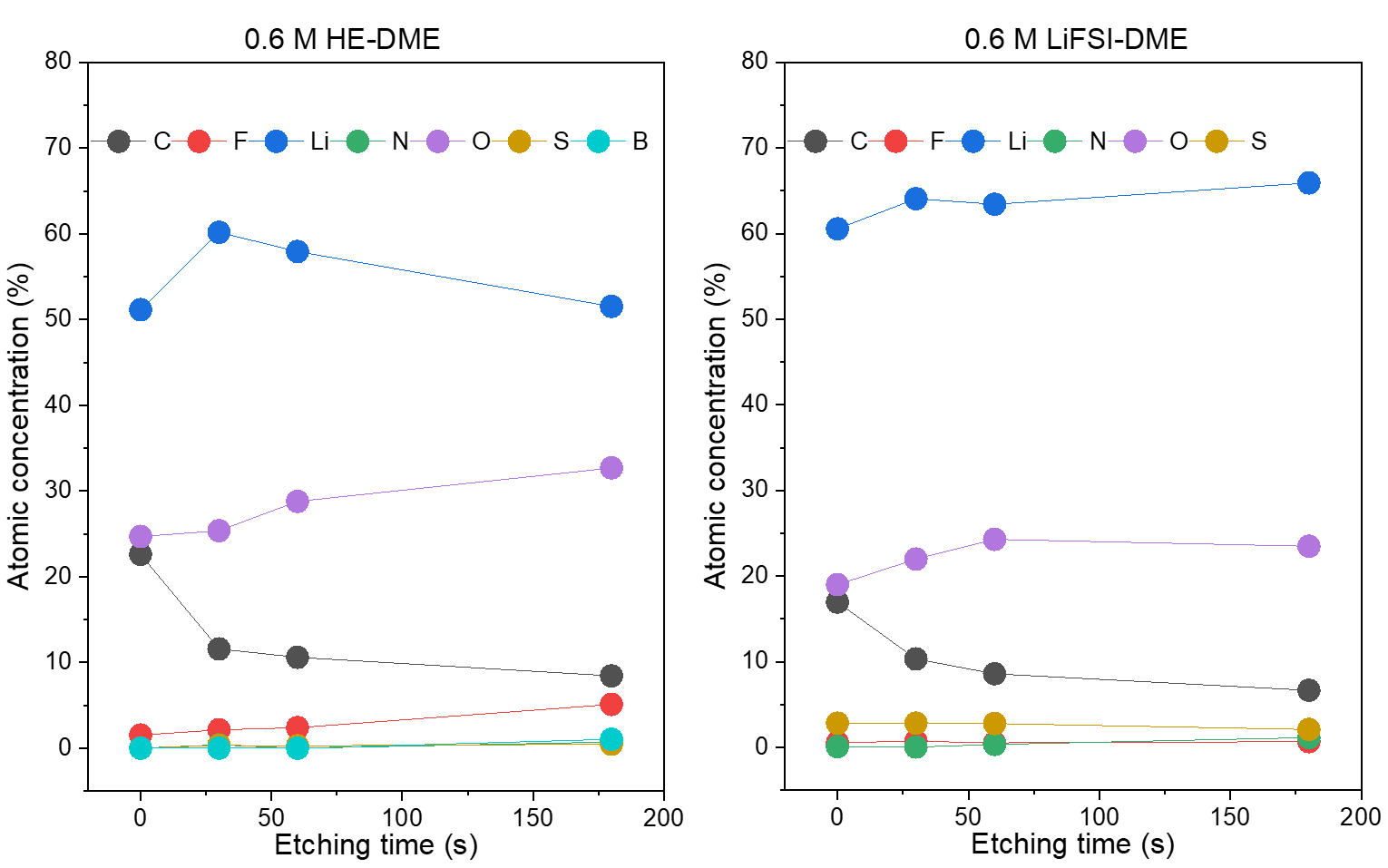


**Supplementary Fig. 52. Elemental composition on Cu electrodes from XPS spectra.** The left panel is from the 0.6 M HE-DME electrolyte and the right panel is from the 0.6 M LiFSI-DME electrolyte.


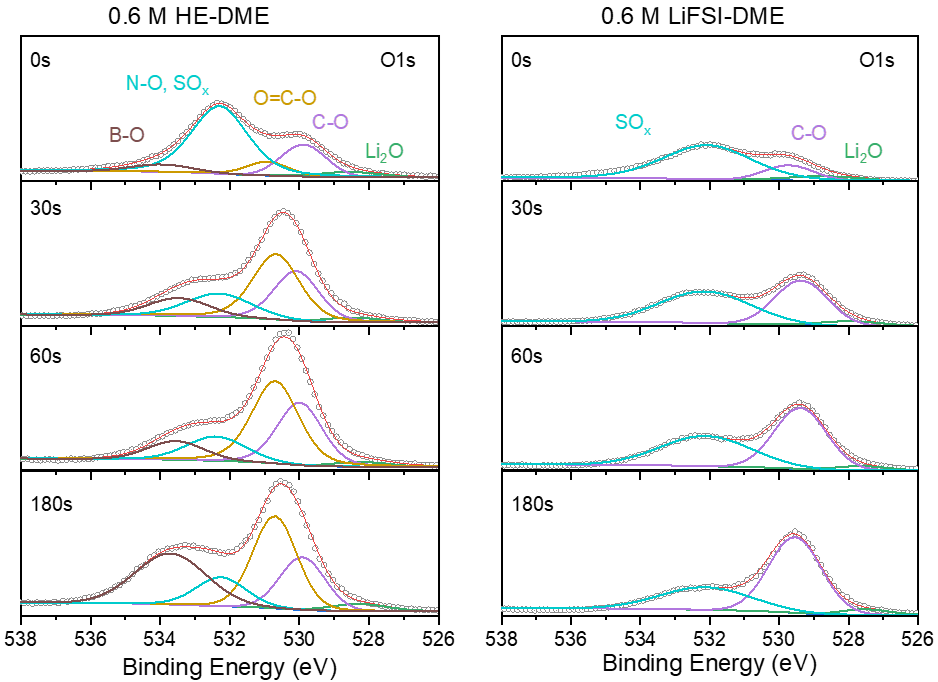


**Supplementary Fig. 53. O 1*s* spectra on the surface of the Cu electrodes.** The left panel is from the 0.6 M HE-DME electrolyte and the right panel is from the 0.6 M LiFSI-DME electrolyte.


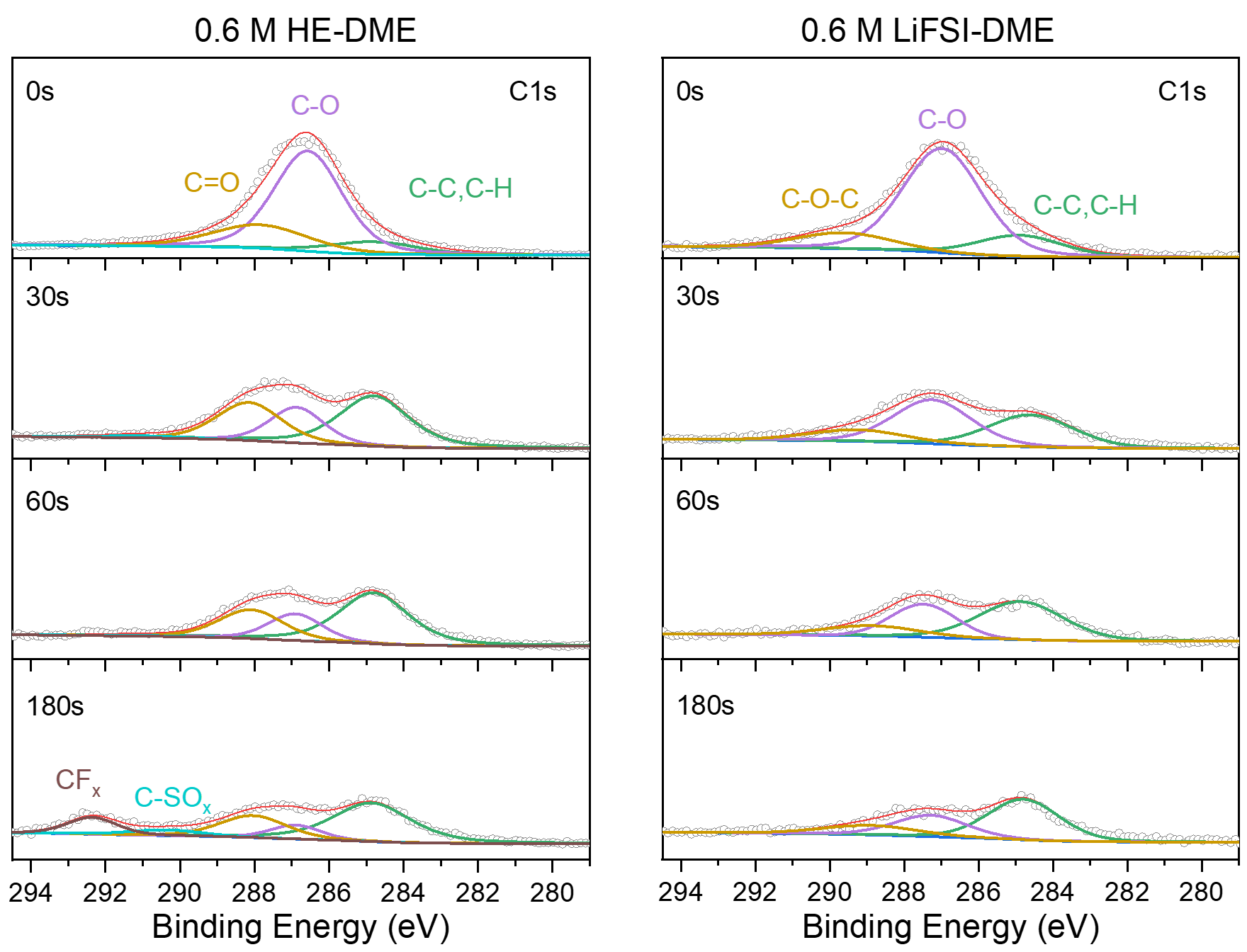


**Supplementary Fig. 54. C 1*s* spectra on the surface of the Cu electrodes.** The left panel is from the 0.6 M HE-DME electrolyte and the right panel is from the 0.6 M LiFSI-DME electrolyte.


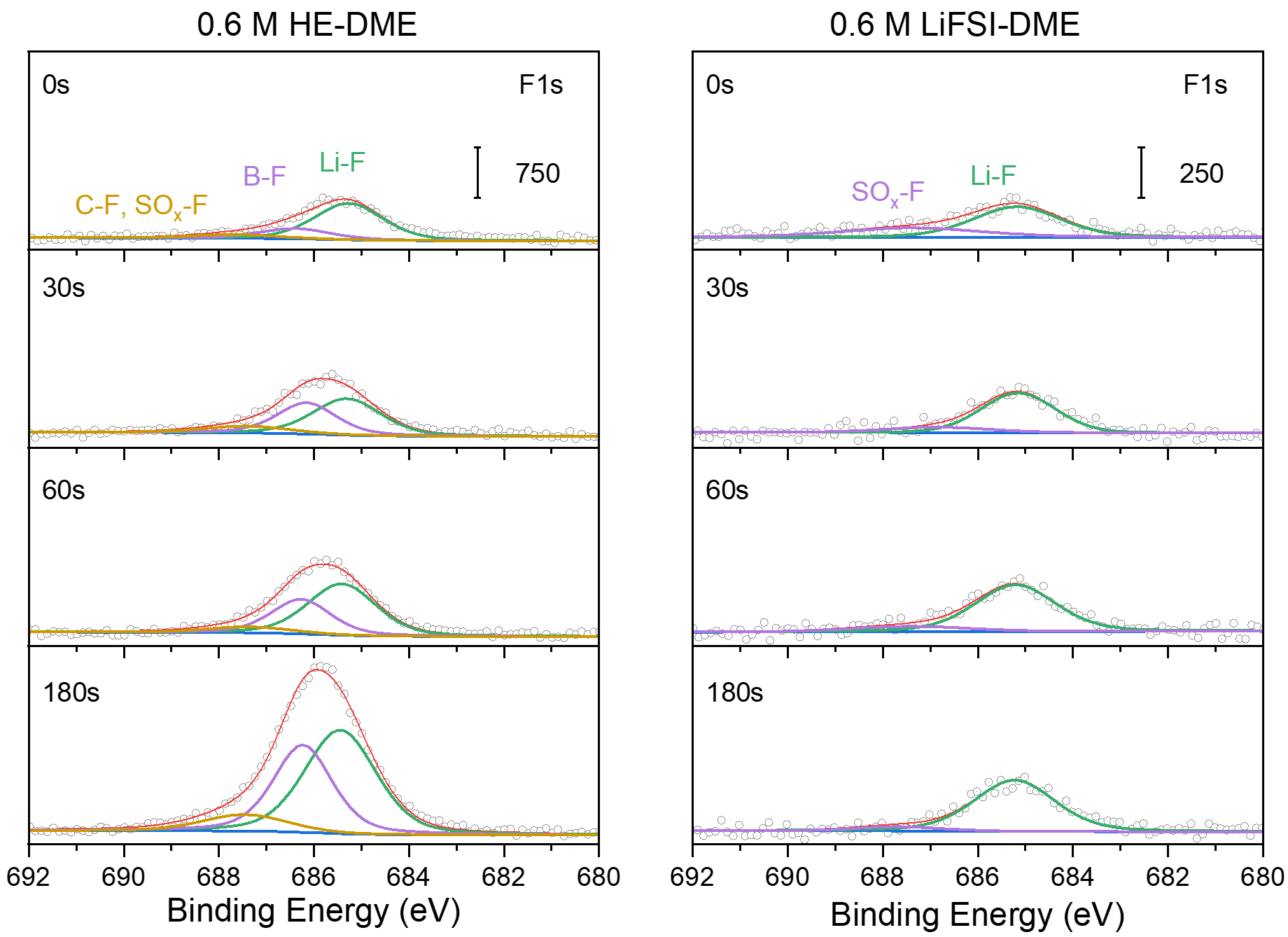


**Supplementary Fig. 55. F 1*s* spectra on the surface of the Cu electrodes.** The left panel is from the 0.6 M HE-DME electrolyte and the right panel is from the 0.6 M LiFSI-DME electrolyte.


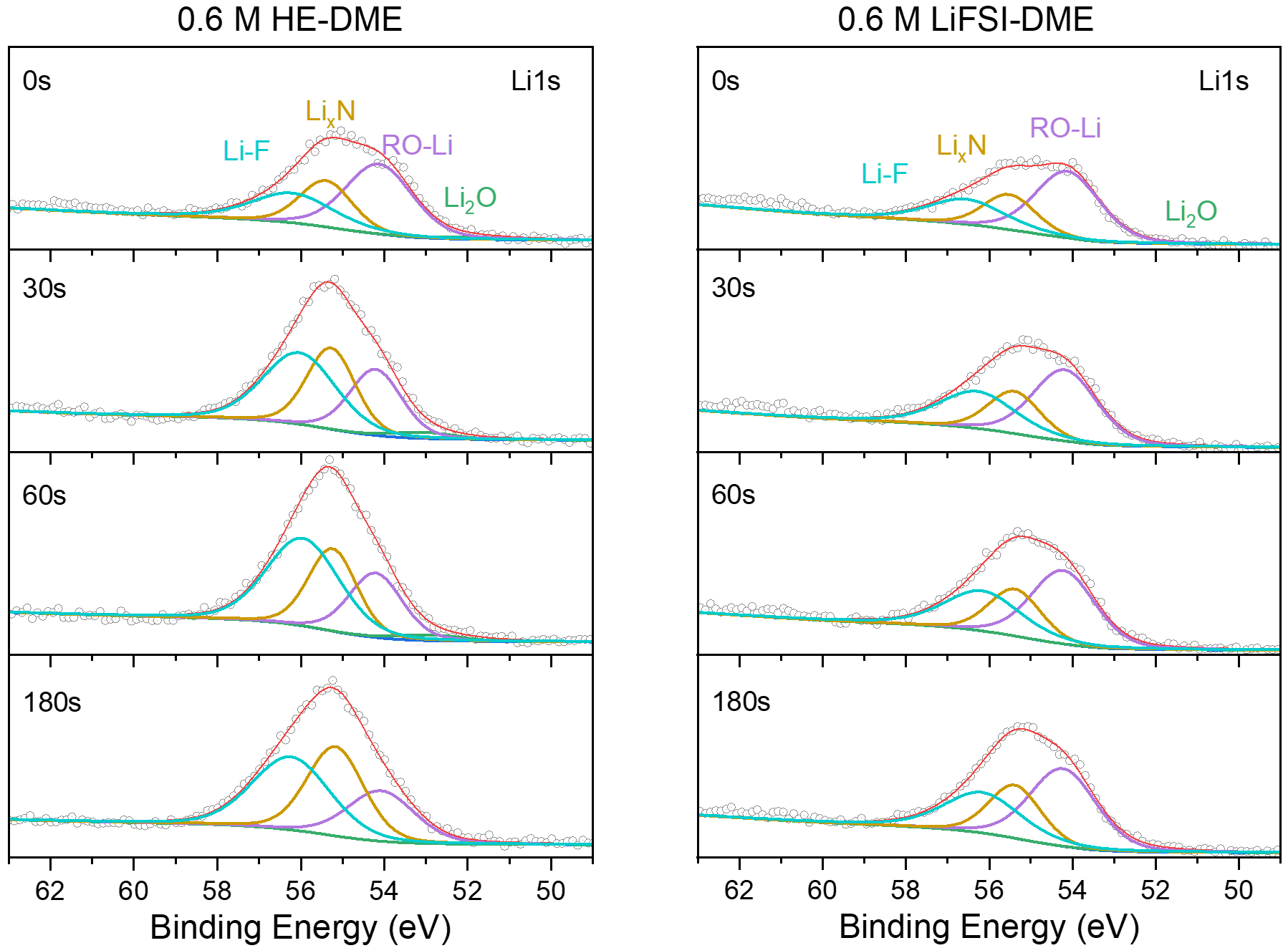


**Supplementary Fig. 56. Li 1*s* spectra on the surface of the Cu electrodes.** The left panel is from the 0.6 M HE-DME electrolyte and the right panel is from the 0.6 M LiFSI-DME electrolyte.


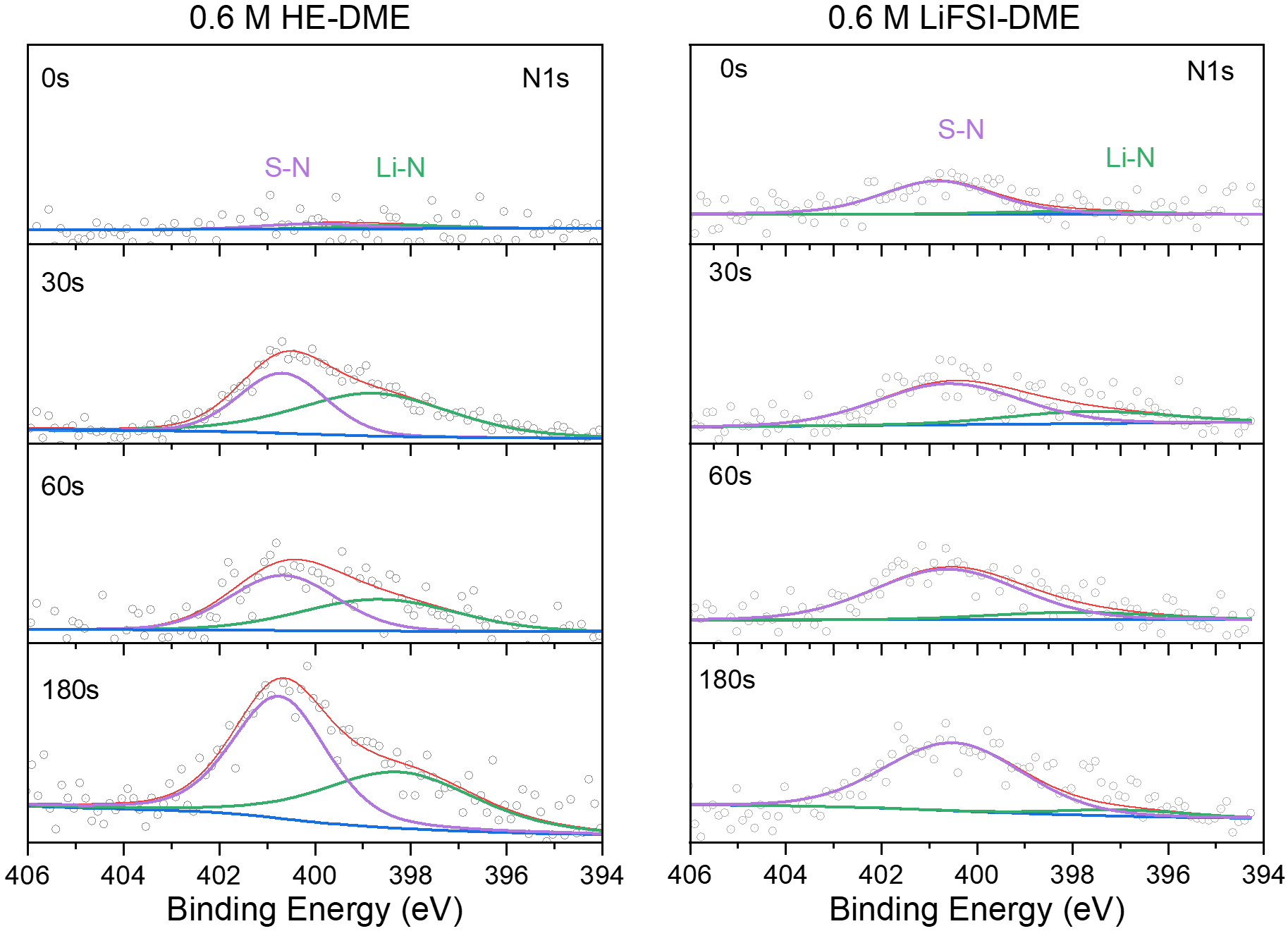


**Supplementary Fig. 57. N 1*s* spectra on the surface of the Cu electrodes.** The left panel is from the 0.6 M HE-DME electrolyte and the right panel is from the 0.6 M LiFSI-DME electrolyte.


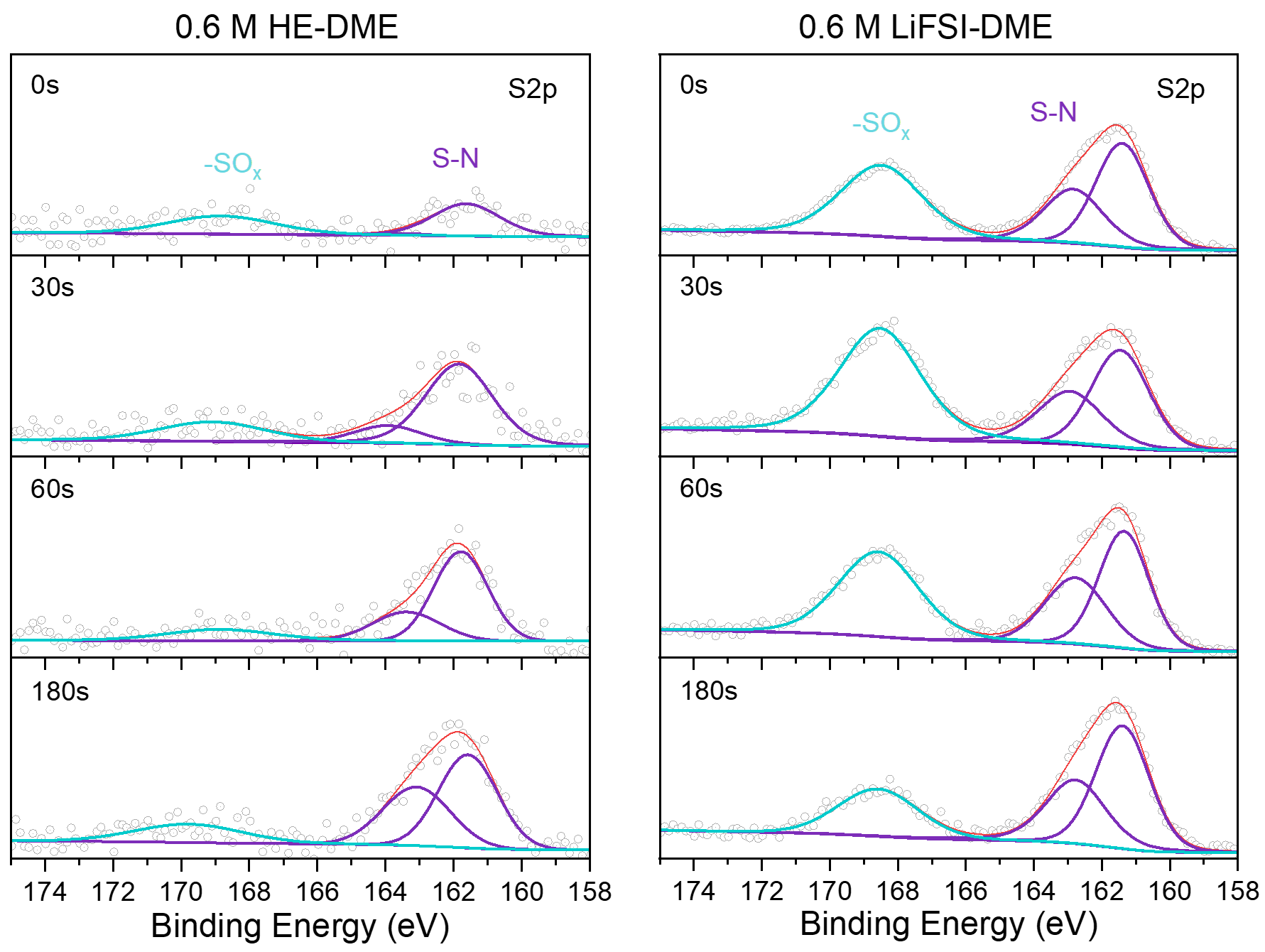


**Supplementary Fig. 58. S 2*p* spectra on the surface of the Cu electrodes.** The left panel is from the 0.6 M HE-DME electrolyte and the right panel is from the 0.6 M LiFSI-DME electrolyte.


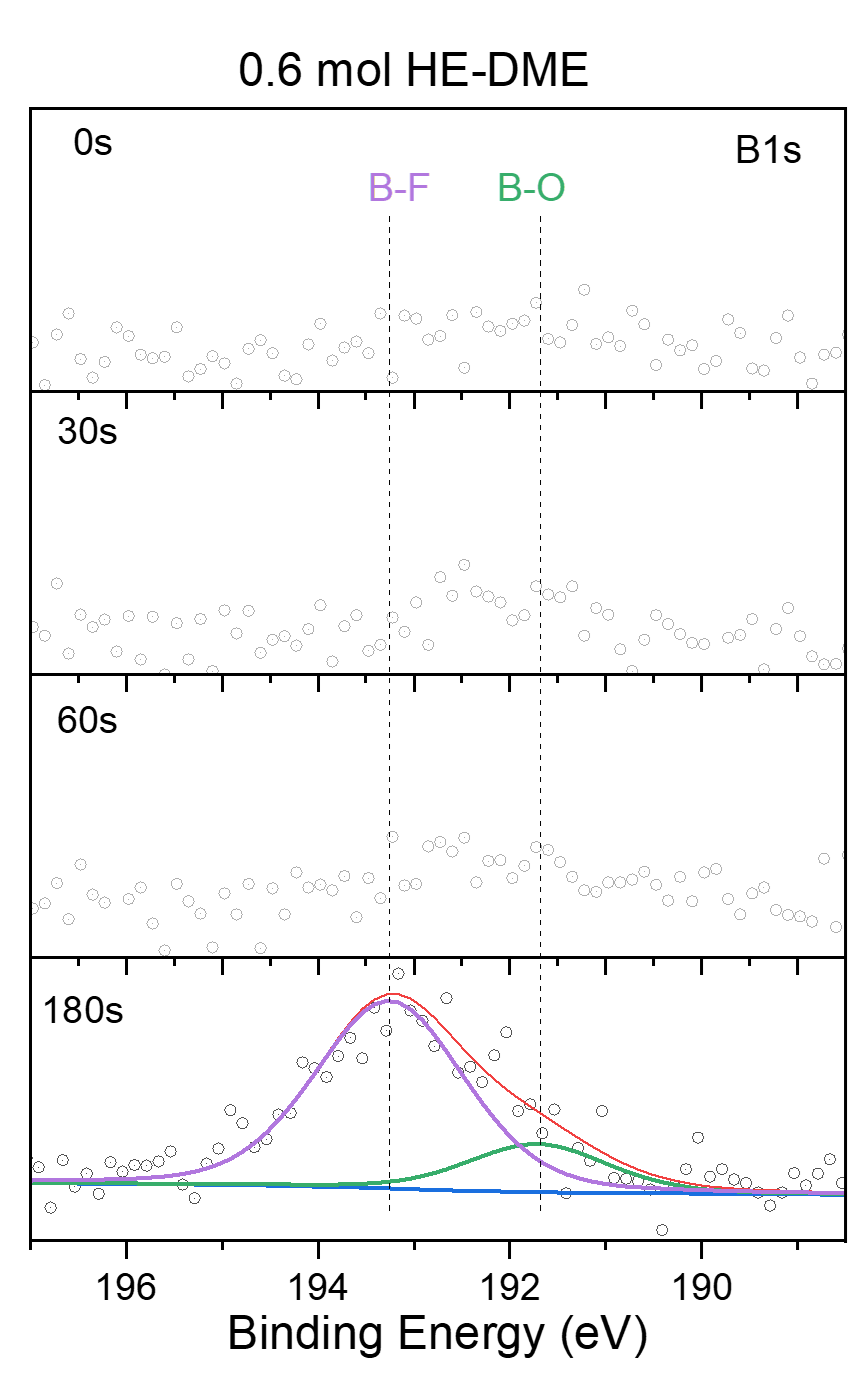


**Supplementary Fig. 59. B 1*s* spectra on the surface of the Cu electrode from the 0.6 M HE-DME electrolyte.**


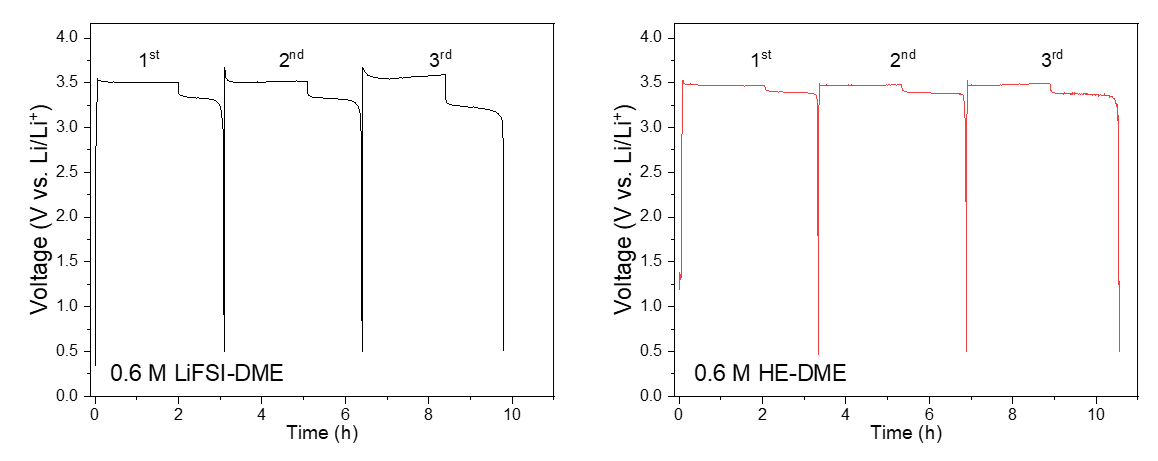


**Supplementary Fig. 60. Galvanostatic charge/discharge curves of Cu||LiFePO_4_ cells using 0.6 M LiFSI-DME and 0.6 M HE-DME electrolytes for the operando NMR tests.**


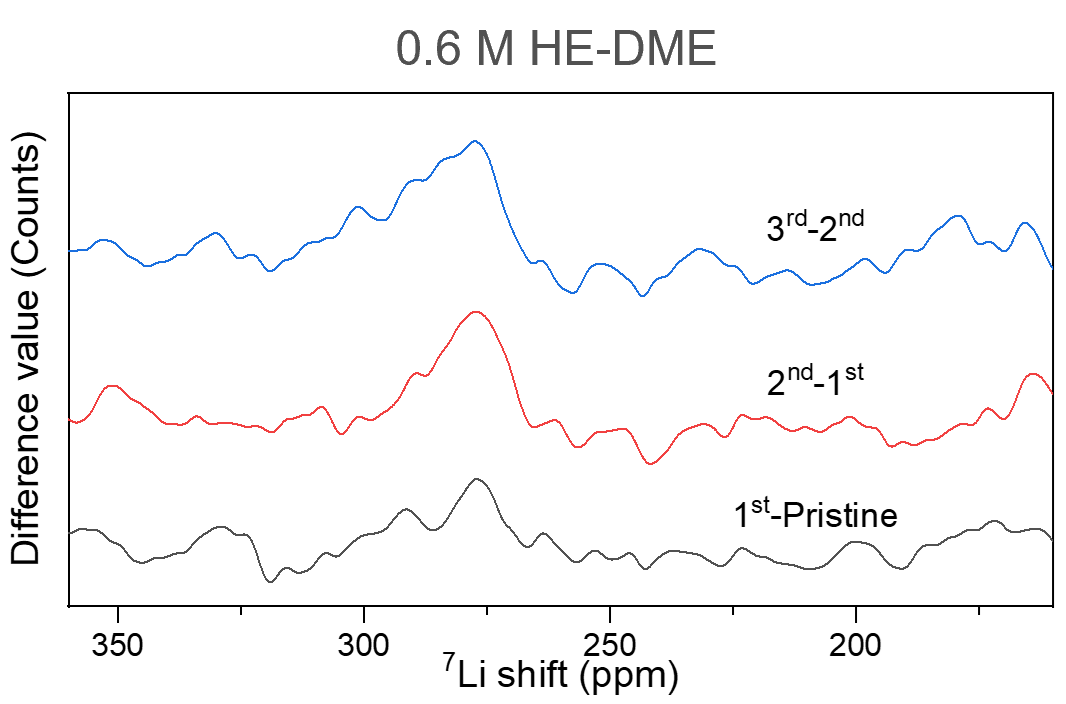


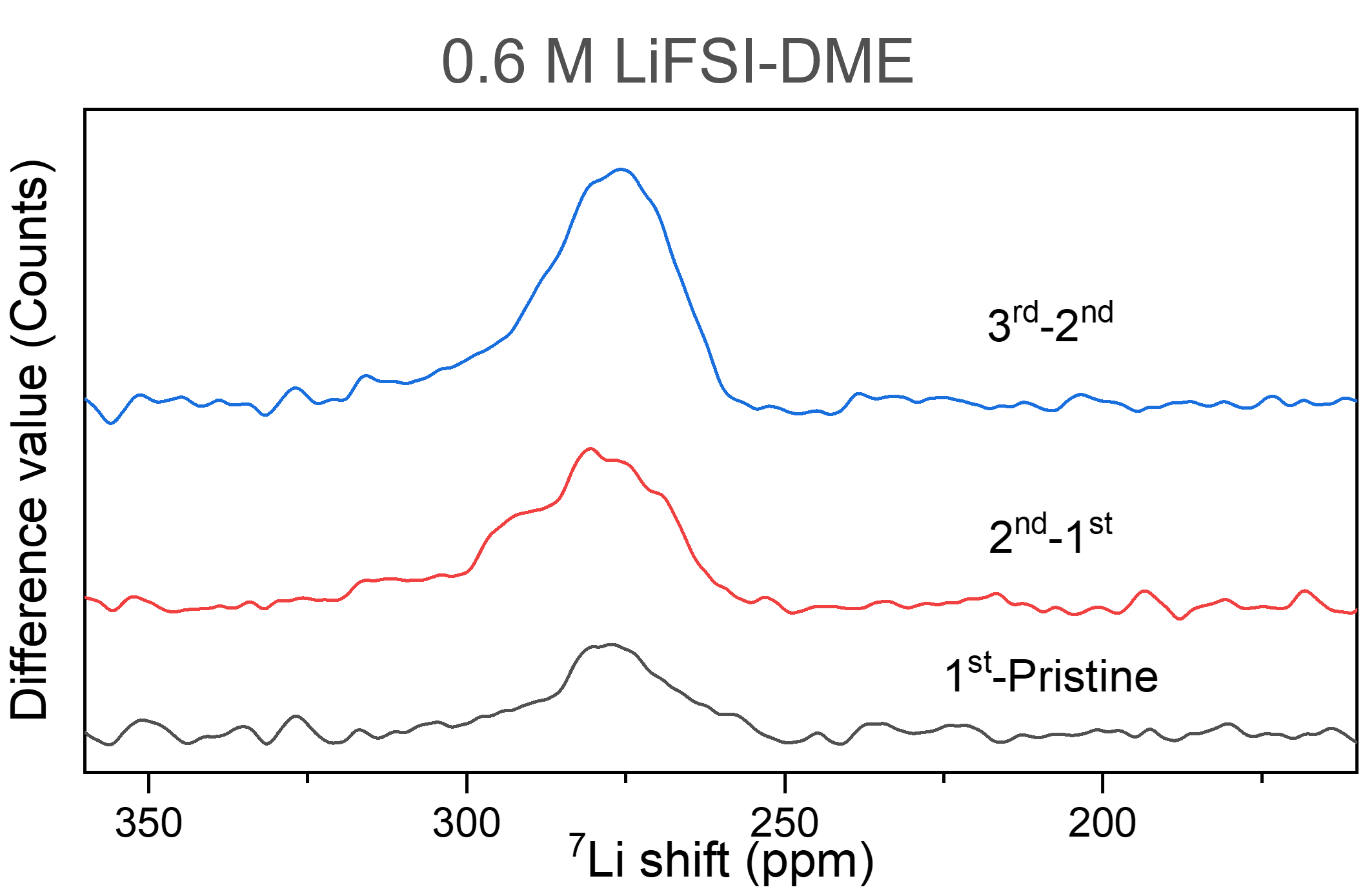


**Supplementary Fig. 61. ^7^Li solid-state NMR spectra after different cycles from 0.6 M HE-DME and 0.6 M LiFSI-DME electrolytes.** These one-dimensional spectra are the difference from the stripped states for the first, second and third cycles.


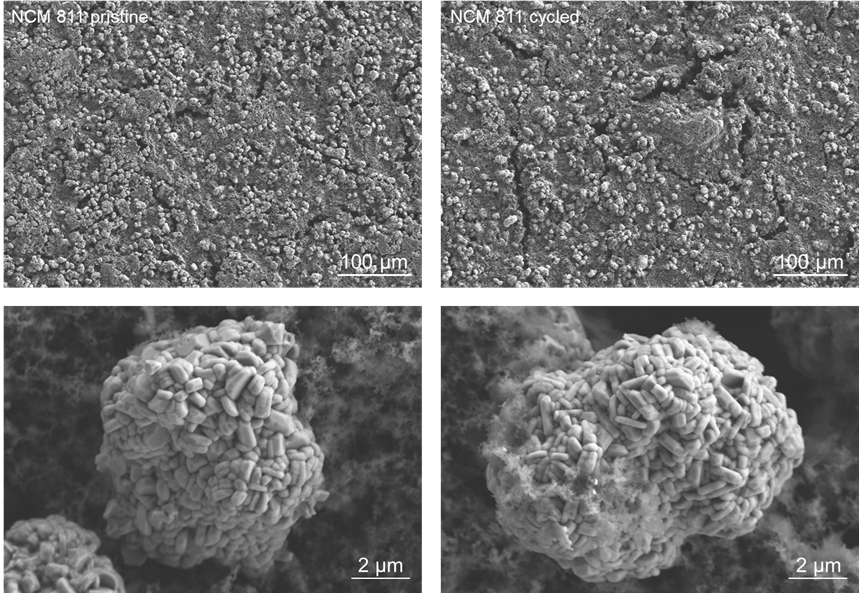


**Supplementary Fig. 62. SEM images of NCM811 cathode at different magnifications.** The left panels are the pristine electrodes before cycling, and the right panels are the cycled electrodes. It is shown that secondary micron sized particles both uncycled and cycled consisted of densely packed primary sub-micron sized particles with a clean surface.


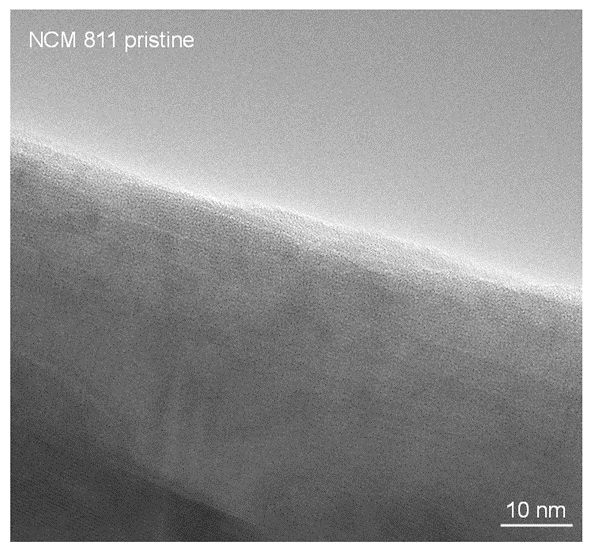


**Supplementary Fig. 63. TEM image of pristine NCM811 cathode.** No interphase layer could be observed along the surface of the particle.


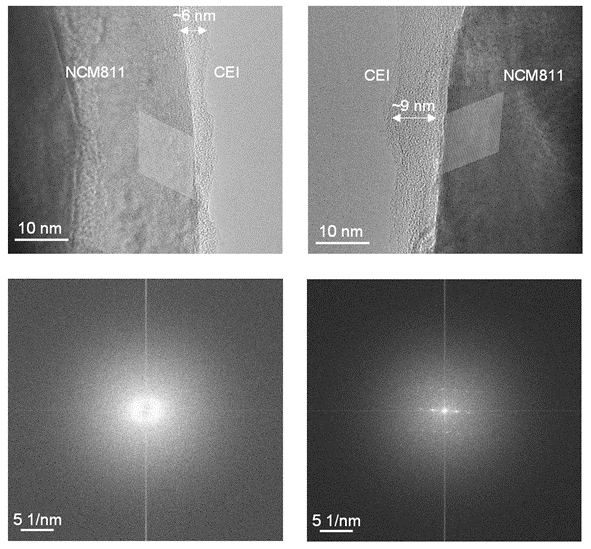


**Supplementary Fig. 64. Morphology, microstructure and composition of cathode electrolyte interphase.** Cryo-TEM images of the cathode electrolyte interphase (CEI) from different NCM811 cathode particles. A clear CEI layer was observed along the surface of the particle with the thickness between 6 and 9 nm. The bottom panels show the fast Fourier transform images from the CEI area and the bulk area of the NCM811 cathode.


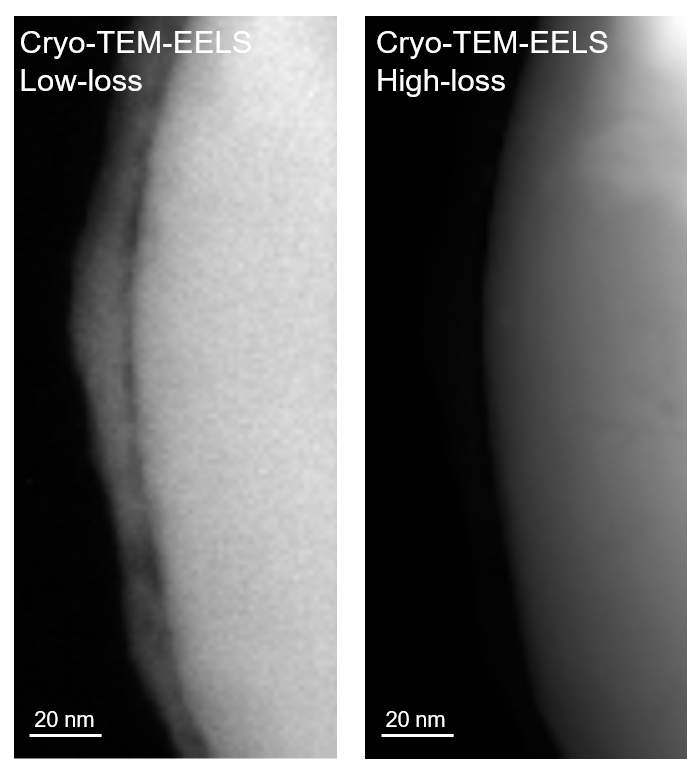


**Supplementary Fig. 65. Cryo-STEM EELS images of NCM811 cathode after 50 cycles.** The left panel is from the low-loss area with high recognition of light elements and the right panel is from the high-loss area.


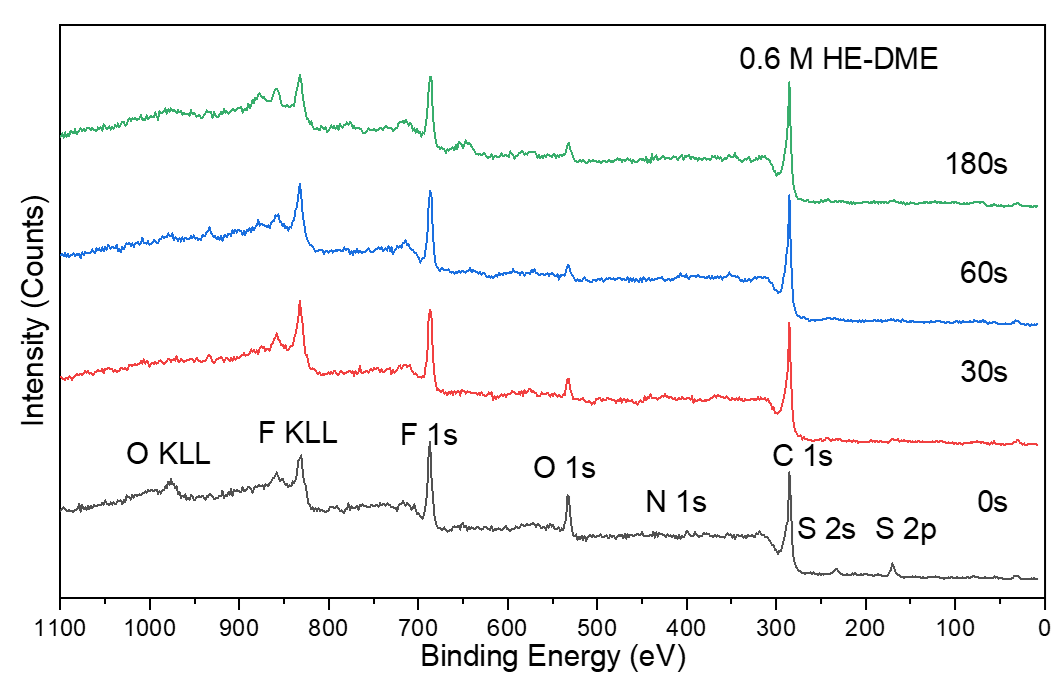


**Supplementary Fig. 66. Surveys of XPS spectra of the NCM811 electrode in the 0.6 M HE-DME electrolyte.** Spectra were recorded at different depths.


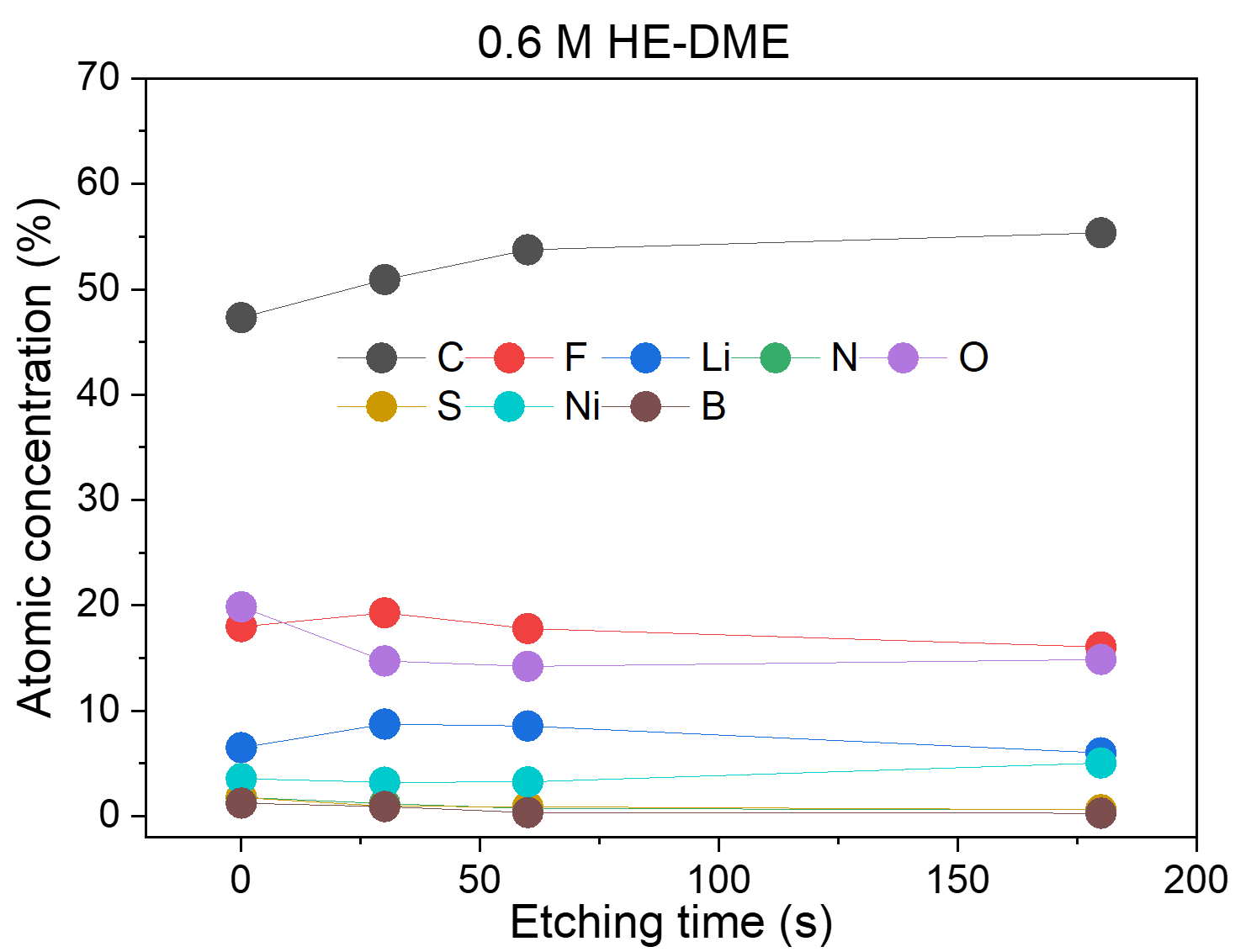


**Supplementary Fig. 67. Elemental composition on the surface of the NCM811 electrode from XPS spectra.**


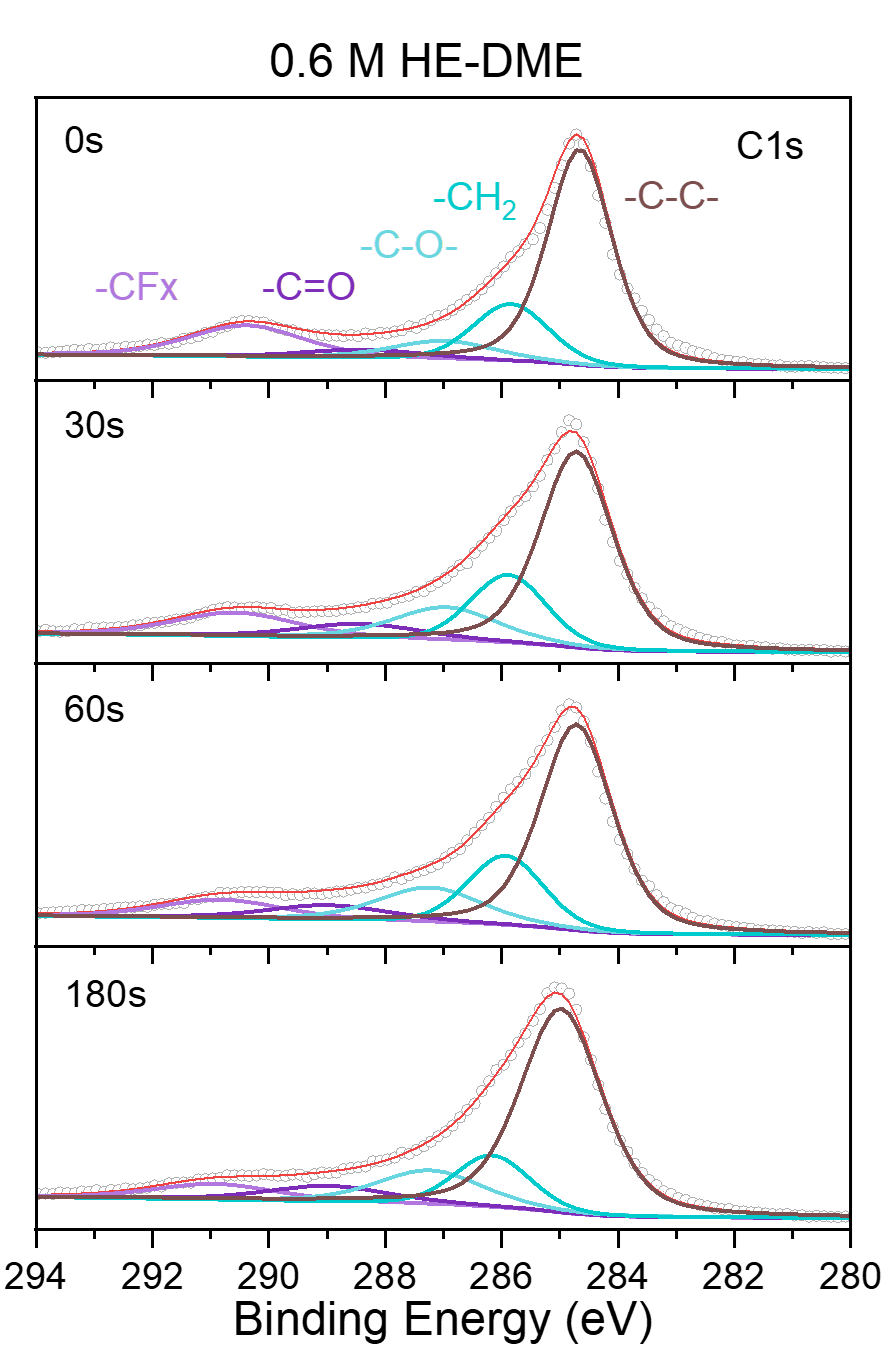


**Supplementary Fig. 68. C 1*s* spectra on the surface of the NCM811 cathode in the 0.6 M HE-DME electrolyte.**


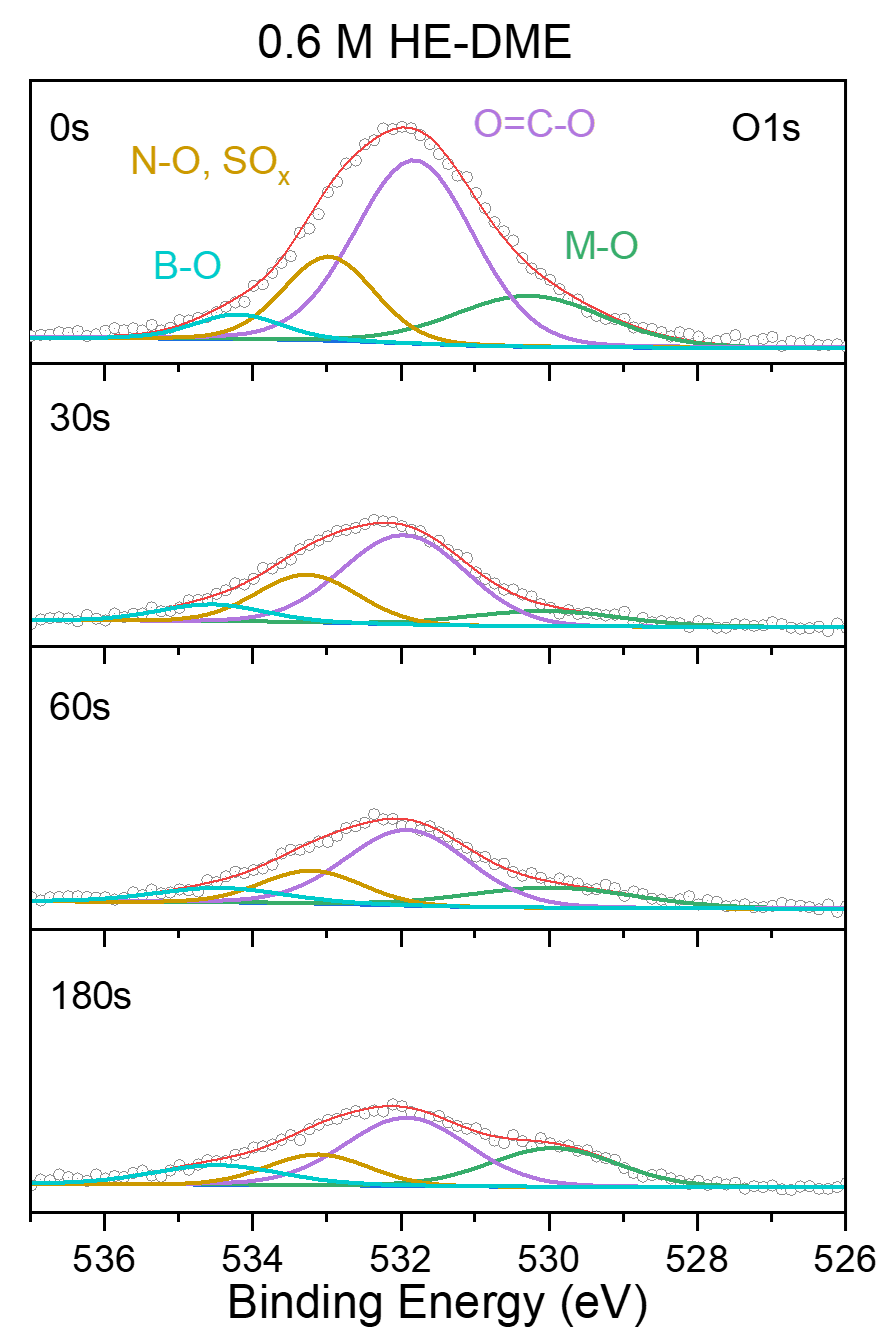


**Supplementary Fig. 69. O 1*s* spectra on the surface of the NCM811 cathode in the 0.6 M HE-DME electrolyte.**


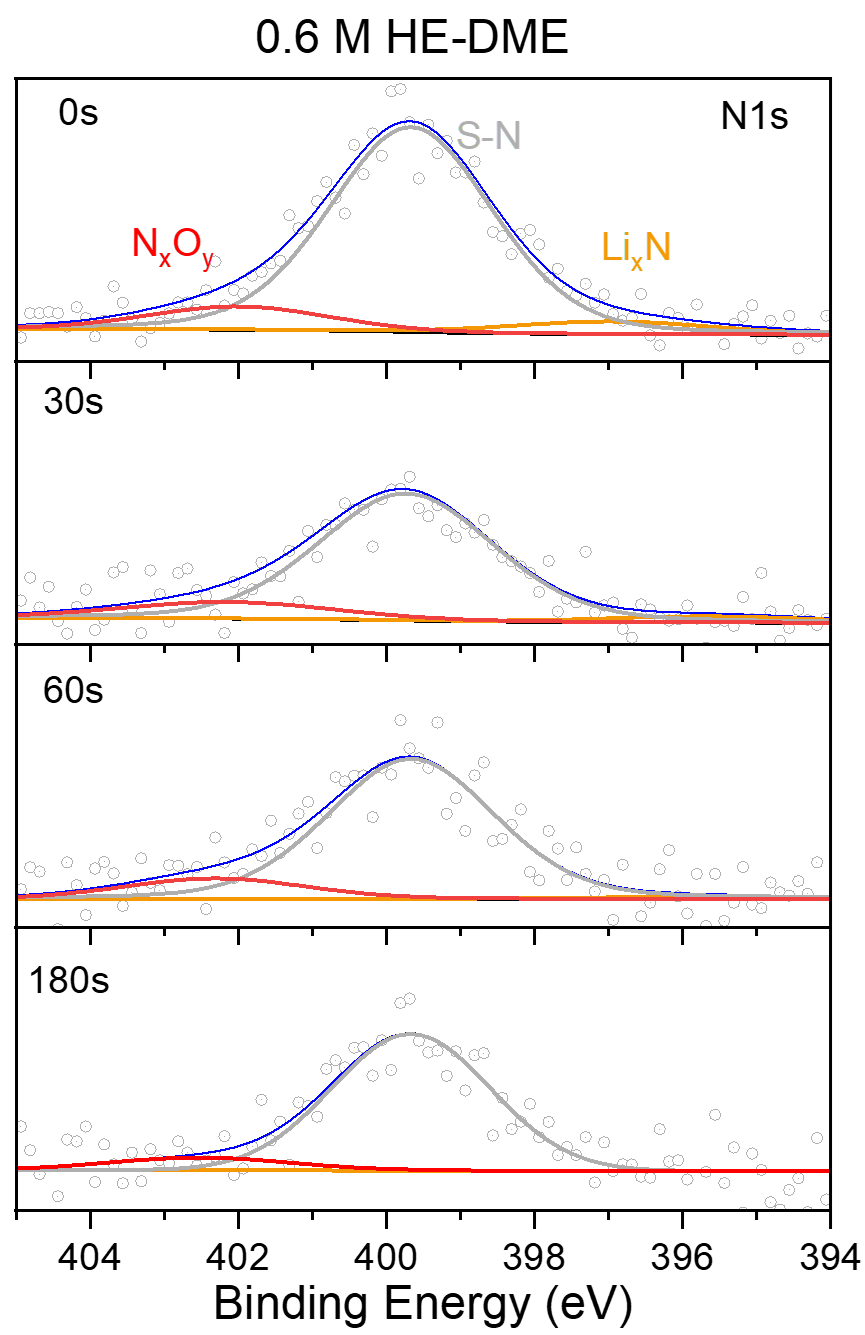


**Supplementary Fig. 70. N 1*s* spectra on the surface of the NCM811 cathode in the 0.6 M HE-DME electrolyte.**


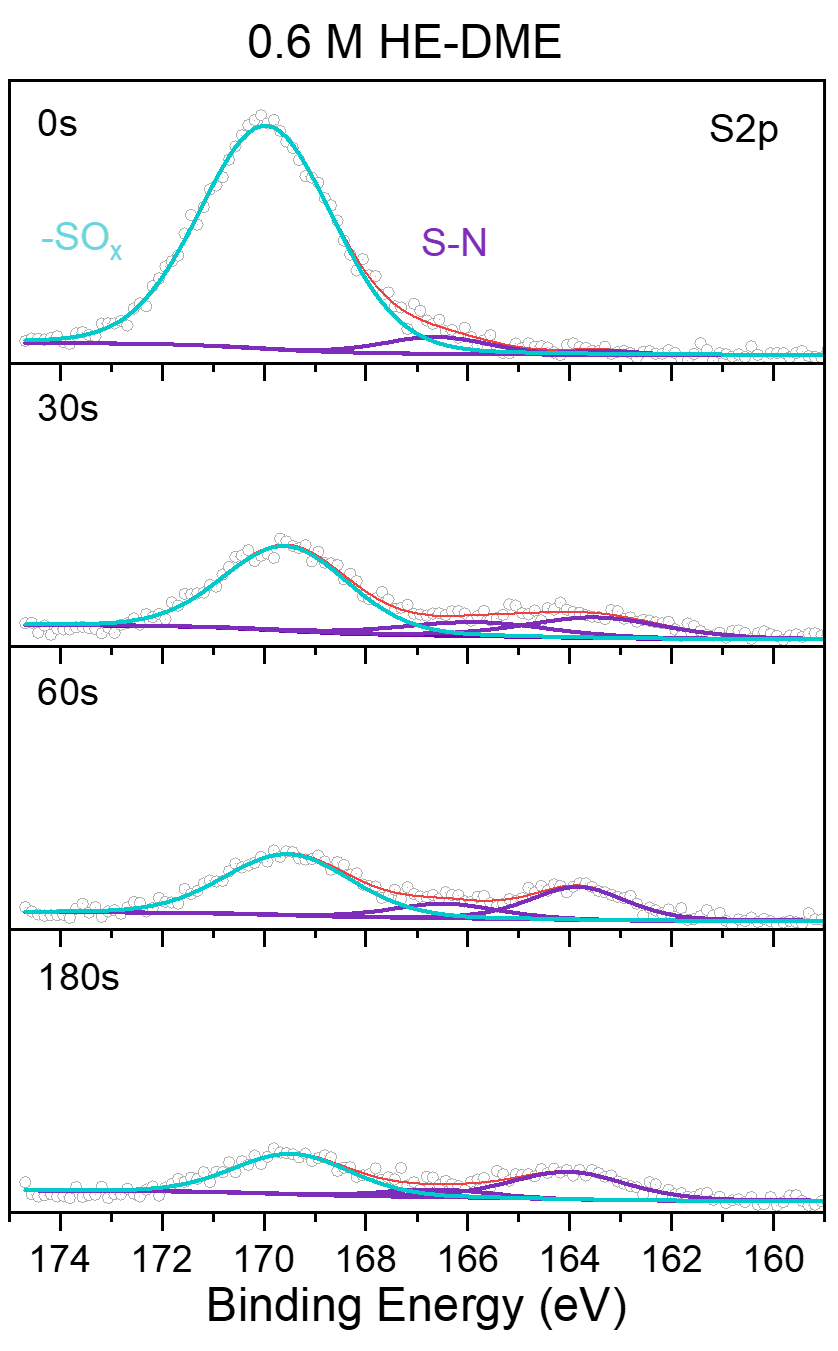


**Supplementary Fig. 71. S 2*p* spectra on the surface of the NCM811 cathode in the 0.6 M HE-DME electrolyte.**


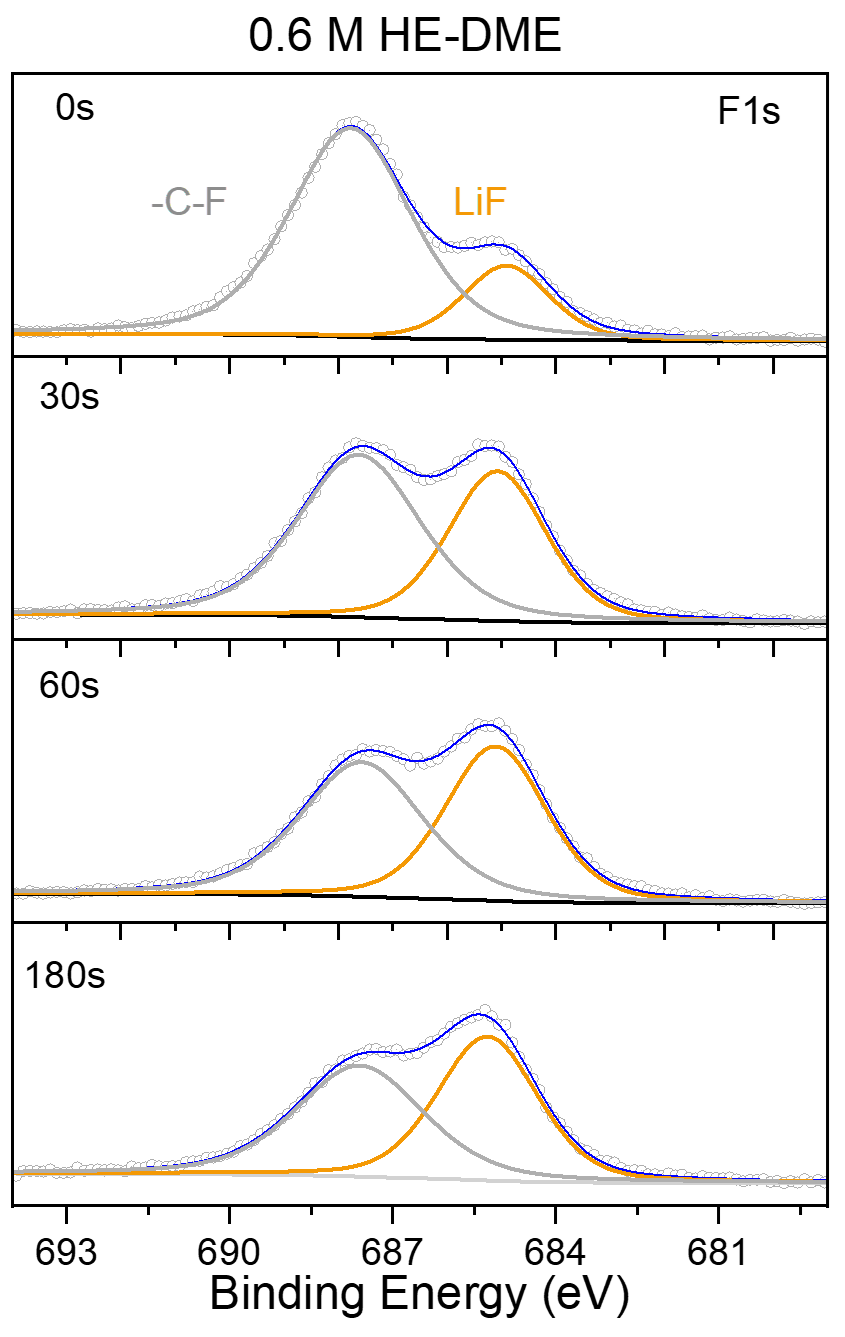


**Supplementary Fig. 72. F 1*s* spectra on the surface of the NCM811 cathode in the 0.6 M HE-DME electrolyte.**


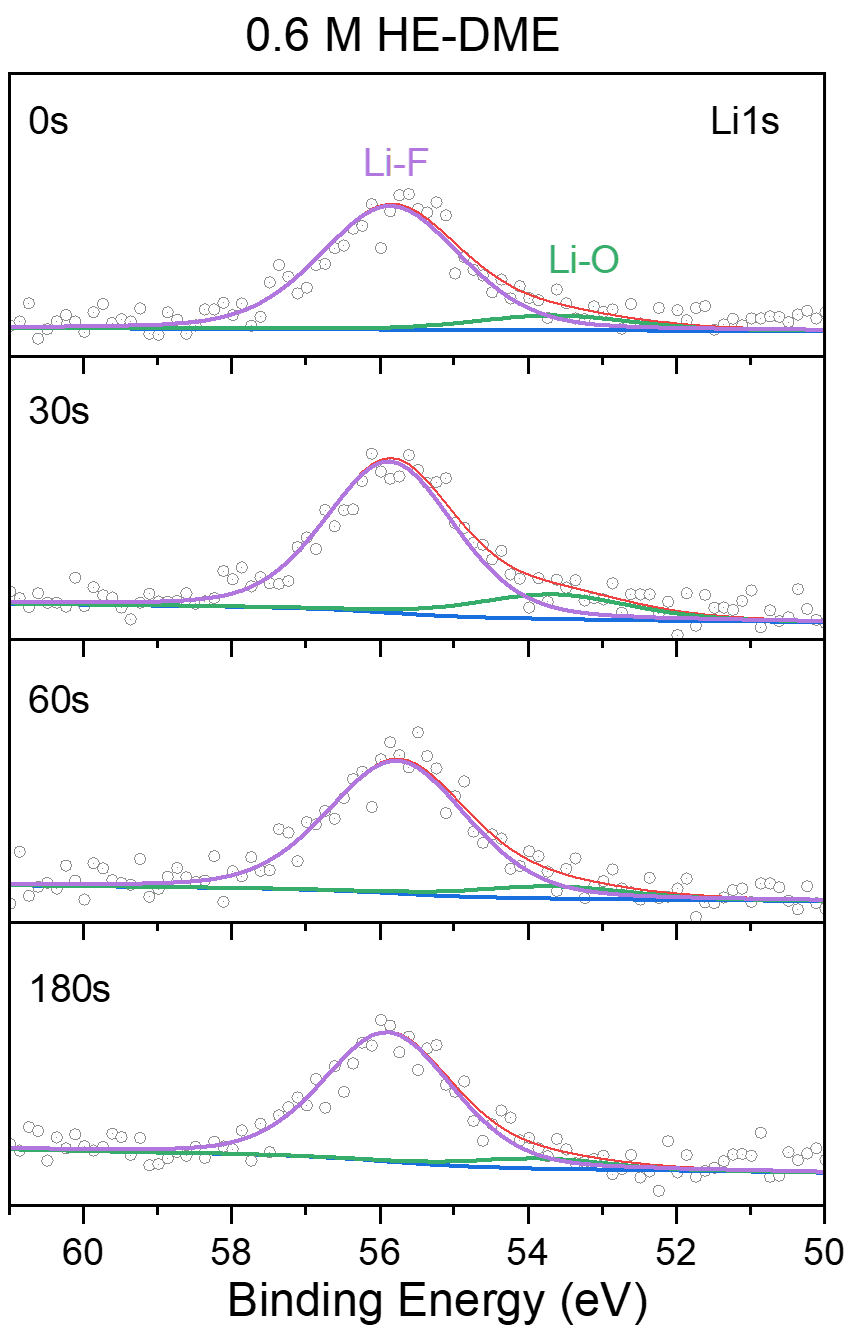


**Supplementary Fig. 73. Li 1*s* spectra on the surface of the NCM811 cathode in the 0.6 M HE-DME electrolyte.**


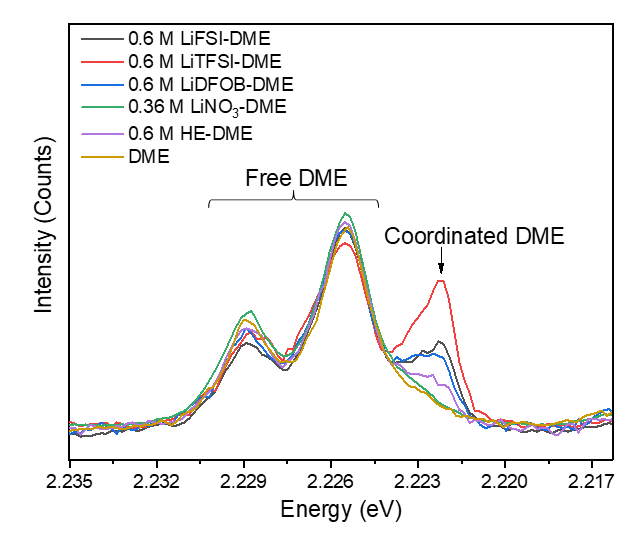


**Supplementary Fig. 74. Solvation structure from Raman spectra of 0.6 M HE-DME electrolyte** **and 0.6 M LiFSI-DME, 0.6 M LiTFSI-DME, 0.6 M LiDFOB-DME and 0.36 M LiNO_3_-DME electrolytes.**


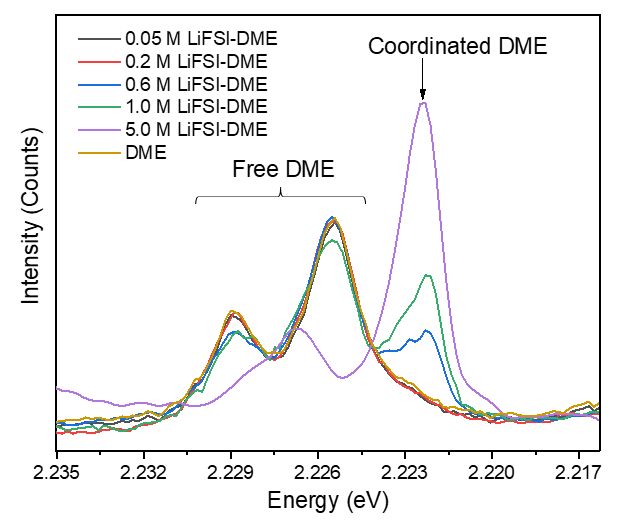


**Supplementary Fig. 75. Solvation structure from Raman spectra of 0.05 M, 0.2 M, 0.6 M, 1.0 M and 5.0 M LiFSI-DME electrolytes.**


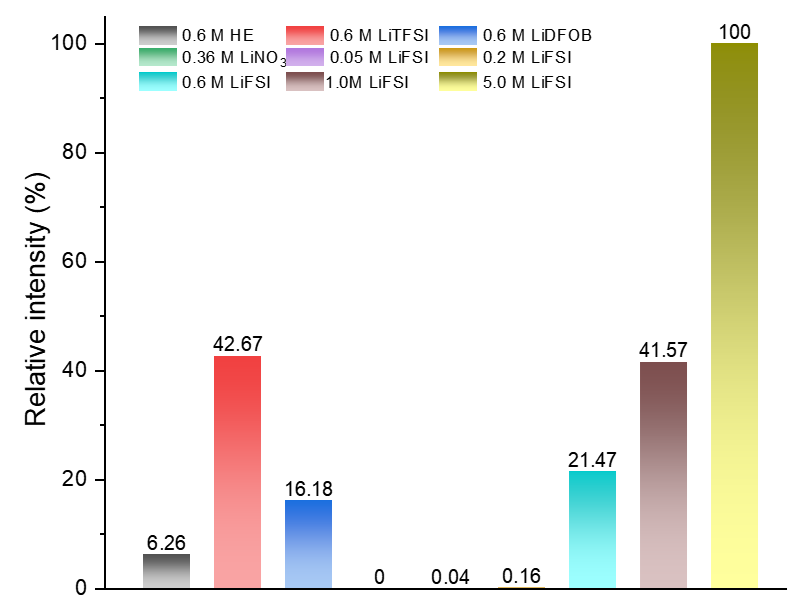


**Supplementary Fig. 76. Solvation structure from Raman spectra of different electrolytes.** Compared with the other electrolytes, the 0.6 M HE-DME electrolyte shows relatively weak DME solvent coordination (the intensity of the peak at 2.23 eV), very similar to the lower salt concentration 0.05 M and 0.2 M LiFSI-DME electrolytes, which can be related to various solvation structures co-existing in this HE-DME electrolyte.


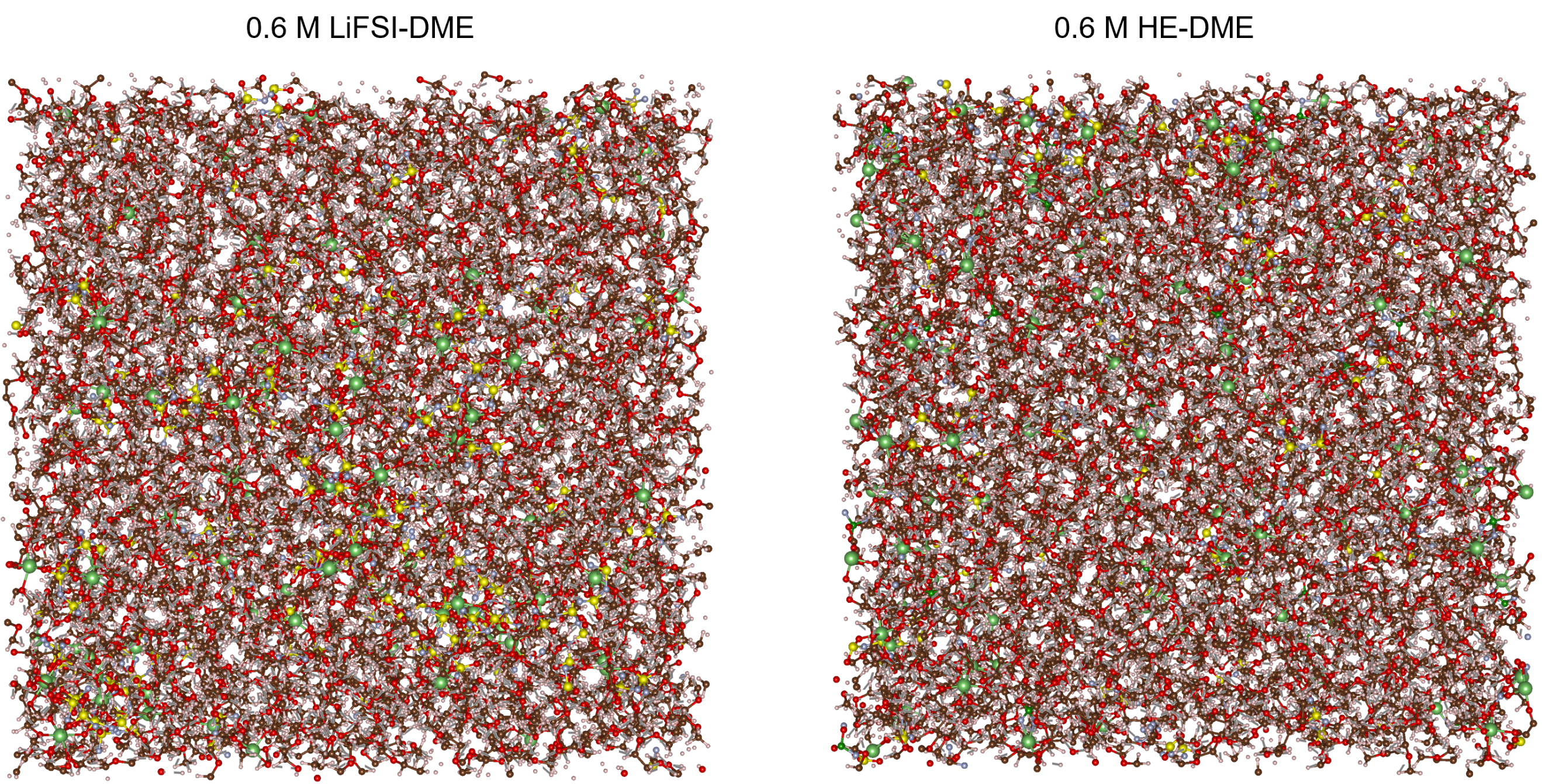


**Supplementary Fig. 77. Simulation structure for 0.6 M LiFSI-DME and 0.6 M HE-DME electrolytes.** See the method for details.


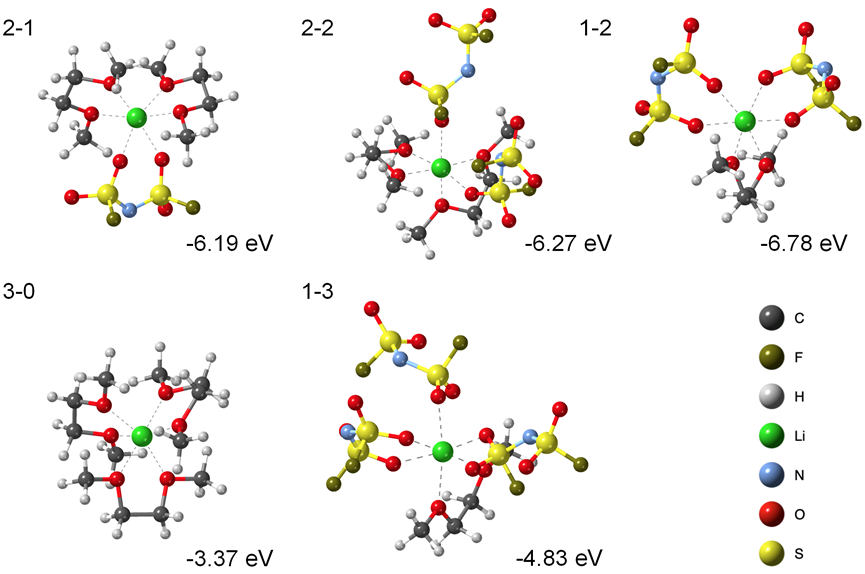


**Supplementary Fig. 78. The representative solvation structures and energies in the 0.6 M LiFSI-DME electrolyte.** The detailed structures are shown in supplementary table 2.


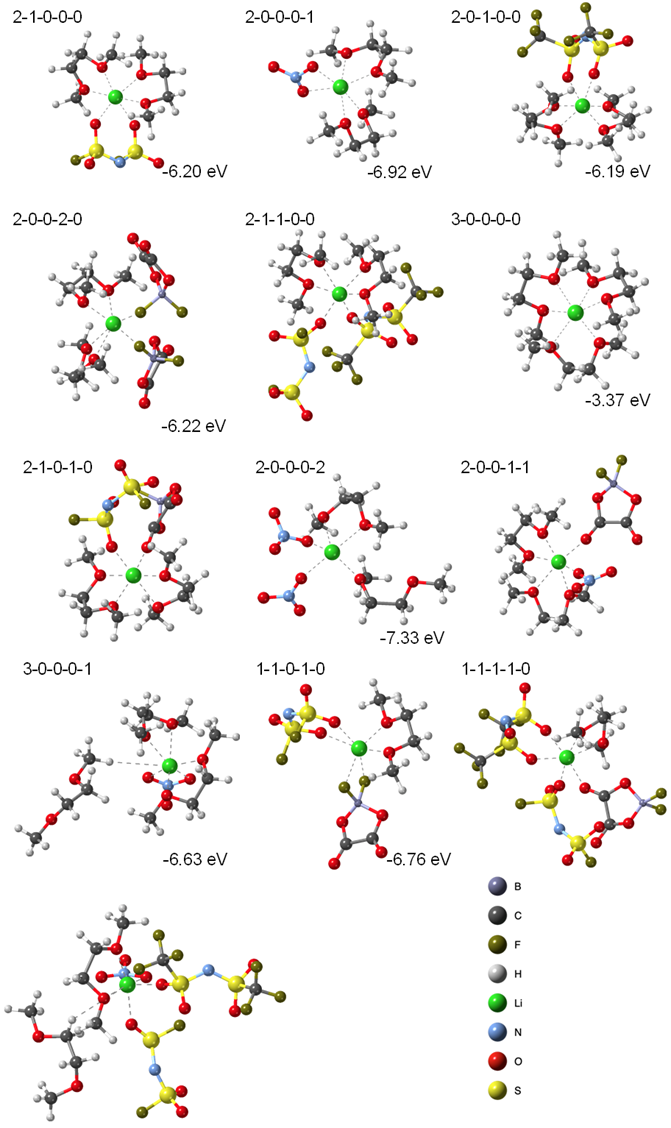


**Supplementary Fig. 79. The representative solvation structures and energies in the 0.6 M HE-DME electrolyte.** The detailed structures are shown in supplementary table 3.


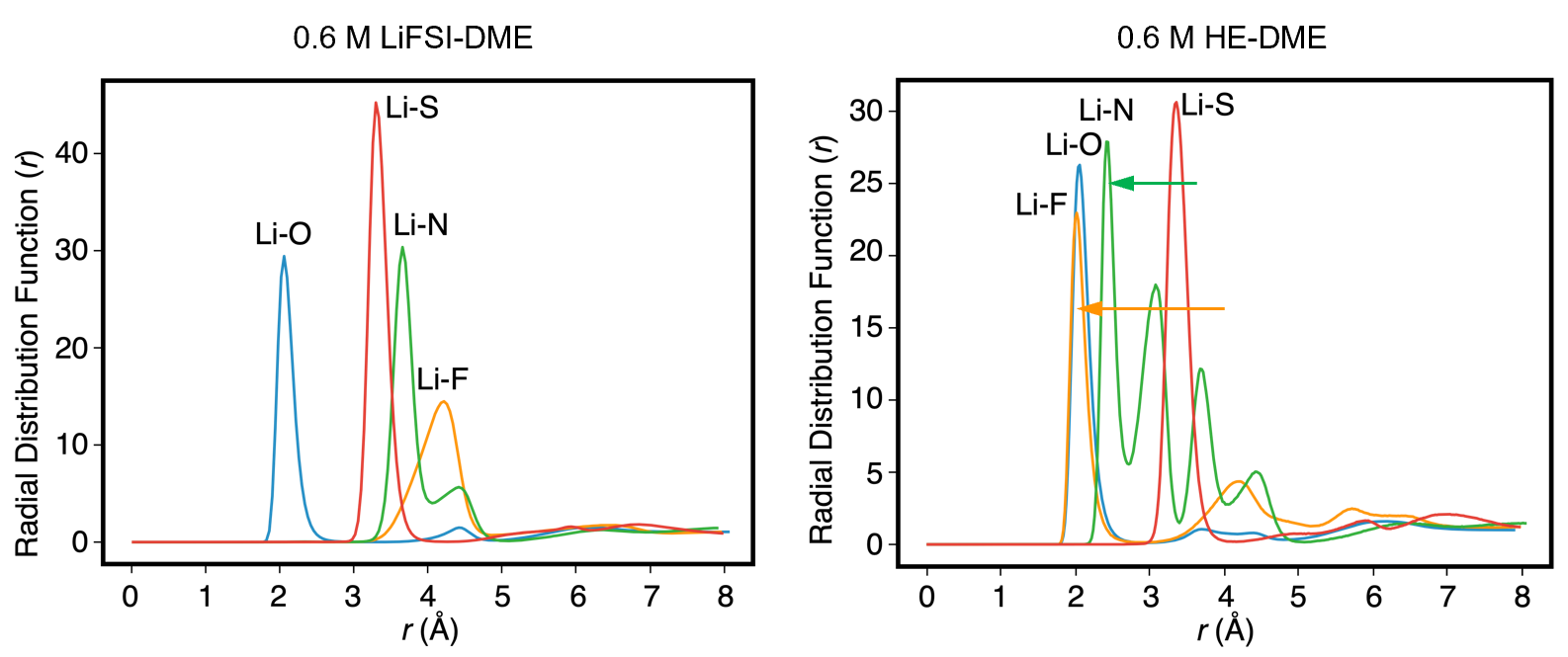


**Supplementary Fig. 80. Simulation of the radial distribution function (RDF) of Li–O, Li–F, Li–N and Li–S in 0.6 M LiFSI-DME and 0.6 M HE-DME electrolytes, respectively.** According to the RDF results of the 0.6 M LiFSI-DME electrolyte obtained from the MD simulations, oxygen shows a strong tendency to coordinate with the lithium-ion in comparison to the other elements, indicating a relatively strong interaction between lithium ions and solvent molecules. However, in the HE-DME electrolyte, fluorine and nitrogen also coordinate with lithium ions, indicating more anion rich solvation structures. This rationalizes the observation that for the HE electrolyte, both the SEI on anode and the CEI on the cathode are rich in decomposed salt anions.


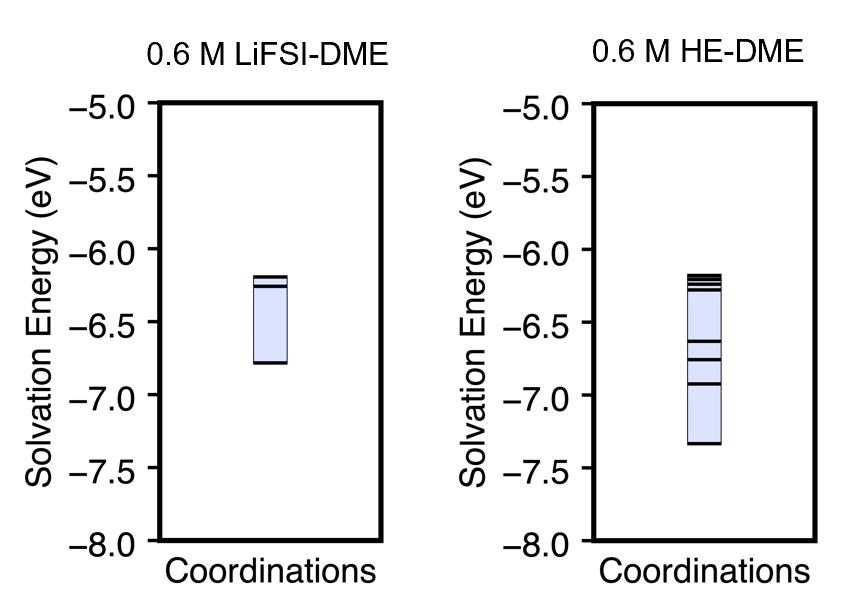


**Supplementary Fig. 81. The energies of the representative solvation structures in 0.6 M LiFSI-DME and 0.6 M HE-DME electrolytes.** The detailed structures are shown in supplementary table 3.


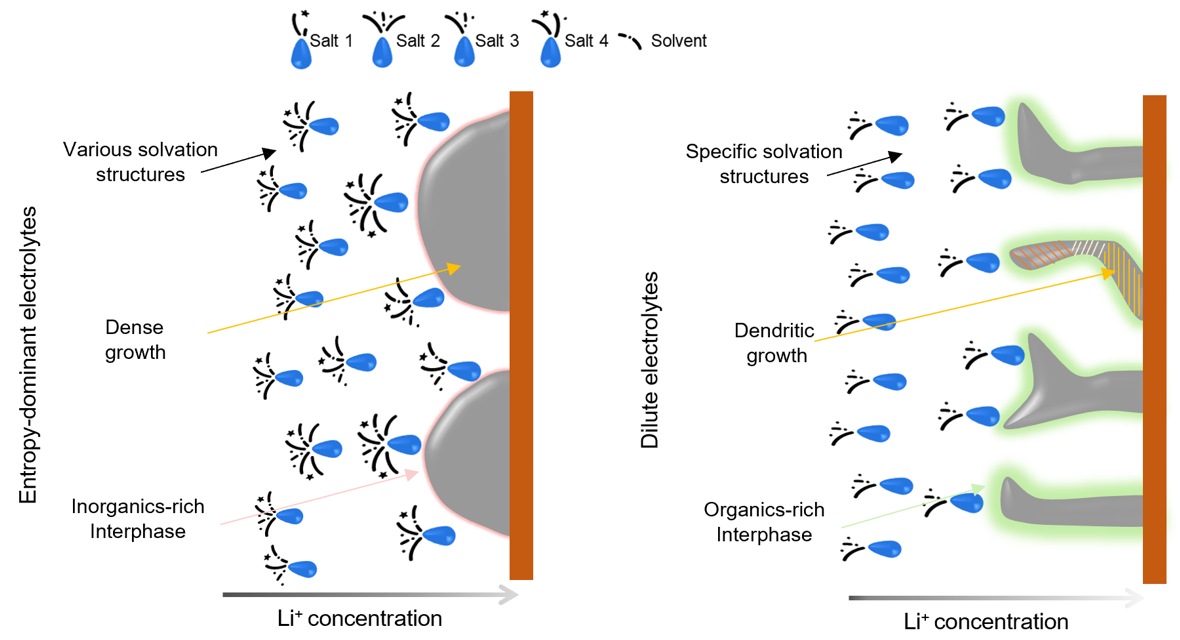


**Supplementary Fig. 82. The illustration of de-solvation processes in entropy-dominated electrolytes and conventional dilute electrolytes.** This diversity results in lower solvation reorganization energies that facilitate lithium-ion diffusion as well as charge transfer towards the interphase. The de-solvation processes in the entropy-dominated and conventional dilute electrolyte are further illustrated where in the HE electrolyte, the inorganic rich SEI/CEI and improved lithium-ion kinetics are attributed to the increasing entropy, resulting in more dense lithium metal growth, despite the low concentration of HE electrolyte.


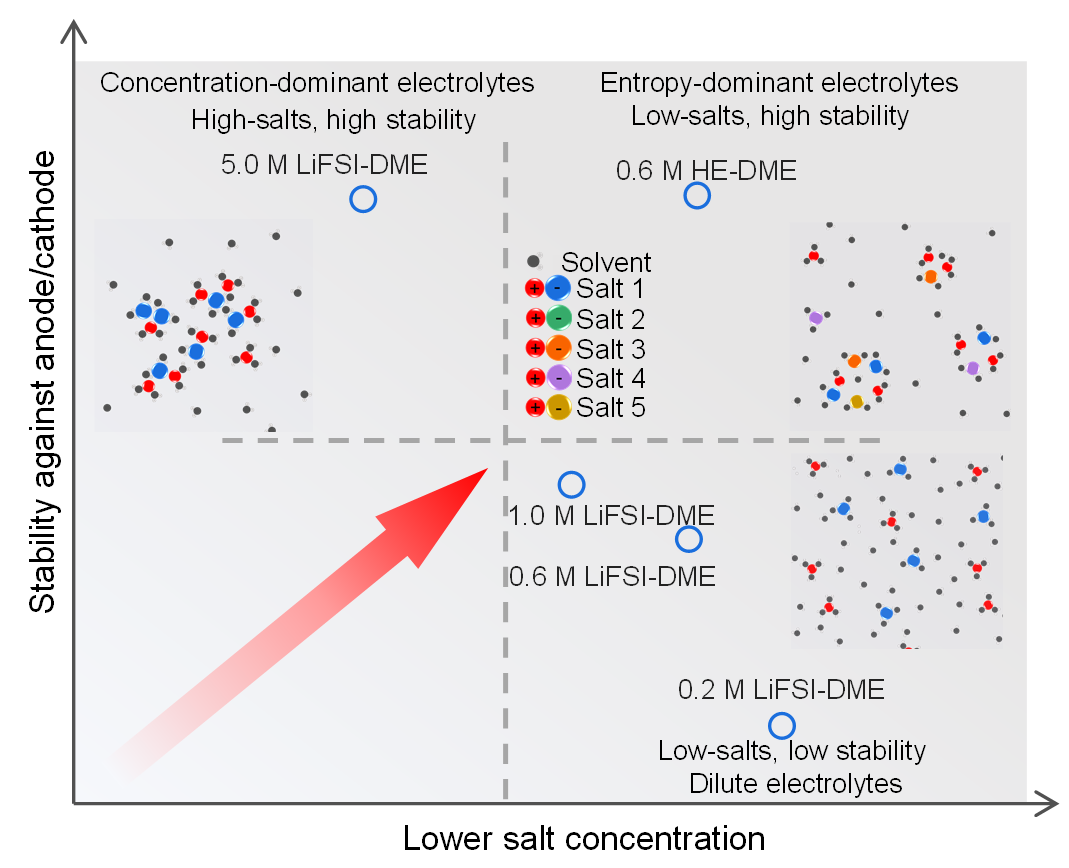


**Supplementary Fig. 83. Relationship between the** **salt concentration and stability against anode/cathode in liquid electrolytes.** Based on the above results, the characteristics of conventional dilute electrolytes, high salt concentration electrolytes and HE electrolytes are compared. From this comparison, the HE demonstrates promising assets, especially realizing improved stability against the anode/cathode in low salt concentration liquid electrolytes, typically achieved only with highly concentrated electrolytes.


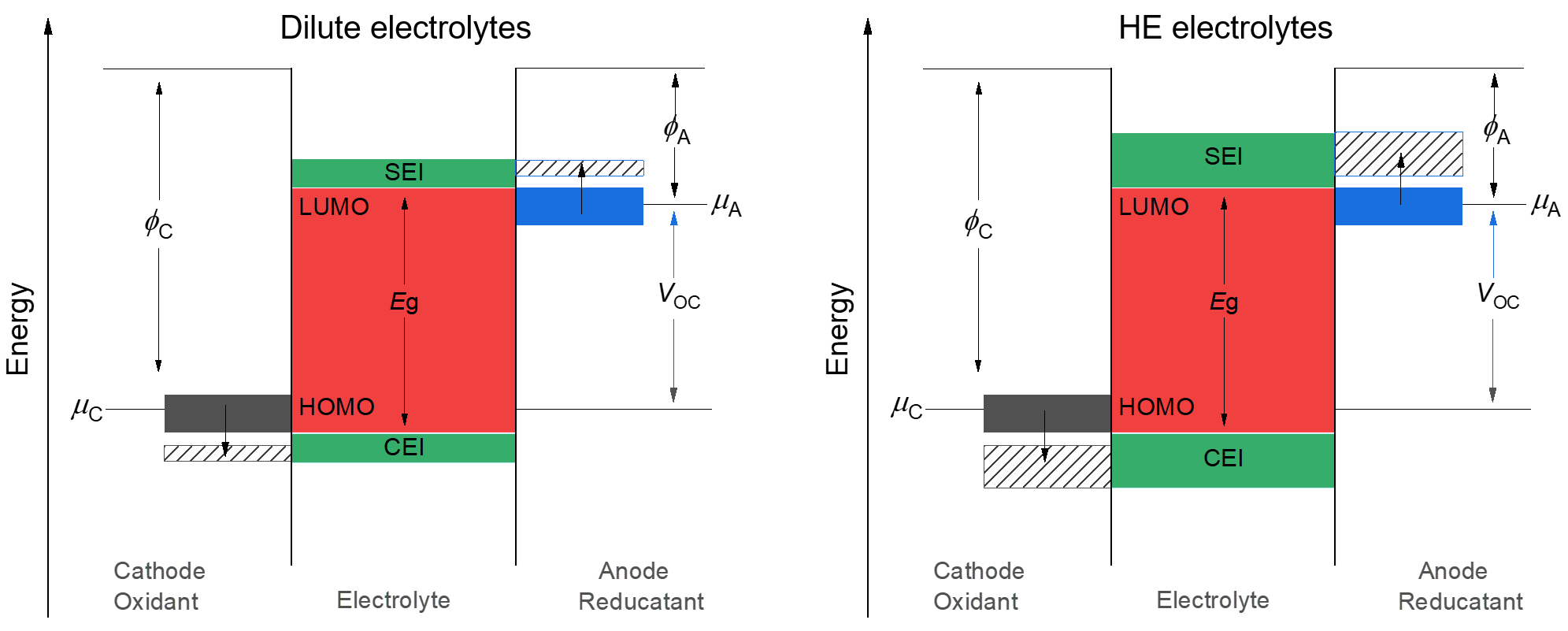


**Supplementary Fig. 84. Schematic diagram about formation of the SEI/CEI layer between electrodes and the liquid electrolyte in cells.** The anode with an electrochemical potential *μ*_A_ above the lowest unoccupied molecular orbital (LUMO) could reduce the electrolyte but the SEI formation could prevent electron transfer to electrolyte LUMO; cathode with electrochemical potential *μ*_C_ below the highest occupied molecular orbital (HOMO) could also oxidize the electrolyte but the CEI formation limits the electron transfer to the cathode from the electrolyte HOMO. Compared to the dilute electrolytes, the HE electrolyte with various kinds of salts could enhance the stabilities of both SEI and CEI layers, enlarging the voltage window of electrolytes.

**Supplementary Fig. 85. Optimization of a five-component 0.6 M HE electrolyte. a**, Lithium plating/stripping CE in Li||Cu cells using the four-component 0.6 M HE and the five-component 0.6 M HE electrolytes at 0.5 mA cm^−2^ (dis)charging each time to a capacity of 1 mAh cm^−2^. The five-component 0.6 M HE electrolyte, including 0.15 M LiFSI, 0.1 M LiTFSI, 0.1 M LiDFOB, 0.1 M LiBETI and 0.15 M LiNO_3_ in DME, was prepared to explore its performance in comparison with the four-component 0.6 M HE electrolyte. **b**, Galvanostatic lithium plating/stripping profiles of a Li||Cu cell for the five-component 0.6 M HE electrolyte at different cycles. **c**, Morphology of the deposited lithium metal from top-view SEM for the five-component 0.6 M HE electrolyte at a current density of 0.5 mA cm^-2^, charged to a capacity of 1 mAh cm^-2^ on Cu foil. **d**, Galvanostatic charge profiles of Li||LiFePO_4_ cells of the four and five component 0.6 M HE electrolytes at a current density of 0.02 C to study the oxidation stability of the electrolytes. **e,** Electrochemical rate capabilities and **f**, cycling performance of Li||NCM811 cells cycled in the voltage window from 2.8 to 4.3 V using the four and five component 0.6 M HE electrolytes.


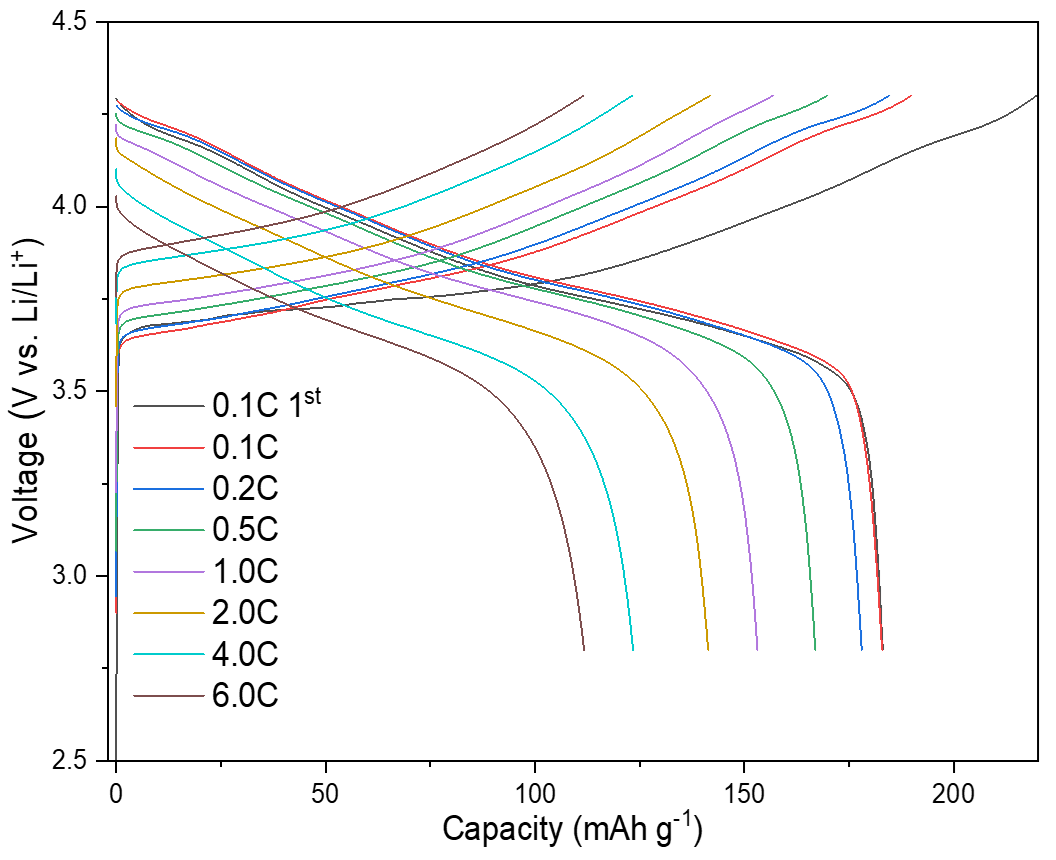


**Supplementary Fig. 86.** **Galvanostatic charge/discharge curves of Li||NCM811 cell in five-component 0.6 M HE-DME electrolyte at different rates.** The cells were cycled at 2.8-4.3 V.


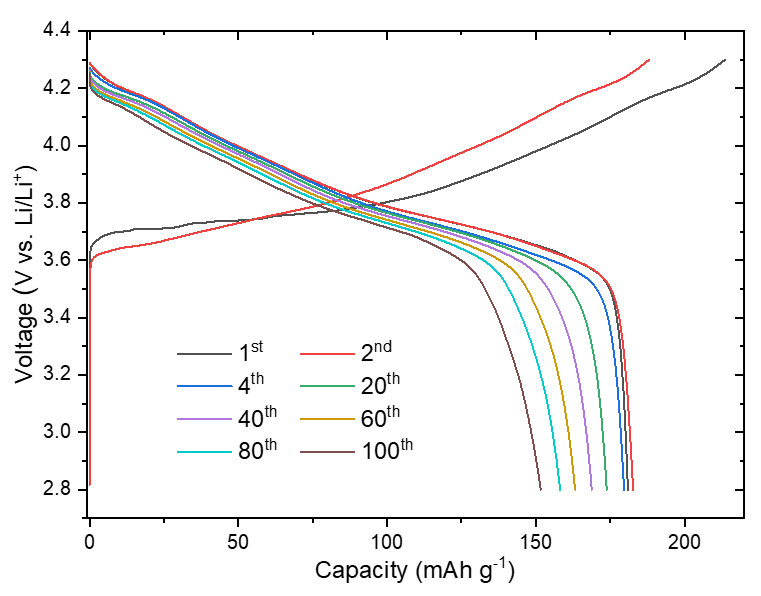


**Supplementary Fig. 87. Galvanostatic charge/discharge curves of Li||NCM811 cell in five-component 0.6 M HE-DME electrolyte at different cycles.** Cells were cycled at the voltage range of 2.8-4.3 V under a rate of 0.1C for three cycles before cycling at 0.333C rate.

**Supplementary Table 1.** Properties for 0.6 M LiFSI-DME, 5.0 M LiFSI-DME and 0.6 M HE-DME electrolytes at room temperature.

|  | *t*_Li_^+^ | Conductivity (mS cm^-1^) | *D*_s_ (10^-5^ cm^2^ s^-1^) |
| --- | --- | --- | --- |
| 0.6 M LiFSI-DME | ~0.39 | ~9.6 | 1.62 |
| 5.0 M LiFSI-DME | ~0.31 | ~1.9 | 0.28 |
| 0.6 M HE-DME | ~0.46 | ~10.2 | 1.96 |

**Supplementary Table 2.** The types of solvation structures and their corresponding proportion in the 0.6 M LiFSI-DME electrolyte.

| DME-LiFSI | Proportion |
| --- | --- |
| 2-1 | 0.51295 |
| 2-2 | 0.165241667 |
| 1-2 | 0.11296875 |
| 3-0 | 0.091239583 |
| 1-3 | 0.036404167 |
| Others | 0.081196 |

**Supplementary Table 3.** The types of solvation structures and their corresponding proportion in the 0.6 M HE-DME electrolyte.

| DME-LiFSI-LiTFSI-  LiDFOB-LiNO_3_ | Proportion | DME | LiFSI | LiTFSI | LiDFOB | LiNO_3_ |
| --- | --- | --- | --- | --- | --- | --- |
| 2-1-0-0-0 | 0.144806 | 2 | 1 | 0 | 0 | 0 |
| 2-0-0-0-1 | 0.139000 | 2 | 0 | 0 | 0 | 1 |
| 2-0-1-0-0 | 0.074173 | 2 | 0 | 1 | 0 | 0 |
| 2-0-0-2-0 | 0.065970 | 2 | 0 | 0 | 2 | 0 |
| 2-1-1-0-0 | 0.061083 | 2 | 1 | 1 | 0 | 0 |
| 3-0-0-0-0 | 0.060497 | 3 | 0 | 0 | 0 | 0 |
| 2-1-0-1-0 | 0.060212 | 2 | 1 | 0 | 1 | 0 |
| 2-0-0-0-2 | 0.051047 | 2 | 0 | 0 | 0 | 2 |
| 2-0-0-1-1 | 0.045853 | 2 | 0 | 0 | 1 | 1 |
| 3-0-0-0-1 | 0.036520 | 3 | 0 | 0 | 0 | 1 |
| 2-0-1-1-0 | 0.025692 | 2 | 0 | 1 | 1 | 0 |
| 2-1-0-0-1 | 0.024130 | 2 | 1 | 0 | 0 | 1 |
| 2-0-0-1-0 | 0.013062 | 2 | 0 | 0 | 1 | 0 |
| 2-0-0-0-0 | 0.013018 | 2 | 0 | 0 | 0 | 0 |
| 3-0-0-0-2 | 0.012802 | 3 | 0 | 0 | 0 | 2 |
| 1-0-0-0-2 | 0.012680 | 1 | 0 | 0 | 0 | 2 |
| 2-0-0-1-2 | 0.009469 | 2 | 0 | 0 | 1 | 2 |
| 1-1-0-0-1 | 0.008780 | 1 | 1 | 0 | 0 | 1 |
| 1-0-0-1-2 | 0.008232 | 1 | 0 | 0 | 1 | 2 |
| 3-1-0-0-0 | 0.008113 | 3 | 1 | 0 | 0 | 0 |
| 2-0-2-0-0 | 0.007466 | 2 | 0 | 2 | 0 | 0 |
| 3-0-0-1-0 | 0.007105 | 3 | 0 | 0 | 1 | 0 |
| 3-0-0-1-1 | 0.006357 | 3 | 0 | 0 | 1 | 1 |
| 2-0-1-0-1 | 0.005442 | 2 | 0 | 1 | 0 | 1 |
| 1-0-1-2-0 | 0.005358 | 1 | 0 | 1 | 2 | 0 |
| 1-0-0-0-1 | 0.005228 | 1 | 0 | 0 | 0 | 1 |
| 3-1-0-0-1 | 0.005087 | 3 | 1 | 0 | 0 | 1 |
| 1-2-0-0-0 | 0.005020 | 1 | 2 | 0 | 0 | 0 |
| 1-1-0-0-2 | 0.004825 | 1 | 1 | 0 | 0 | 2 |
| 3-0-1-0-0 | 0.004722 | 3 | 0 | 1 | 0 | 0 |
| 2-1-0-0-2 | 0.003660 | 2 | 1 | 0 | 0 | 2 |
| 1-1-1-1-0 | 0.003588 | 1 | 1 | 1 | 1 | 0 |
| 2-1-0-1-1 | 0.000679 | 2 | 1 | 0 | 1 | 1 |
| 2-1-1-0-1 | 0.000010 | 2 | 1 | 1 | 0 | 1 |
| 2-0-1-1-1 | 0.000001 | 2 | 0 | 1 | 1 | 1 |
| Others | 0.06 |  |  |  |  |  |

**Supplementary References**

1 Maier, J. Concentration Polarization of Salt-Containing Liquid Electrolytes. *Advanced Functional Materials* **21**, 1448-1455, doi:<https://doi.org/10.1002/adfm.201002153> (2011).

2 Wang, Q. *et al.* Interface chemistry of an amide electrolyte for highly reversible lithium metal batteries. *Nature Communications* **11**, 4188, doi:10.1038/s41467-020-17976-x (2020).

3 Weber, R. *et al.* Long cycle life and dendrite-free lithium morphology in anode-free lithium pouch cells enabled by a dual-salt liquid electrolyte. *Nature Energy* **4**, 683-689, doi:10.1038/s41560-019-0428-9 (2019).

4 Fang, C. *et al.* Pressure-tailored lithium deposition and dissolution in lithium metal batteries. *Nature Energy* **6**, 987-994, doi:10.1038/s41560-021-00917-3 (2021).

5 Rehnlund, D., Ihrfors, C., Maibach, J. & Nyholm, L. Dendrite-free lithium electrode cycling via controlled nucleation in low LiPF6 concentration electrolytes. *Materials Today* **21**, 1010-1018, doi:<https://doi.org/10.1016/j.mattod.2018.08.003> (2018).

6 Pei, A., Zheng, G., Shi, F., Li, Y. & Cui, Y. J. N. l. Nanoscale nucleation and growth of electrodeposited lithium metal. **17**, 1132-1139 (2017).

7 Bhattacharyya, R. *et al.* In situ NMR observation of the formation of metallic lithium microstructures in lithium batteries. *Nature Materials* **9**, 504-510, doi:10.1038/nmat2764 (2010).

8 Gunnarsdóttir, A. B., Amanchukwu, C. V., Menkin, S. & Grey, C. P. Noninvasive In Situ NMR Study of “Dead Lithium” Formation and Lithium Corrosion in Full-Cell Lithium Metal Batteries. *Journal of the American Chemical Society* **142**, 20814-20827, doi:10.1021/jacs.0c10258 (2020).

9 Küpers, V., Kolek, M., Bieker, P., Winter, M. & Brunklaus, G. In situ 7Li-NMR analysis of lithium metal surface deposits with varying electrolyte compositions and concentrations. *Physical Chemistry Chemical Physics* **21**, 26084-26094, doi:10.1039/C9CP05334D (2019).

10 Chandrashekar, S. *et al.* 7Li MRI of Li batteries reveals location of microstructural lithium. *Nature Materials* **11**, 311-315, doi:10.1038/nmat3246 (2012).

11 Chang, H. J. *et al.* Investigating Li Microstructure Formation on Li Anodes for Lithium Batteries by in Situ 6Li/7Li NMR and SEM. *The Journal of Physical Chemistry C* **119**, 16443-16451, doi:10.1021/acs.jpcc.5b03396 (2015).

12 Jang, D. H. & Oh, S. M. Electrolyte Effects on Spinel Dissolution and Cathodic Capacity Losses in 4 V Li / Li x Mn2 O 4 Rechargeable Cells. *Journal of The Electrochemical Society* **144**, 3342-3348, doi:10.1149/1.1838016 (1997).

13 Suo, L. *et al.* Fluorine-donating electrolytes enable highly reversible five-V-class Li metal batteries. *Proceedings of the National Academy of Sciences* **115**, 1156, doi:10.1073/pnas.1712895115 (2018).

14 Wang, C., Meng, Y. S. & Xu, K. Perspective—Fluorinating Interphases. *Journal of The Electrochemical Society* **166**, A5184-A5186, doi:10.1149/2.0281903jes (2018).

15 Krause, L. J. *et al.* Corrosion of aluminum at high voltages in non-aqueous electrolytes containing perfluoroalkylsulfonyl imides; new lithium salts for lithium-ion cells. *Journal of Power Sources* **68**, 320-325, doi:<https://doi.org/10.1016/S0378-7753(97)02517-2> (1997).
